# Group Interpersonal Psychotherapy for Depression in Perinatal Adolescents in Kenya: A Pilot Randomized Clinical Trial

**DOI:** 10.1001/jamanetworkopen.2026.18255

**Published:** 2026-06-16

**Authors:** Manasi Kumar, Albert Tele, Vincent Nyongesa, Obadia Yator, Shillah Mwaniga Mwavua, Joseph Kathono, Darius Nyamai, Angela Langat, Carol Ngunu, Anne Obondo, Keng Yen Huang, Caleb Othieno

**Affiliations:** 1 Institute for Excellence in Health Equity, Department of Population Health, New York University School of Medicine, New York, New York; 2 Faculty of Behavioural and Movement Sciences, Department of Clinical, Neuro Developmental Psychology, Vrije University Amsterdam, Amsterdam, The Netherlands; 3 Department of Psychiatry, University of Nairobi, Nairobi, Kenya; 4 UNITID (University of Nairobi Institute of Tropical and Infectious Disease), Nairobi, Kenya; 5 Nairobi City County Government, Health Wellness and Nutrition, Nairobi, Kenya; 6 Center for Epidemiological Modelling and Analysis, University of Nairobi, Nairobi, Kenya; 7 Department of Psychiatry, Maseno University, Kisumu, Kenya

## Abstract

**Importance:**

Group interpersonal psychotherapy (IPT-G) is effective across diverse populations and adaptable to low-resource settings.

**Objective:**

To assess whether shortened 4-session mini IPT-G vs full IPT-G delivered by lay health care workers reduces depression and improves family functioning among perinatal adolescents.

**Design, Setting, and Participants:**

This pilot randomized clinical trial of group IPT for depression used a 3-arm longitudinal type 1 implementation-effectiveness design between May 13, 2022, and April 1, 2024, at 2 primary care maternal and child health clinics in Nairobi, Kenya. The study applied a collaborative care approach within the World Health Organization (WHO) Mental Health Gap Action Programme framework for training a nonspecialist workforce, where trained nurses screened participants and supervised group sessions delivered by community health promoters collaboratively with psychologists. Participants included perinatal adolescents, aged 13 to 18 years, in their first to second trimester of pregnancy.

**Intervention:**

Participants were randomized to treatment as usual (TAU [n = 44]), mini IPT-G (n = 38), or full IPT-G (n = 40). TAU involved information sheets; mini IPT-G was culturally adapted; and full IPT-G followed the WHO protocol.

**Main Outcomes and Measures:**

Primary outcomes included depression measured by the 9-item Patient Health Questionnaire (PHQ-9) and functional impairment within 1 week post intervention and at 6-month follow-up. Secondary outcomes were posttraumatic stress disorder, psychological distress, disability, and self-efficacy. Linear mixed models were used to evaluate the preliminary effectiveness of the interventions.

**Results:**

Among the 122 adolescents included in the analysis (median age, 17 [IQR, 17-18] years), 97 (79.5%) were single, and 91 (74.6%) had at least a secondary education level. Retention included 101 participants (82.8%) at 1 week post intervention and 91 (74.6%) at 6-month follow-up. Both IPT arms demonstrated significantly greater reductions in depression than the TAU arm post intervention. Mean (SD) PHQ-9 score decreased from 12.38 (5.90) at baseline to 2.15 (1.94) in the full IPT-G arm (β vs TAU = −5.79; 95% CI, −7.67 to −3.91; *P* < .001), from 11.58 (4.93) to 3.94 (2.81) in the mini IPT-G arm (β vs TAU = −3.97; 95% CI −5.83 to −2.10; *P* < .001), and from 11.00 (4.97) to 7.94 (3.71) in the TAU arm. At 6 months, effects were attenuated. Scores in the full IPT-G arm remained significantly lower than in the TAU arm (β = −2.22; 95% CI, −4.25 to −0.18; *P* = .03) but not in the mini IPT-G arm (β = −1.38; 95% CI, −3.25 to 0.48; *P* = .15).

**Conclusions and Relevance:**

In this trial, IPT-G delivered by nonspecialists was feasible, acceptable, and effective. Mini IPT-G improved completion rates, while full IPT-G showed more sustained benefits at follow-up. These findings support task-sharing of mental health interventions for perinatal adolescents in low-resource settings.

**Trial Registration:**

Pan African Clinical Trial Registry Identification: PACTR202501888041900

## Key Points

**Question:**

How effective is task-shifted group interpersonal psychotherapy (IPT-G) for depression management in Kenya, and how do full vs mini versions fare with perinatal adolescents?

**Findings:**

In this randomized clinical trial of 122 participants, both full and mini IPT-G significantly reduced depressive symptoms compared with treatment as usual within 1 week post intervention, with full IPT-G demonstrating greater reductions than mini IPT-G over time.

**Meaning:**

These findings suggest that contextually adapted IPT-G, delivered via task-shifting, is an effective and scalable approach for reducing perinatal adolescent depression in low-resource settings.

## Teaser

This randomized clinical trial evaluates the feasibility and preliminary effectiveness of task-shifted delivery of full and mini versions of group interpersonal psychotherapy for depression among perinatal adolescents in Kenya.

## Introduction

In Kenya, as in other low- and middle-income countries (LMICs), a substantial treatment gap persists for common mental disorders in perinatal adolescents.^1^ Depressive disorders remain a leading cause of mental disability, and their burden is high in adolescents in Kenya.^2^ This gap triggers additional vulnerabilities and stressors, including discontinuation of education, poor skills training, social isolation, and financial insecurities. Vulnerable groups of perinatal adolescents experience a high prevalence of depression and compounded social adversities, which add to considerable barriers to accessing care.^3^

Vulnerable populations frequently lack access to resources that address both individual and community challenges, increasing their risk of harm and neglect.^4^ Vulnerability results from accumulation of social, psychological, and physical challenges over time, heightening susceptibility to adverse health outcomes.^5^ Social barriers further complicate health care access for these groups.^4^ Economic and social inequities impede access to medical care,^6^ placing these populations at elevated risk of morbidity and mortality.^7^ Despite a greater need, these groups are less likely to seek or use health services.^5^

Poverty, gender-based violence, and early pregnancy- and parenting-related stress collectively perpetuate a cycle of vulnerability. This cycle adversely affects both maternal and child health outcomes.^8^ The World Health Organization’s Mental Health Gap Action Programme^9^ provides a framework for integrating mental health services into primary health systems. Task-shifting approaches enable nonspecialist health workers to play a vital role in detecting, treating, and supporting individuals with common mental disorders.^10^ Several interventions have been recommended within this program, including group interpersonal psychotherapy (IPT-G).^11^

Interpersonal psychotherapy (IPT) is an evidence-based, time-limited psychological intervention targeting interpersonal difficulties contributing to depressive symptoms.^12,13,14^ After conducting an engagement interview and taking a patient history, the therapist and patient define an interpersonal problem through the lens of 4 problem areas: grief, interpersonal disputes, role transitions, and interpersonal or social deficits.^15,16,17,18^ The individual version of the therapy is delivered within 12 to 16 sessions, with 8 sessions recommended in the World Health Organization IPT-G manual. Irrespective of the modality, the therapy covers 3 phases: initial, middle, and termination.^12,19,20^ IPT has demonstrated efficacy across diverse populations and settings, including LMICs,^21,22,23^ and can be adapted to local cultural and resource contexts. The group format of IPT-G provides additional benefits in low-resource settings by enhancing peer support and reducing reliance on limited specialist health workers.^24^ Although outcomes in other studies were promising in high-income countries with pregnant women, they left several gaps that we aimed to fill in the present study, which includes pregnant adolescents rather than adults, uses 2 briefer group versions of IPT, and provides replication in a low-income country.

Implementing psychological interventions through task-shifting to nonspecialized health workers is a critical strategy to address this public health need.^25^ The standard IPT protocol, consisting of 8 to 12 sessions, presents a significant barrier to scale-up. A key unresolved question is whether a condensed version of IPT can retain most of its clinical benefits while improving feasibility and demonstrating clinical effectiveness of full-IPT-G. One of our hypothesis and implementation questions was whether most symptomatic improvement occurs during initial sessions, in which case a shorter protocol may balance efficacy and efficiency and thereby enhance scalability. The full protocol could therefore be optimized for more severe symptoms, while a shorter version could be scaled up for mild to moderate depression.

This study was designed to evaluate feasibility and preliminary effectiveness of an integrated IPT-G delivery in Kenya. We combined Mental Health Gap Action Programme–based screening by nurses with nonspecialist delivery by community health promoters (CHPs) of IPT-G. The primary objective was to compare standard 8-session full IPT-G with a newly developed, condensed 4-session mini IPT-G. Both interventions were evaluated against an enhanced treatment as usual (TAU) control for pregnant adolescents with depression. Several publications have informed this work.^24,26,27,28^ The study sought to determine whether a mini IPT-G protocol could achieve a substantial proportion of clinical benefit. If successful, mini IPT-G could serve as a more scalable first-line treatment, increasing access to evidence-based psychological care without significant loss of effectiveness.

## Methods

### Design

We conducted a 3-arm pilot randomized type 1 hybrid implementation-effectiveness trial to compare the implementation and outcomes of a brief vs a full IPT-G protocol. Participants were randomized into 3 intervention arms: full IPT-G, mini IPT-G, and TAU. The trial protocol is found in Supplement 1. The protocol was approved by the Kenyatta National Hospital–University of Nairobi Institutional Review Board. All procedures were conducted in accordance with the Declaration of Helsinki.^29^ All participants provided written informed consent. This study followed the Consolidated Standards of Reporting Trials (CONSORT) reporting guideline.

### Participants

**Figure 1.**
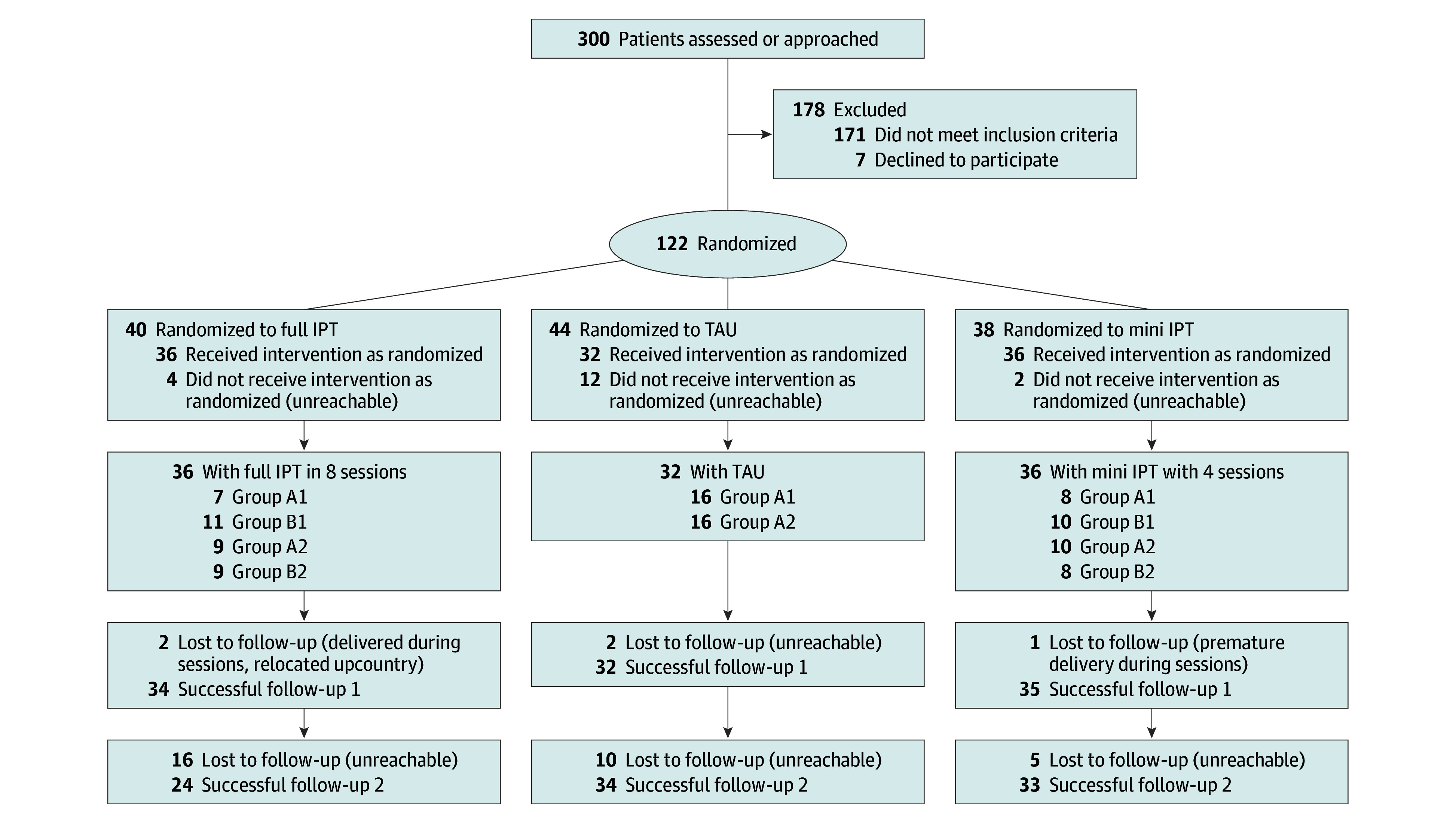
CONSORT Flow Diagram Exclusion criteria consisted of current suicidal ideation with an active plan or a plan within the past 3 months, suicide attempts in the past 3 months, ongoing alcohol or substance abuse, severe cognitive or physical impairments limiting participation, or severe mental disorders such as bipolar disorder or schizophrenia. IPT indicates interpersonal psychotherapy; full, 8-session group IPT; mini, 4-session group IPT; and TAU, treatment as usual.

Pregnant adolescents (n = 122) aged 13 to 18 years were identified using purposive and snowball sampling with support from CHPs. Eligibility and inclusion criteria included fluency in English or Kiswahili, provision of written informed consent, agreement to participate in intervention sessions, and a score of 10 or greater on the Edinburgh Postnatal Depression Scale,^30^ indicating depressive symptoms. Exclusion criteria consisted of current suicidal ideation with an active plan or a plan within the past 3 months, suicide attempts in the past 3 months, ongoing alcohol or substance abuse, severe cognitive or physical impairments limiting participation, or severe mental disorders such as bipolar disorder or schizophrenia (Figure 1).

#### Trial Design and Comparison Arms

Participants in the TAU arm received psychoeducational materials on depression, anxiety, and substance use after baseline assessment, as well as routine postnatal follow-up calls. Participants in the full IPT-G arm attended 1 pregroup individual session and 8 weekly 90-minute group sessions. Participants in the mini IPT-G arm attended 1 pregroup individual session and 4 weekly 90-minute group sessions (eTable 1 in Supplement 2).

#### Consent and Enrollment

During the first interaction, participants received study information sheets and consent forms. At the second meeting, facilitators collected signed consent forms, provided further study information, and confirmed participant understanding. All adolescents, including those younger than 18 years, provided written informed consent as emancipated minors, without requiring parental or guardian consent.

### Setting

The study was conducted between May 13, 2022, and April 1, 2024, at Kariobangi North and Kangemi health centers, which are under the Nairobi County Council. Kariobangi serves a low-income residential area in northeastern Nairobi, with a population of 18 903 residents.^31^ Kangemi is located in a tenement area on the outskirts of Nairobi, serving 116 710 residents.^31^

### Intervention

#### Development and Adaptation

The intervention consisted of 2 IPT-G formats: a full IPT-G (8 sessions) and a mini IPT-G (4 sessions).^26^ Intervention manuals and procedures were culturally and contextually adapted to the needs of adolescents in the study setting, with attention to the unique challenges faced by pregnant and parenting adolescents.^24^ Content adaptation and refinement for cultural relevance and developmental appropriateness were completed by a multidisciplinary team of clinical psychologists, public health researchers, and certified IPT supervisors.

#### Intervention Procedures

Participants completed a pre–group engagement interview to build rapport, conduct an interpersonal inventory, and identify a primary problem area, such as grief, role transition, or interpersonal dispute. Participants received psychoeducation on the link between interpersonal events and mood, and logistical barriers to participation were addressed. Group sessions were cofacilitated by 2 CHPs and followed the 3 IPT phases: initial, middle, and termination. Sessions began with review of the previous week’s mood using the 9-item Patient Health Questionnaire (PHQ-9), followed by structured discussions and exercises targeting identified problem areas with standard IPT techniques. Groups consisted of 7 to 11 participants. Details about facilitator training and supervision are available in eMethods 1 in Supplement 2.

### Randomization

Eligible participants were randomized to the 3 arms using a computer-generated sequence prepared by a member of the research team (A.T.) who did not participate in recruitment or intervention delivery. Randomization was implemented in blocks of equal size (45 per arm and 135 participants per cohort) to balance across arms. Allocation was concealed using sealed, opaque envelopes that were sequentially numbered and opened only after the participant’s baseline assessment was completed. However, other participants declined, leaving a sample of 122.

### Outcome Measurements

Mental health outcomes were assessed before the first individual session (baseline), within 1 week after intervention completion (post intervention), and at 6 months after the intervention (follow-up). Primary outcomes included depression, measured with the PHQ-9, a 9-item self-report scale based on the *Diagnostic and Statistical Manual of Mental Disorders* (Fifth Edition)^32,33^ criteria for screening and measuring the severity of depressive symptoms.^32,34^ Functional impairment was measured with a United Nations Children’s Fund–validated tool,^35,36^ a 3-item scale measuring the frequency of emotional or psychological interference in home, school or work, and peer functioning during the past month, with higher summed scores (range, 0-9) indicating greater impairment. Secondary outcomes were measured using validated instruments assessing well-being, functioning, coping, self-efficacy, social support, trauma symptoms, and interpersonal violence (eTable 2 in Supplement 2).

### Quantitative Data Collection and Analysis

Sociodemographic characteristics were collected through structured interviews at baseline. Trained research assistants administered all interviews using tablets with Dooblo software, version V628,^37^ to ensure standardized and secure data collection.

### Statistical Analysis

Descriptive statistics summarized participants’ baseline sociodemographic and mental health outcomes across the 3 arms. For continuous variables, normality was assessed using the Shapiro-Wilk test and visual inspection of Q-Q plots. Normally distributed variables (eg, baseline PHQ-9 scores, age) are reported as means and SDs. Nonnormally distributed variables (eg, number of sessions attended) are reported as medians with IQRs and ranges (minimum to maximum). Categorical variables (eg, intervention arm, sex) are reported as frequencies and percentages. Baseline differences between intervention arms were assessed using 1-way analysis of variance for continuous variables and χ^2^ tests for categorical variables to evaluate group comparability prior to intervention.

For longitudinal outcomes, linear mixed-effects models were fitted separately for each measure. Models included fixed effects for time (baseline, post intervention, and follow-up), intervention group, and their interaction, with a random intercept for participant identification to account for repeated measures. Estimated marginal means were used to estimate group differences post intervention and at follow-up. Cohen *d* effect sizes were calculated as the difference in estimated marginal means divided by pooled baseline SD, with 95% CIs for each comparison. Analyses were conducted using Stata, version 18 (StataCorp LLC), with statistical significance set at 2-sided *P* < .05 (eMethods 2-10 and eTable 3 in Supplement 2).

## Results

Eligibility screening identified 300 individuals, of whom 178 (59.3%) were excluded: 171 (57.0%) did not meet inclusion criteria, and 7 (2.3%) declined participation. The remaining 122 participants (40.7%) were randomized to full IPT-G (40 [32.8%]), TAU (44 [36.1%]), or mini IPT-G (38 [31.1%]). In the full IPT-G arm, 36 participants (90.0%) received the intervention, and 4 (10.0%) were unreachable. In the TAU arm, 32 participants (72.7%) received the intervention, and 12 (27.3%) were unreachable. In the mini IPT-G arm, 36 participants (94.7%) received the intervention, and 2 (5.3%) were unreachable. Within 1 week post intervention, 34 participants (85.0%) in the full IPT-G arm, 32 (72.7%) in the TAU arm, and 35 (92.1%) in the mini IPT-G arm completed assessments. At 6 months, 24 participants (60.0%) in the full IPT-G arm, 34 (77.3%) in the TAU arm, and 33 (86.8%) in the mini IPT-G arm completed follow-up assessments (Figure 1).

Baseline demographic and psychosocial characteristics are summarized in Table 1 and eTable 4 in Supplement 2. Median participant age was 17 (IQR, 17-18 years), and 75 (61.5%) were younger than 18 years. Most participants were single (97 [79.5%]) and had completed at least secondary education (91 [74.6%]). Most participants reported low household income (<KSh 4999 per month, 64 [52.5%]), with half (61 [50.0%]) living with their parents. Baseline PHQ-9 score indicated moderate depressive symptoms across groups (full IPT-G: 12.38 [5.90]; mini IPT-G: 11.58 [4.93]; TAU: 11.00 [4.97]). Functional limitations and other psychosocial measures were comparable across study arms. No statistically significant baseline differences were observed between groups.

**Table 1.** Baseline Characteristics of Intention to Treat Population

Characteristic	Study arm, No. (%) of participants			
	TAU (n = 44)	Mini IPT (n = 38)	Full IPT (n = 40)	All (N = 122)
Age, y				
<18	26 (59.1)	23 (60.5)	26 (65.0)	75 (61.5)
18	18 (40.9)	15 (39.5)	14 (35.0)	47 (38.5)
Age, median (IQR), y	17 (17-18)	17 (17-18)	17 (17-18)	17 (17-18)
Marital status				
Single	37 (84.1)	27 (71.1)	33 (82.5)	97 (79.5)
Married or partnered	7 (15.9)	11 (28.9)	7 (17.5)	25 (20.5)
Highest educational attainment				
Primary school	13 (29.5)	12 (31.6)	6 (15.0)	31 (25.4)
Secondary or high school	31 (70.5)	26 (68.4)	34 (85.0)	91 (74.6)
Mean family monthly income, KSh^a^				
<4999	24 (54.5)	23 (60.5)	17 (42.5)	64 (52.5)
5000-9999	14 (31.8)	10 (26.3)	17 (42.5)	41 (33.6)
≥10 000	6 (13.6)	5 (13.2)	6 (15.0)	17 (13.9)
Persons sharing home				
Parents	24 (54.5)	14 (36.8)	23 (57.5)	61 (50.0)
Spouse or partner	7 (15.9)	10 (26.3)	7 (17.5)	24 (19.7)
Others	13 (29.5)	14 (36.8)	10 (25.0)	37 (30.3)
Currently using any medication				
No	38 (86.4)	31 (81.6)	38 (95.0)	107 (87.7)
Yes	6 (13.6)	7 (18.4)	2 (5.0)	15 (12.3)
Weeks of gestation at first ANC visit				
<12 (<3 mo)	10 (22.7)	13 (34.2)	16 (40.0)	39 (32.0)
12-28 (3-7 mo)	31 (70.5)	25 (65.8)	23 (57.5)	79 (64.8)
>28 (>7 mo)	3 (6.8)	0	1 (2.5)	4 (3.3)
Unplanned pregnancy				
No	20 (45.5)	23 (60.5)	10 (25.0)	53 (43.4)
Yes	24 (54.5)	15 (39.5)	30 (75.0)	69 (56.6)
Presence of social support				
No	6 (13.6)	6 (15.8)	3 (7.5)	15 (12.3)
Yes	38 (86.4)	32 (84.2)	37 (92.5)	107 (87.7)
Family member ever treated for mental illness (eg, depression)				
No	42 (95.5)	29 (76.3)	33 (82.5)	104 (85.2)
Yes	2 (4.5)	9 (23.7)	7 (17.5)	18 (14.8)
Ever experienced intimate partner violence in pregnancy				
No	35 (79.5)	30 (78.9)	33 (82.5)	98 (80.3)
Yes	9 (20.5)	8 (21.1)	7 (17.5)	24 (19.7)
Lives with individual with problem alcohol use or street drug use				
No	33 (75.0)	22 (57.9)	30 (75.0)	85 (69.7)
Yes	11 (25.0)	16 (42.1)	10 (25.0)	37 (30.3)
Ever consumed alcohol at any time in your life				
No	33 (75.0)	24 (63.2)	25 (62.5)	82 (67.2)
Yes	11 (25.0)	14 (36.8)	15 (37.5)	40 (32.8)
Pressured into substance use by friends or spouse				
No	34 (77.3)	22 (57.9)	29 (72.5)	85 (69.7)
Yes	10 (22.7)	16 (42.1)	11 (27.5)	37 (30.3)

Abbreviations: ANC, antenatal care; IPT, group interpersonal psychotherapy; KSh, Kenyan Shillings; TAU, treatment as usual.

^a^ US $1 = KSh 123.

### Feasibility of IPT and Study Procedures

#### Participation, Attendance, and Treatment Completion

In the full IPT-G arm, participants attended a mean (SD) of 6.5 (2.2) of 8 sessions. In the mini IPT-G arm, participants attended a mean (SD) of 3.6 (0.8) per 4 sessions (eTable 5 in Supplement 2).

Retention meant attendance at the termination session and included 101 participants (82.8%) post intervention and 91 (74.6%) at 6-month follow-up. Treatment completion meant attending all scheduled sessions. Retention was comparable between arms (full IPT-G: 28 [77.8%]; mini IPT-G: 32 [88.9%]). However, treatment completion was higher for the mini IPT-G arm (28 [77.8%]) than for the full IPT-G arm (19 [52.8%]) (eFigure 1 in Supplement 2).

#### Implementation Success

Both treatment arms demonstrated high fidelity to the intervention protocol, reflecting strong adherence to the core principles and delivery structure of IPT-G. Facilitator competence was slightly higher in the mini IPT-G arm, with 98.5% of sessions rated as adequate or above on IPT fidelity checklist, compared with 92.0% for full IPT-G. CHPs delivered both protocols effectively. The mini IPT-G protocol contributed to improved consistency and focus during session delivery.

### Overview of Outcome Trends

**Figure 2.**
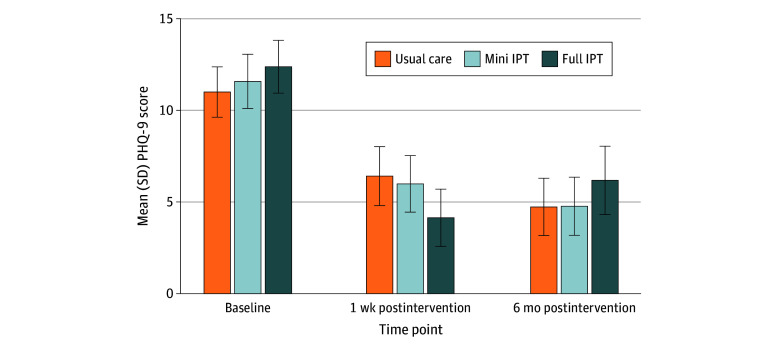
Bar Graph of 9-Item Patient Health Questionnaire Scores Over Time Indicates primary outcome. IPT indicates interpersonal psychotherapy; full, 8-session group IPT; and mini, 4-session group IPT.

Table 2, Figure 2, and eFigures 2 to 10 in Supplement 2 present overall trends in primary and secondary outcomes over time. Across all arms, depressive symptoms decreased from baseline to within 1 week post intervention, with smaller changes observed during follow-up.

**Table 2.** Mean Scores and Effect Sizes for Key Psychosocial Outcomes at Baseline, Post Intervention, and at Follow-Up by Intervention Arm

Measurement point	Study arm								
	TAU			Mini-IPT-G			Full-IPT-G		
	No. of participants	Mean (SD) score	Cohen *d* (95% CI)	No. of participants	Mean (SD) score	Cohen *d* (95% CI)	No. of participants	Mean (SD) score	Cohen *d* (95% CI)
PHQ-9 (depression)^a^									
Baseline	44	11.00 (4.97)	1 [Reference]	38	11.58 (4.93)	1 [Reference]	40	12.38 (5.90)	1 [Reference]
Post intervention	32	7.94 (3.71)	0.781 (0.269 to 1.293)	35	3.94 (2.81)	1.816 (1.256 to 2.377)	34	2.15 (1.94)	2.507 (1.867 to 3.146)
Follow-up	34	5.53 (2.26)	1.392 (0.858 to 1.925)	33	4.15 (3.21)	1.797 (1.222 to 2.373)	24	3.38 (2.36)	2.188 (1.466 to 2.911)
Functioning^b^									
Baseline	44	2.47 (0.74)	1 [Reference]	38	2.48 (0.71)	1 [Reference]	40	2.52 (0.7)	1 [Reference]
Post intervention	32	2.07 (0.71)	0.651 (0.144 to 1.157)	35	2.05 (0.73)	0.534 (0.054 to 1.014)	34	2.26 (0.63)	0.458 (−0.027 to 0.943)
Follow-up	34	2.45 (0.35)	0.097 (−0.382 to 0.576)	33	2.41 (0.5)	0.14 (−0.346 to 0.627)	24	2.38 (0.38)	0.19 (−0.383 to 0.763)
NSESSS-PTSD adult^c^									
Baseline	44	8.91 (9.33)	1 [Reference]	38	10.24 (11.42)	1 [Reference]	40	9.60 (9.95)	1 [Reference]
Post intervention	32	3.66 (6.78)	0.647 (0.141 to 1.154)	35	8.69 (9.13)	0.078 (−0.394 to 0.55)	34	6.29 (7.62)	0.364 (−0.119 to 0.846)
Follow-up	34	4.32 (6.91)	0.492 (0.006 to 0.978)	33	4.82 (6.68)	0.557 (0.062 to 1.052)	24	5.17 (7.46)	0.417 (−0.161 to 0.994)
HITS (intimate partner violence)^d^									
Baseline	44	7.05 (3.68)	1 [Reference]	38	7.47 (4.55)	1 [Reference]	40	7.15 (3.59)	1 [Reference]
Post intervention	32	5.62 (1.64)	0.583 (0.079 to 1.087)	35	6.17 (2.39)	0.312 (−0.163 to 0.787)	34	6.59 (2.03)	0.167 (−0.312 to 0.647)
Follow-up	34	6.18 (1.78)	0.399 (−0.085 to 0.882)	33	6.76 (2.86)	0.159 (−0.328 to 0.645)	24	7.25 (3.25)	−0.253 (−0.827 to 0.321)
CORE-10^e^									
Baseline	44	15.52 (8.07)	1 [Reference]	38	16.37 (5.81)	1 [Reference]	40	16.65 (6.3)	1 [Reference]
Post intervention	32	9.81 (5.76)	0.998 (0.474 to 1.521)	35	8.31 (5.17)	1.371 (0.847 to 1.896)	34	6.47 (4.43)	1.940 (1.360 to 2.521)
Follow-up	34	10.47 (5.14)	0.940 (0.436 to 1.445)	33	9.64 (6.36)	1.012 (0.496 to 1.528)	24	10.17 (5.43)	1.056 (0.445 to 1.666)
Brief COPE Inventory^f^									
Baseline	44	16.07 (4.43)	1 [Reference]	38	15.66 (3.78)	1 [Reference]	40	14.05 (4.01)	1 [Reference]
Post intervention	32	18.47 (4.23)	−0.572 (−1.075 to −0.068)	35	17.43 (4.49)	−0.445 (−0.923 to 0.032)	34	18.38 (5.12)	−1.004 (−1.512 to −0.496)
Follow-up	34	15.44 (2.99)	0.159 (−0.321 to 0.638)	33	16.76 (3.97)	−0.396 (−0.887 to 0.095)	24	15.46 (4.5)	−0.465 (−1.044 to 0.114)
General Self-Efficacy Scale^g^									
Baseline	44	27.41 (8.32)	1 [Reference]	38	26.16 (8.39)	1 [Reference]	40	25.2 (8.56)	1 [Reference]
Post intervention	32	28.16 (5.72)	0.041 (−0.453 to 0.534)	35	28.66 (7.67)	−0.280 (−0.754 to 0.194)	34	29.03 (5.98)	−0.626 (−1.117 to 0.136)
Follow-up	34	29.56 (4.1)	−0.330 (−0.812 to 0.152)	33	28.64 (6.44)	−0.356 (−0.846 to 0.134)	24	28.33 (5.21)	−0.522 (−1.103 to 0.059)
MSPSS^h^									
Baseline	44	4.27 (1.25)	1 [Reference]	38	3.95 (1.24)	1 [Reference]	40	4.06 (1.2)	1 [Reference]
Post intervention	32	4.77 (1.22)	−0.372 (−0.870 to 0.126)	35	4.53 (0.99)	−0.431 (−0.908 to 0.046)	34	4.67 (1.15)	−0.562 (−1.050 to −0.074)
Follow-up	34	4.50 (1.02)	−0.240 (−0.720 to 0.241)	33	4.70 (1.09)	−0.682 (−1.182 to −0.182)	24	4.36 (0.97)	−0.353 (−0.928 to 0.223)
WHODAS 2.0 (Short Version)^i^									
Baseline	44	23.82 (16.78)	1 [Reference]	38	23.14 (17.32)	1 [Reference]	40	26.67 (17.82)	1 [Reference]
Post intervention	32	13.87 (11.37)	0.832 (0.317 to 1.346)	35	16.96 (14.39)	0.399 (−0.078 to 0.875)	34	14.03 (12.97)	0.804 (0.306 to 1.301)
Follow-up	34	11.7 (10.95)	0.824 (0.326 to 1.323)	33	10.98 (14.06)	0.796 (0.291 to 1.301)	24	15.36 (15.01)	0.538 (−0.044 to 1.120)
WHO-5 Well-Being Index^j^									
Baseline	44	31.64 (22.39)	1 [Reference]	38	31.68 (22.38)	1 [Reference]	40	28.60 (16.47)	1 [Reference]
Post intervention	32	46.12 (30.72)	−0.606 (−1.111 to −0.101)	35	50.17 (24.10)	−0.743 (−1.231 to −0.255)	34	57.18 (25.19)	−1.351 (−1.882 to −0.821)
Follow-up	34	48.35 (27.77)	−0.815 (−1.313 to −0.317)	33	60.85 (26.88)	−1.077 (−1.597 to −0.557)	24	58.67 (28.00)	−1.329 (−1.96 to −0.697)

Abbreviations: COPE, Coping Orientation Problems Experiences; CORE-10, 10-item Clinical Outcomes in Routine Evaluation; HITS, Hurt, Insult, Threaten, Scream; IPT-G, group interpersonal therapy; MSPSS, Multidimensional Scale of Perceived Social Support; NSESSS-PTSD, Severity of Posttraumatic Stress Symptoms—Adult National Stressful Events Survey PTSD Short Scale; PHQ-9, 9-item Patient Health Questionnaire; TAU, treatment as usual; WHO-5 Well Being Index, World Health Organization 5-item Well Being Index; WHODAS 2.0, World Health Organization Disability Assessment Schedule 2.0.

^a^ Scores range from 0 to 27, with higher scores indicating increased depression severity.

^b^ Measured using a 3-item scale for the frequency of emotional or psychological interference in home, school, or work and peer functioning during the past month. Scores range from 0 to 9, with higher scores indicating greater impairment.

^c^ Scores range from 0 to 36, with higher scores indicating greater severity of posttraumatic stress disorder.

^d^ Scores range from 4 to 20, with higher scores indicating existence of intimate partner violence.

^e^ Scores range from 0 to 40, with higher scores indicating greater distress.

^f^ Scores range from 28 to 112, with higher scores indicating greater use of coping strategy.

^g^ Scores range from 10 to 40, with higher scores indicating greater self-efficacy.

^h^ Scores range from 12 to 84, with higher scores indicating high perceived support.

^i^ Scores range from 0 to 48, with higher scores indicating worse functioning.

^j^ Scores range from 0 to 25, with higher scores indicating best possible well-being.

### Primary Outcomes

Both IPT-G interventions reduced depressive symptoms compared with TAU within 1 week post intervention (mean [SD] PHQ-9 scores: 2.15 [1.94] in the full IPT-G arm, 3.94 [2.81] in the mini IPT-G arm, and 7.94 [3.71] in the TAU arm). The full IPT-G showed a greater reduction in PHQ-9 scores than TAU within 1 week post intervention (β = −5.79; 95% CI, −7.67 to −3.91; *P* < .001), and mini IPT-G also showed reduction compared with TAU (β = −3.97; 95% CI, −5.83 to −2.10; *P* < .001), with both estimates indicating clinically meaningful decreases in depressive symptoms. The difference between mini IPT-G and full IPT-G was not statistically significant (β = 1.82; 95% CI, −0.04 to 3.69; *P* = .05) (Table 3).

**Table 3.** Treatment Effects in the Intention to Treat Population

Outcome	Study arm comparison^a^					
	Full-IPT-G vs TAU		Mini-IPT-G vs TAU		Mini-IPT-G vs full-IPT-G	
	β (95% CI)	*P* value	β (95% CI)	*P* value	β (95% CI)	*P* value
**Post intervention**						
Primary						
PHQ-9 (depression)	−5.79 (−7.67 to −3.91)	<.001	−3.97 (−5.83 to −2.10)	<.001	1.82 (−0.04 to 3.69)	.05
Functioning	0.20 (−0.10 to 0.50)	.20	0.02 (−0.28 to 0.32)	.88	−0.17 (−0.47 to 0.13)	.25
Key secondary						
NSESSS-PTSD adult	2.64 (−1.48 to 6.76)	.21	5.04 (0.95 to 9.13)	.02	2.40 (−1.63 to 6.43)	.24
HITS (intimate partner violence)	1.10 (−0.35 to 2.55)	.14	0.74 (−0.70 to 2.18)	.32	−0.36 (−1.79 to 1.07)	.62
CORE-10	−3.31 (−6.17 to −0.45)	.02	−1.43 (−4.27 to 1.41)	.32	1.88 (−0.92 to 4.67)	.19
Brief COPE Inventory	−0.07 (−2.06 to 1.93)	.95	−1.04 (−3.02 to 0.94)	.31	−0.97 (−2.92 to 0.98)	.33
General Self-Efficacy Scale	0.87 (−2.49 to 4.23)	.61	0.50 (−2.83 to 3.83)	.77	−0.37 (−3.65 to 2.90)	.82
MSPSS	−0.08 (−0.62 to 0.46)	.76	−0.27 (−0.80 to 0.27)	.33	−0.18 (−0.71 to 0.35)	.50
WHODAS 2.0 (Short Version)	0.17 (−6.92 to 7.25)	.96	3.10 (−3.94 to 10.13)	.39	2.93 (−4.00 to 9.86)	.41
WHO-5 Well-Being Index	11.05 (−0.74 to 22.84)	.07	4.04 (−7.66 to 15.75)	.50	−7.01 (−18.53 to 4.52)	.23
**Follow-up **						
Primary						
PHQ-9 (depression)	−2.22 (−4.25 to −0.18)	.03	−1.38 (−3.25 to 0.48)	.15	0.83 (−1.25 to 2.92)	.43
Functional limitations	−0.06 (−0.38 to 0.27)	.73	−0.04 (−0.34 to 0.26)	.79	0.02 (−0.31 to 0.34)	.93
Key secondary						
NSESSS-PTSD adult	0.84 (−3.62 to 5.30)	.71	0.49 (−3.60 to 4.58)	.82	−0.35 (−4.84 to 4.14)	.88
HITS (intimate partner violence)	1.33 (−0.23 to 2.88)	.09	0.74 (−0.70 to 2.18)	.31	−0.59 (−2.15 to 0.98)	.46
CORE-10	−0.28 (−3.37 to 2.82)	.86	−0.80 (−3.63 to 2.04)	.58	−0.52 (−3.63 to 2.60)	.74
Brief COPE Inventory	−0.01 (−2.17 to 2.15)	>.99	1.37 (−0.61 to 3.35)	.18	1.38 (−0.83 to 3.59)	.21
General Self-Efficacy Scale	−1.23 (−4.85 to 2.40)	.51	−0.92 (−4.25 to 2.40)	.59	0.30 (−3.41 to 4.01)	.87
MSPSS	−0.15 (−0.74 to 0.44)	.82	0.21 (−0.33 to 0.76)	.66	0.36 (−0.23 to 0.96)	.46
WHODAS 2.0 (Short Version)	3.66 (−4.01 to 11.33)	.35	−0.72 (−7.75 to 6.31)	.84	−4.38 (−12.10 to 3.34)	.27
WHO-5 Well-Being Index	10.34 (−2.42 to 23.10)	.11	12.48 (0.79 to 24.18)	.04	2.15 (−10.69 to 14.99)	.74

Abbreviations: COPE, Coping Orientation Problems Experiences; CORE-10, 10-item Clinical Outcomes in Routine Evaluation; HITS, Hurt, Insult, Threaten, Scream; IPT-G, group interpersonal psychotherapy; MSPSS, Multidimensional Scale of Perceived Social Support; NSESSS-PTSD, Severity of Posttraumatic Stress Symptoms–Adult National Stressful Events Survey PTSD Short Scale; PHQ-9, 9-item Patient Health Questionnaire; TAU, treatment as usual; WHO-5 Well Being Index, World Health Organization 5-item Well Being Index; WHODAS 2.0, World Health Organization Disability Assessment Schedule 2.0.

^a^ β coefficients represent mean differences between arms from linear mixed-effects models with conditional likelihood estimation to adjusted for repeated measures within participants. Models included time to intervention arm to and time × arm interaction. Two-sided α = .05 indicated statistical significance.

At 6 months, treatment effects were attenuated. The reduction in PHQ-9 scores for full IPT-G compared with TAU was smaller (β = −2.22; 95% CI, −4.25 to −0.18; *P* = .03), and reduction for mini IPT-G compared with TAU was not statistically significant (β = −1.38; 95% CI, −3.25 to 0.48; *P* = .15). The difference between IPT arms remained nonsignificant (β = 0.83; 95% CI, −1.25 to 2.92; *P* = .43). No between-group differences were observed for functional limitations within 1 week post intervention (full IPT-G vs TAU: β = 0.20 [95% CI, −0.10 to 0.50; *P* = .20]; mini IPT-G vs TAU: β = 0.02 [95% CI, −0.28 to 0.32; *P* = .88]) or at 6-month follow-up (full IPT-G vs TAU: β = −0.06 [95% CI, −0.38 to 0.27; *P* = .73]; mini IPT-G vs TAU: β = −0.040 [95% CI, −0.34 to 0.26; *P* = .79]) (Table 3). Secondary outcomes and subgroup analysis are given in eMethods 11 and 12, eTable 6, and eFigures 11 and 12 in Supplement 2).

## Discussion

In this pilot trial, we evaluated feasibility and preliminary effectiveness of 2 versions of IPT-G, a full version and a mini version, both delivered by CHPs, compared with TAU. The findings offer compelling evidence for feasibility of task-shifting IPT delivered in primary care and generate critical insights into the relationship among intervention dosage, adherence, and clinical outcomes.

### Feasibility, Adherence, and Mini IPT-G Advantage

Our paramount finding is the superior feasibility and adherence profile of the mini-IPT-G protocol. A significantly higher treatment completion rate (77.8% for mini IPT-G vs 52.8% for full IPT-G) and consistently better session-by-session attendance demonstrate that the shorter protocol was more acceptable and sustainable for participants. This is further supported by the higher follow-up retention rates in the mini IPT-G arm at both the 1-week (92.1%) and 6-month (86.8%) postintervention assessments. Declining attendance in full IPT-G suggests participant burden and logistical challenges associated with a longer commitment, a common barrier in psychological treatments in LMICs.^38,39^ Furthermore, the slightly higher fidelity ratings for mini IPT-G (98.5% vs 92.0%) suggest that a condensed protocol may be easier for CHPs to deliver with consistency, enhancing implementation quality. This collective evidence positions mini IPT-G as a more scalable and potentially sustainable model in resource-constrained contexts.^39,40,41^

### Clinical Efficacy: Trade-Off Between Dosage and Accessibility

On the primary outcome of depression, both IPT interventions demonstrated large, statistically significant, and clinically meaningful reductions in PHQ-9 scores post intervention compared with TAU, with very large within-group effect sizes (Cohen *d* > 1.8). Session-by-session data indicated that most dramatic improvement occurred early in treatment for both groups, aligning with the concept of a front-loaded therapeutic effect. While the point estimate and between-group effect size favored full IPT-G post intervention, the mixed-effects model indicated that mini IPT-G was not statistically inferior to full IPT-G. This is a crucial result: it suggests that the mini IPT-G protocol captured a substantial portion of the clinical benefit achieved by the full IPT-G.

A key finding of this study is the comparison between the mini IPT-G and full IPT-G protocols. Our data reveal that substantial improvement occurred within the first 4 sessions for both arms, with mini IPT-G capturing this initial, rapid progress. Therefore, we conclude that a dose of 4 sessions is optimal for perinatal adolescents. While full IPT-G showed a continued, gradual decline in symptoms, the magnitude of additional benefit is valuable to those with higher clinical needs or social support, as it includes doubled treatment duration, albeit at a risk of lower completion rate. As our participants improved, they returned to school or their livelihood, indicating the compelling need for shorter treatments. This study provides strong evidence that the mini IPT-G protocol is sufficient as a first-line treatment, offering a favorable balance of efficacy and efficiency. It positions mini IPT-G as a highly scalable intervention that can increase access to evidence-based psychological care without a substantial loss of effectiveness.

Attenuation of between-group effects at the 6-month follow-up, where neither active arm was statistically superior to TAU, warrants careful interpretation. This finding indicates a need for booster sessions for the mini IPT-G, depending on participants’ needs, or ongoing support to maintain initial gains. However, the maintained, substantial within-group effect sizes for both IPT-G arms at follow-up suggest that symptomatic relief was meaningful and persistent for most participants, even if the statistical advantage over a TAU group that also showed some improvement was not sustained. These findings suggest intervention effects may vary by baseline depression severity. Full IPT-G demonstrated consistent benefits across both severity groups, with particularly strong short-term effects among participants with moderately severe symptoms. Mini IPT-G demonstrated more limited and less consistent effects, particularly among those with milder symptoms, where outcomes were comparable to those of TAU. While these exploratory analyses were not powered for definitive subgroup comparisons, they provide preliminary support for tailoring intervention intensity according to baseline symptom severity.

### Specificity of Effects and Secondary Outcomes

Findings on secondary outcomes highlight specific mechanisms through which IPT-G may operate. The significant reduction in psychopathology symptoms (ie, the 10-item Clinical Outcomes in Routine Evaluation measure) for full IPT-G and in posttraumatic stress disorder symptoms for mini IPT-G points to the transdiagnostic potential of an interpersonal framework. However, the largely null findings for other outcomes, such as self-efficacy, social support, and functioning, in which IPT is embedded, suggest that while IPT effectively alleviates core depressive symptoms, it may not directly target or sufficiently alter these broader psychosocial constructs within the given time frame and dosage. This specificity reinforces the construct validity of the interventions while indicating areas where adjunctive or integrated components might be needed. However, it should be added that these measures of self-efficacy, family functioning, or coping have not been validated or culturally adapted for African settings, and future work on IPT implementation in LMICs should focus on refining contextual process and outcome measures.

### Limitations

This study has some limitations. As a feasibility study, it was not powered to detect definitive treatment effects; therefore, findings should be interpreted with caution. The relatively small sample size limits statistical power and generalizability to broader adolescent perinatal populations. Postintervention assessments occurred at different points (week 4 for mini-IPT-G and TAU, week 8 for full-IPT-G), reflecting the clinical duration of each intervention, which limits direct comparability of end-of-treatment effects. High attrition, consistent with the rate of approximately 35% reported by Meffert et al,^42^ may have introduced bias and limited the ability to detect differences between groups. Additionally, the study was not designed to explore mechanisms of change, such as dose-response relationships or the influence of cultural adaptations, which are important considerations for optimizing IPT delivery among pregnant adolescents. Future research should address these limitations through larger, adequately powered trials incorporating contextual cultural adaptations and evaluate the scalability of IPT’s benefits in low-resource settings.

## Conclusions

This pilot randomized clinical trial provided strong evidence that the mini IPT-G protocol was highly feasible and effective for reducing depression when delivered by trained CHPs. The critical trade-off observed was between the marginally superior (but not statistically significant) postintervention efficacy of full IPT-G and the decisively superior adherence, retention, and completion rates of mini IPT-G. From a public health and implementation perspective, mini IPT-G presented a compelling “best buy,” offering a large proportion of clinical benefit for a fraction of participant and CHP burden, thereby potentially reaching a wider population in need. However, targeted strategies, including digital screening or telephone follow-ups tested for more vulnerable individuals, could augment longer-term benefits.

## Notes

Supplement 1.
Trial Protocol

Supplement 2. **eMethods 1.** Facilitator Recruitment and Training **eMethods 2.** Sample Size and Power **eMethods 3.** Primary Outcome Analysis **eMethods 4.** Effect Sizes **eMethods 5.** Hypothesis Testing and Significance Level **eMethods 6.** Analysis Population **eMethods 7.** Multiple Comparisons **eMethods 8.** Missing Data Handling **eMethods 9.** Subgroup and Sensitivity Analyses **eMethods 10.** Software and Code Availability **eMethods 11.** Secondary Outcomes **eMethods 12.** Subgroup Analysis by Baseline Depression Severity **eTable 1.** Description of Intervention and Comparison Arms **eTable 2.** Summary of Study Measures, Psychometric Properties, and Scoring **eTable 3.** Baseline Characteristics by Completeness of PHQ-9 Data **eTable 4.** Additional Baseline Characteristics by Intervention Arm **eTable 5.** Attendance Metrics by Intervention Arm **eTable 6.** Adjusted Differences in PHQ-9 Scores by Intervention Group and Baseline Severity **eFigure 1.** Session by Session Attendance Rates **eFigure 2.** Mean FFC Over Time by Intervention **eFigure 3.** Mean WHODAS Over Time by Intervention **eFigure 4.** Mean WHO Well-Being Over Time by Intervention **eFigure 5.** Mean PTSD Over Time by Intervention **eFigure 6.** Mean Multidimensional Scale of Social Support Over Time by Intervention **eFigure 7.** Mean HITS Over Time by Intervention **eFigure 8.** Mean General Self-Efficacy Over Time by Intervention **eFigure 9.** Mean CORE-10 Over Time by Intervention **eFigure 10.** Mean COPE Over Time by Intervention **eFigure 11.** Average Depression Scores by Session and Intervention Arm **eFigure 12.** Average Depression Scores by Session and Individual
eReferences.

Supplement 3.
Data Sharing Statement
