# Supplementary material for: Group Interpersonal Psychotherapy for Depression in Perinatal Adolescents in Kenya: A Pilot Randomized Clinical Trial
Source: JAMA Netw Open. 2026 Jun 16;9(6):e2618255. doi: 10.1001/jamanetworkopen.2026.18255 (PMC13273492; doi:10.1001/jamanetworkopen.2026.18255)
Supplement: Supplement 1. — Trial Protocol [file jamanetwopen-e2618255-s001.pdf]

IMPLEMENTING MENTAL HEALTH INTERVENTIONS FOR PREGNANT ADOLESCENTS IN PRIMARY CARE LMIC CONTEXTS

Protocol Version

V4.1

January24, 2022

Declaration

Funding Agency: The PI would like to acknowledge Fogarty International Centre and NIH for the K43 Emerging Global Leader award (award number TW010716-01A1).

Declaration of Interest

We declare that this proposal is my original work and has not been presented for the award of any degree at any other University.

PrincipleInvestigator

Dr. Manasi Kumar, Senior Lecturer, Department of Psychiatry
University ofNairobi

Signature Date: 24th January, 2022

Mentors

Prof Caleb Othieno, Department of Psychiatry, University of Nairobi, Kenya

Signature Date: 24th January, 2022

Prof Mary McKay, Professor and Dean, Brown School of Social Work, Washington University at St. Louis, M

Signature Date: 24th January, 2022

Prof Keng Yen Huang, Associate Professor, Child and Adolescent Psychiatry, NYU

Signature________________________. Date: 24th January,2022

Field and research Assistant

Vincent Nyongesa

Operational Definitions

Post-partumdepressionwhichisalsocalled**postnataldepression**isatypeofclinicaldepression which can affect women, typically after child birth. It is one of the common mental disorders among mothers after child birth. The symptoms include low mood, tiredness, and insomnia, lack of energy, forgetfulness, irritability and poorfunctioning.

Perinatal depression is typically defined as a non-psychotic depressive episode of mild to major severity that occurs during pregnancy or post-delivery.

Adolescence is a transitional phase of growth and development between childhood and adult-hood. This age range falls within World Health Organization (WHO) definition of young people, which refers to individuals between ages 10 and 24. Furthermore, during adolescence, the individual experiences an upsurge of sexual feelings following the latent sexuality of childhood. It is during adolescence that the individual learns to control and direct sexual urges.

Groupinterpersonalpsychotherapyisatherapeuticinterventionadministeredinagroupof6-8 participants meant to improve attachment mechanisms involving emotional understanding, social support and learning to cope with current life. It focuses on the important interpersonal changes and challenges women experience during the postpartum period and uses 6-16 sessions utilizing differentmodels.

Dissemination and Implementation Science describes implementation science as the methods to promote the systematic uptake of clinical research findings and other evidence-based practices into routine practice and hence improve the quality and effectiveness of health care. Dissemination is defined as the targeted distribution of information and intervention materials to a specific public health or clinical practice audience. The intent is to spread knowledge and the associated evidence-based interventions.

WHO mental health plan runs from 2013-2020 and encompasses four major objectives that are: strengthening effective leadership and governance for mental health, providing comprehensive, integrated and responsive mental health and social care services in community-based settings, implementing strategies for promotion and prevention in mental health and strengthening information systems, evidence and research for mental health.

WHO mhGAP The WHO Mental Health Gap Action Programme (mhGAP) aims at scaling up services for mental, neurological and substance use disorders for countries especially with low- and middle-income and provides clinical guidance on supporting objectives laid out in the WHO mental health plan.

List of Abbreviations and Acronyms

WHO – World Health Organization

CFIR – Consolidated Framework for Implementation Research
D & I – Dissemination and Implementation
G-IPT/IPT-G – Group Interpersonal Psychotherapy
MH – Mental Health
SDGs – Sustainable Development Goals
LMICs – Lower- and-Middle IncomeCountries
KDHS – Kenya Demographic Health Survey
KNBS – Kenya National Bureau ofStatistics
mhGAP-IG - Mental Health Gap Action Program- Intervention Guide
EBI - Evidence Based Intervention
SMART - Strengthening mental health research training in Africa
MOH – Ministry of Health
MOE – Ministry of Education

UNFPA – United Nations Population Fund
MCH – Maternal and Child Health
FGD – Focused Group Interviews
KII – Key Informant Interviews
NIMH – National Institute of Mental Health, US

Synopsis

Objectives

High prevalence of adolescent pregnancies in low-and-middle-income countries (LMICs) has resulted in a tremendous public health burden. Many adolescent pregnancies are unwanted, unplanned, and outside of marriage. When adolescent girls become mothers, their opportunities for economic and educational growth are limited. A burgeoning number of adolescent mothers from LMICs are also marginalized due to poverty or gender-based traditions, which further limit their access to health resources, social protection/ support and personal development. Structural inequalities and adverse circumstances have resulted in high prevalence of depression in adolescent mothers. Despite the enormous mental health needs for adolescent mothers in LMICs, child and adolescent mental health service remains in its' infancy. There is a need to advance cultural and context-sensitive perinatal depression interventions that can align with adolescents' living contexts and developmental needs and simultaneously address multiple resource challenges in provision of mental health care in LMIC settings. This application seeks to respond to these adolescent perinatal depression intervention and implementation research gaps by i) utilizing WHO Mental Health Treatment Gap Action Program (mhGAP) and Group Interpersonal psychotherapy (IPT-G) depression care packages that has shown to be efficacious for adult depression, and further test the approaches in adolescents with perinatal depression in Kenyan maternal and child health (MCH) servicesettings.

The **specific aims**of this study are to:

1. Explore barriers and strategies to improve mhGAP-IG/IPT-G fit and implementation for adolescent perinatal depression in MCH clinics inKenya;
2. Develop adapted version of IPT-G/ and mhGAP-IG, and build MCH capacity for implementation;
3. Test feasibility and implementation-effectiveness outcomes for the adapted version of mhGAP-IG/IPT-G.

Study Design

To address Aim 1, we will recruit stakeholders representing diverse perspectives and focus group discussions. For Aim 2, to build capacity in health research and facility staff for adolescent depression care, we will train 20 researchers and 16 providers in modified mhGAP perinatal depression care and in modified IPT-G using task-sharing and collaborative care strategies. **We will integrate testing of MMAP tools with 250 adolescents ages 10-19 and 50 pregnant adolescents from the two sites. We will carry out mixed methods work involving 8 FGDs and 20 KIIs to validate the MMAP tools. We will also carry out HAT adaptation to prepare an enhanced treatment as usual arm materials. As the COVID supplement will be worked on during this time, we would prepare an mhGAP/IPT-G online training for HCWs.** For Aim 3, we will recruit 90 pregnant adolescents from MCHs to participate in a three-arm intervention implementation evaluation study: a) IPT-G full version (8 sessions) b) IPT-G mini (4 sessions) and c) wait-list control. To evaluate implementation-effectiveness of the IPT-G/mhGAP, we will apply a hybrid effectiveness-implementation design and randomized control trial (RCT). The evaluation constructs will be guided by the Proctor's Conceptual model of Implementation Research (Proctor, et al., 2011), which consider implementation, service, and patient outcome indicators. Interviews with partners and caregivers of adolescents, adolescents and health care providers will be conducted before and after intervention. Primary outcomes will be focus on acceptability, engagement with the interventions, and effectiveness on adolescents' depression.

Public Health Significance: This research addresses significant global public health problem and critical child mental health implementation research and service gaps in LMICs. It aligns closely with the NIMH strategic plan, Kenya Vision 2030, UN SDGs, and WHO mhGAP framework. Results of this proposal will advance implementation research and yield need knowledge and tools for addressing similar global health gaps in other LMICs.

Contents

a. Principal Investigator 3
Declaration 19
Operational Definitions 21
List of Abbreviations and Acronyms 22
Synopsis 23
1. Background 29
1.1 Background 29
1.2 Literature Review 30
1.2.1 Adolescent Pregnancies and Unmet Adolescent Needs in Kenya. 30
1.2.2 Global Agenda and Strategies for Addressing Depression and Mental Health Needs in LMIC Settings. 30
1.3 Problem statement 31
1.4 Study significance 32
2. Study Purpose and Objectives. 32
2.1 Purpose 32
2.2 Aims and Objectives 33
Aim 1 (1-18months). 33
3. Study Design 35
3.1 Overview 35
3.2 Study Framework 37
3.3 Methodology for Aim1 38
3.4 Methodology for Aim2 48
3.5 Methodology for Aim3 54
3.6 Locations/Facilities. 59
3.7 Study Participants 59
4. Ethical Considerations 60
4.1 Recruitment and enrolment: 60
4.2 Potential risks 61
4.3 Adequacy of protection of human subjects 62
4.4 Potential benefits of proposed research to human subjects and others. 62
4.5 Data Management 63
Budget 66
References 70
Appendices 57
Appendix 1: Adolescent Informed Consent and Assent 57
Appendix 2: Adolescent Informed Consent and Assent (Swahili translation) Kibali na Maarifa ya Vijana . 60
Appendix 3. Parents/Guardians/Male Partner Informed Consent 64
Appendix 4. Parents/Guardians/Male Partner Informed Consent (Swahili Translation) 66
Appendix 5. Informed Consent for Health Care Worker/Community Health Workers Baseline Survey 68

Appendix 6. Informed Consent For Health Care Worker/Community Health Workers Baseline Survey (Swahili Translation) 71
Appendix 7. Informed Consent For Workshop And Stakeholder Participation 74
Appendix 8. Informed Consent For Workshop And Stakeholder (Swahili Translation)..... 77
Appendix 9: Consent Form for Research Assistants 80
Appendix 10: Consent Form for Research Assistants (Swahili Translation) 81
Appendix 11: Consent Form for Health Centre Management Team And Advisory Board 82
Appendix 12: Consent form for Health centre Management team and Advisory Board (Swahili translation) 83
Appendix 13: Consent Form For Technical Advisory Board 84
Appendix 14: Consent form for Technical Advisory Board (Swahili translation)..... 85
Fomu ya kibali kwa timu ya Bodi ya Ushauri wa kitaalam 85
Appendix 15: Consent Form for Community Advisory Board..... 86
Appendix 16: Consent form for Community Advisory Board (Swahili translation) Fomu ya kibali kwa timu ya Bodi ya Ushauri wa Jumuiya 87
Appendix 17: ASSENT FORM for 10-17 years..... 88
Appendix 18: Consent form for students 18-19 years of age..... 90
Appendix 19: CONSENT FROM CAREGIVERS 10-17 years 92
Appendix 20: Informed Consent for Health Care Worker/Community Health Workers-participation in mhGAP and IPT-G etraining content development-Covid Supplement..... 94
Appendix21: Informed Consent and Assent for HAT 97
Appendix 22: Informed Consent For Workshop And Stakeholder Participation in HAT 100
Appendix 23: Participant information sheet (PIS)..... 103
Appendix 24: Adolescent Informed Consent and Assent for trial..... 108
Appendix 25: Verbal consent..... 111
Appendix 26: Fomu ya Kibali kwa miaka 10-17 112
Appendix 27: Fomu ya idhini kwa wanafunzi wa miaka 18-19 114
Appendix 28: IDHINI KUTOKA KWA WALEZI WA MIAKA 10-17..... 116
Appendix 29. Informed Consent for Health Care Worker/Community Health Workers-participation in mhGAP and IPT-G etraining content development-Covid Supplement (Swahili Translation) 118
Appendix 30: Adolescent Informed Consent and Assent (Swahili translation) - HAT 121
Appendix 31. Informed Consent For Workshop And Stakeholder Participation in HAT (Swahili Translation) 124
Appendix 32: Participant Information Sheet (PIS) (Kiswahili) 126
Appendix 33: Adolescent Informed Consent and Assent for trial (Kiswahili) 131
Appendix 34: Verbal consent (Kiswahili) 135
Summary table of tools sequence..... 136
Training tools for mhGAP and IPT 138
Appendix 35: Evidence-Based Practice Attitude Scale..... 138
Appendix 36: (Research & Service Capacity Building to prepare for implementation of Adapted version of IPT-G and Workflow)..... 140

Appendix37: mhGAP training assessment questions for e-training 146
Appendix 38: Multiple Choice Questions Answers 166
Appendix 39: Facilitator Rubric 167
Pilot feasibility and implementation trial outcomes 170
Appendix 40: Study eligibility checklist 170
Appendix41: Socio Demographic Questionnaire 171
Appendix 42: Edinburgh Postnatal Depression Scale (EPDS)Form* 173
Appendix 43: Patient Health Questionnaire- 9 175
Appendix44: Alcohol, Smoking and Substance Involvement Screening Test (ASSIST-Lite) 176
Appendix 45: The CRAFFT Questionnaire (version 2.1) 177
Appendix 46: Adverse Childhood Experiences (ACEs) Questionnaire 178
Appendix 47: Kessler Psychological Distress Scale (K10) 179
Appendix 48: Severity of Posttraumatic Stress Symptoms— 181
Appendix 49: World Health Organization Disability Assessment Schedule 2.0 183
Appendix 50: MMAP Adolescent questionnaire - EXAMPLE – PART 1 of 2 - contains: 185
Appendix 51: MMAP Adolescent questionnaire – EXAMPLE – PART 2 of 2: 192
Appendix 52: Hurt, Insult, Threaten, and Scream (HITS) tool for intimate partner violence screening 196
Appendix 53: Adolescent and Family Effectiveness Outcomes 197
Appendix 54: Core 10 outcome measure 198
Appendix 55: Instructional Skill development 199
Appendix 56: WHO-5 Well-being index 201
Appendix 57: General Self-Efficacy Scale (GSE) 202
Appendix 58: Multidimensional Scale of Perceived Social Support 203
Appendix 59: IPT INTERPERSONAL INVENTORY 204
Appendix 60: Malawi Developmental Assessment Test (MDAT) 212
Appendix 61: EQD 227
Translation Monitoring Form 229
Health care worker outcomes 230
Appendix 62: My View About Group Interpersonal Psychotherapy for peripartum adolescent Project (CHW version) 230
Appendix 63: Implementation Process Experience 231
Appendix 64: IPT rating Scale for non-specialist HCWs and for training IPT-G supervisors 233
Appendix 65: GROUP IPT KNOWLEDGE TEST (Ref: WHO IPT Manual ,2016) 240
Appendix 66: Becci IPT competency assessment 241
Appendix 67: HCW feasibility measure domains 244
Appendix 68: mhGAP Questionnaire outcomes 247
Appendix 69: MCH Service Contexts (for mhGAP & IPT-G) For MCH Staff & Potential Implementers 248
Apendix 70: My Work Environment-This section ask your views about your health facility environment 254
Appendix 71: Mental Health Knowledge/Beliefs 257
Appendix 72: AMHR Dissemination and Implementation Science Measure: Provider Version 260

Appendix 73: The following questions are adapted from the Program Sustainability Assessment Tool (PSAT) 271
Appendix 74: Implementation Partnership Questionnaire 280
Qualitative interview guides 282
Appendix 75: Aim 1 Assessment Tools (For Study Years 1 & 2) 282
MOVE TO mhGAP and Group-Interpersonal Psychotherapy DISCUSSION 285
Appendix 76: Qualitative Interview Questions 287
Appendix 78: Exit interviews 292
Appendix 79: Endline Evaluation from HCWs through FGDs/KIIs 295
Appendix 80: Interview guide- 297
Appendix 81: Interview/ Focus Group Questions-COVID Supplement 300
Appendix 82: HAT interview guides 305
Training tools for mhGAP and IPT (Swahili translations) 309
Appendix 83: Facilitator rubric (Swahili) 309
Pilot feasibility and implementation trial outcomes (Swahili translations) 312
Appendix 84: Pata orodha ya uhakiki 312
Appendix 85: Socio Demographic Questionnaire (Swahili version) 313
Appendix 86: Edinburgh Postnatal Depression Scale (EPDS) Form* (Swahili translation) 315
Appendix 87: Patient Health Questionnaire – 9 (Swahili version) 317
Appendix 88: Alcohol, Smoking and Substance Involvement Screening Test (ASSIST-Lite)- Kiswahili version 318
Appendix 89: The CRAFFT Questionnaire (Version 2.1) _Kiswahili 320
Appendix 90: Adverse Childhood Experiences (ACEs) Questionnaire (Kiswahili) 321
Appendix 91: Kipimo cha mafadhaiko ya kisaikolojia cha Kessler (K10) 322
Appendix 92: Severity of Posttraumatic Stress Symptoms—Adult National Stressful Events Survey PTSD Short Scale (NSESSS) (Swahili translation) 324
Appendix 93: WORLD HEALTH ORGANIZATION DISABILITY ASSESSMENT SCHEDULE 2.0 (Kiswahili) 326
Appendix 94: DODOSO- MFANO –SEHEMU YA 1 Kwa 2 - ina: 328
Appendix 95: DODOSO–MFANO – SEHEMU YA 2 Kwa 2: 336
Appendix 96: Hurt, Insult, Threaten, and Scream (HITS) tool for intimate partner violence screening (Swahili) 340
Appendix 97: Matokeo ya Ufanisi wa Vijana na Familia 341
Appendix 98: CORE 10 Outcome measure (Kiswahili version) 342
Appendix 100: WHO-5 Well-being index (Kiswahili) 345
Appendix 101: The General Self-Efficacy Scale (GSF) (Swahiliversion) 346
Appendix 102: Multidimensional Scale of Perceived Social Support 347
Appendix 103: IPT Interpersonal inventory (Kiswahili) 348
Appendix 104: EQ-5D (Swahili) 351
Appendix 105: Uzoefu wa Mchakato wa Utekelezaji 353
Appendix 106: IPT rating scale (Kiswahili) 356

Appendix 107: Becci IPT competency assessment (Kiswahili) 363
Appendix 108: HCW feasibility measure domains (Kiswahili) 366
Appendix 109: mhGAP Questionnaire outcomes (Kiswahili) 369
Appendix 110: Mazingira Yangu ya Kazi-Sehemu hii inauliza maoni yako kuhusu mazingira ya kituo chako cha afya 370
Appendix 111: (AMHR) Sayansi ya Usambazaji na Utekelezaji wa Sayansi Hatua: 372
Appendix 112: Implementation Partnership Questionnaire (Kiswahili) 386
Qualitative interview guides 388
Appendix 113: Interview Questions (Swahili translations) 388
Appendix 115: Endline evaluation (Kiswahili) 393
Appendix 116: *Miongozo ya Majadiliano ya Kikundi cha FGD* 395
Appendix 117: COVID Supplement (Kiswahili) 398
Appendix 118: Miongozo ya mahojiano ya HAT 403
Appendix 118: PROGRESS REPORT .... **Error! Bookmark not defined.**

1. Background

1.1 Background

Globally, depression is the leading cause of disease burden in women of reproductive age. Maternaldepressionoccursinwomenduringbothantenatalandpostnatalperiods.Theprevalence of depression is high among pregnant women, with worldwide estimates of 11-18% (2, 3) and is estimatedtobebetween15%-28%inLMIC(4).StudiesinKenyaandneighboringcountrieshave found that younger women (18-24 years) experience greater psychological vulnerabilities (5) and depression in this group ranges from 8.3% to 39% (6, 7, 8) with high risk populations like adolescent mothers, gender based violence affected women reporting higher prevalence estimates of over 40% (9).Young women in Sub-Saharan Africa (SSA) lose around 10.4 disability adjusted life years due to depression during pregnancy. Women who experience antepartum depression oftencontinuetoexperiencedepressivesymptomsintothepostpartumperiod,withmorethan54% of those with postpartum depression reporting depressive episodes before or during pregnancy (10).Pregnantwomenbetweenages18-21yearsareatparticularlyhighriskfordepressioninSSA countries like Kenya because circumstances that lead to their pregnancy often include low socioeconomic status and food insecurity (11), with subsequent increases in unprotected sex with older men in an effort to recruit financial support through sexual partners. These factors are associated with increased risk of Adolescent mothers, gender-based violence, low educational attainment and social stigma, all of which are associated with higher prevalence of depression (9, 8, 12, 13, 14, 15, 16, 17). These vulnerable young women need heath care and psychosocial support in building resilience against challenging barriers and riskfactors.

Consistent with the World Health Organization (WHO) recommendations for Low-and-Middle- Income Countries (LMICs), we propose to use Interpersonal Psychotherapy (IPT), an evidence- based depression treatment with demonstrated efficacy in East African regions of Kenya and Ugandafordepressionduringpregnancy(18,19).IPThasbeenshowntohavehighefficacywhen delivered by a readily available work force of local, non-specialist personnel in East Africa (18, 20, 21, 22, 23). Based on recent studies by Meffert and colleagues in Kenya (19, 24) we believe that IPT’s focus on social support, rebuilding interpersonal relationships by bolstering interpersonal communications skills, focusing on life-transitions make it a promising fit with the mental health care needs of pregnant young women in Kenya. However, young pregnant women in Kenya experience a particular set of stressors which we believe will require adaptation of standard IPT in order to match patient needs. For example, unwed mothers in Kenya are often unable to complete education as planned with related short-comings in earning potential and are susceptibleto exploitation andabusebytheirbiologicalfamiliesandcommunityingeneral.While IPT is well-equipped to address these issues, adaptations are required to address specific role transitions and losses faced by young pregnant women inKenya.

1.2 LiteratureReview

1.2.1 Adolescent Pregnancies and Unmet Adolescent Needs in Kenya.

Adolescents make up nearly 16% of the world's population, and the proportion is greater in Sub-Saharan Africa (23% of the regional population) (25) Over 16 million adolescent girls worldwide give birth between ages 15–19, and the vast majority of these births (95%) occur in LMICs (26). When adolescent girls become mothers, their opportunities for economic and educational growth are further compromised. A burgeoning number of adolescent mothers from LMICs are also marginalized due to poverty, gender-based traditions or geographical resource scarcity which further limit their access to health resources, information, and social protection and personal development (27, 28, 29, 30, 31, 32) Structural inequalities and adverse circumstances have detrimental impact on adolescent mothers' mental health, and set the stage for other adverse medicalconditions,riskybehaviorsandmentalillnessinadulthood(29,33)Estimatedprevalence of depression during perinatal period is 11–18% worldwide, and 30–50% in LMICs (27, 34, 35, 36). The risk for perinatal depression in adolescents is 2–9 times higher than adults (34, 36, 37). High prevalence of adolescent pregnancies and perinatal depression in vulnerable adolescents in Kenya has resulted in tremendous public health burden as well as social and economic burdenfor families and communities (27). Despite the enormous mental health needs for adolescentmothers inLMICs, most perinatal mental health research has been focused on adults and has not considered the unique challenges that adolescent mothers are facing. Most research has also not invested in public health strategies or culturally relevant strategies to empower adolescents and address the service needs of this population in LMICsettings.

1.2.2 Global Agenda and Strategies for Addressing Depression and Mental Health Needs in LMICSettings.

WHO proposed two comprehensive frame works for supporting country efforts to establish and scale-upmentalhealthservices.*TheComprehensiveMentalHealthActionPlan2013-2020(ortheAction Plan)* (38) proposes that in order to improve population mental health in LMICs, system strengthening in 4 areas needs to be considered, including i) developing effective leadership and governance, ii) providing comprehensive, integrated, and responsive mental health services, iii) implementingstrategiesforpromotingmentalhealth,andiv)strengtheningevidenceandresearch formentalhealth.ComplementarytotheActionPlan,*TheMentalHealthGapActionProgramme(mhGAP)*(39,40)focusesonii)andiii)oftheActionPlanandprovidesevidence-basedguidelines and tools to support service system setup, delivery, and provision of evidence-basedinterventions (EBIs) for common mental health disorders in non-specialized health-care settings (41, 42). Specifically, *mhGAP* recommends applications of collaborative, task-shifting and task-sharing implementation strategies (i.e., considering collaboration between mental health specialists and non-specialist health-care providers and redistribution of clinical tasks from mental health specialists to non-specialized providers) in provision of mental health services. The *mhGAP Intervention Guideline (mhGAP-IG*) also provides assessment, management clinical decision- making action flowcharts, and recommended EBIs for treating 8 priority mental healthconditions (includinginterventionsfordepression).AlthoughbothActionPlanand*mhGAP*havebeenapplied

Successfullytoaddressadultdepressionandothermentaldisordersinseveral LMICs(43,44,45), **applicationofthesefrMeworksinchildandadolescentmentalhealthserviceremains in its infancy**. A handful of depression intervention studies in LMICs that utilize EBIs recommended in the *mhGAP* (e.g., Group interpersonal psychotherapy/**IPT-G**, Thinking Healthy Program, Problem Management Plus) have exclusively either focused on perinatal depression forawiderangeofreproductive-agedwomen(29,46,47,48,49,50,51)orinterventionsfordepressedadolescentsinnon-medicalhealthservicesettings(52,53).**Moststudieshavefocusedonefficacy** of EBIs **and have not examined implementation related questions or tested feasibility of *mhGAP-IG***forperipartumadolescentmentalhealth.Morepragmaticresearchthatsimultaneously considers effective mental health leadership development, workforce development strategies,EBI implementation strategies, and effectiveness outcomes is sorelyneeded.

1.3 Problemstatement

While the Action Plan and *mhGAP* have been applied to adult depression, **these evidence-based guidelines have not been applied to adolescent girls with perinatal depression in Kenya**. Therefore, research is needed to apply WHO Recommended Frameworks and EBIs in Kenyan Context for Adolescent Perinatal Depression.

In considering utilizing WHO recommended models in Kenya, several other system and implementation gaps and population characteristics need to be considered. Like many other LMICs, Kenya has weak child and adolescent mental health system with limited mental health professionals and workers (53). In tandem with the global agenda, the Kenya Vision 2030 mental health policy program (developed in 2012) brings in major health system reforms aiming at ensuring equity, people centeredness and participatory approach, efficiency, multi-sectoral approach and social accountability in delivery of mental health services (54), progress in actualizing these changes in child mental health policy and service system development remain considerably slow.

Inadditiontothesystemgaps,thereareother**intervention-implementationresearchgaps**.*First*, in the current *mhGAP-IG*, there are separate evidence-based guidelines and tools for adultdepression (includingperinatal depression) and child and adolescent depression, but interventionsthatintegratebothpregnancyandadolescentneedsforadolescentperinataldepressionarelacking. The adult and child/adolescent versions of *mhGAP*-depression packages focus on somewhat differentcontentsandapproaches.Forexample,inadultdepression,*mhGAP-IG*emphasizesmore on patient’s individual needs and intervention (i.e., psychoeducation, stress reduction and strengthening social support, promotion of improved functioning in daily activities, brief psychological treatment for depression, and pharmacology). For adolescent depression, mhGAP- IG considers critical roles of caregivers, families, and schools/ teachers in depressionintervention givenadolescents’developmentalneeds;therefore,itrecommendsthatdepressioninterventionfor adolescents must consider both individual and family/community related intervention (i.e., consideration of psychoeducation for the child/adolescent and family, guidance on promoting

wellbeing, caregiver support, stressors management, community resources linkage,andcollaboration with teachers/schools in managing child and adolescent depression). Although both adult and child/adolescent depression intervention guidelines/tool packages are relevant to adolescent perinatal depression, how these packages should be adapted to effectively address Kenyanperinataladolescentneedsisyettobesystematicallystudied.Anothergapisthedemand-resource gap, as maternal and child health (MCH) clinics usually serve high volume of pregnant adolescentsoryoungmothers(e.g.,20-30pregnantadolescentsareseenperweek[or960-1440per year] in each MCH clinic in Nairobi, Kenya). Considering high prevalence of adolescent pregnancy, high risk for depression in this population, and low resource in MCH clinics, utilizing WHO recommended depression EBIs (e.g., IPT-G), which usually require more than 5 intervention sessions, may not be practical in meeting population service demands and needs. Additional cost-effective strategies (such as considering lower dose/ intensity/fewer intervention sessions, using group instead of individual approach for intervention, applying low-cost patient/family/community empowerment approaches of service provision) will beneeded.

In summary, given developmental needs and living context differences (e.g., parent-child co-living, connection with schools and health centers) between adults and adolescents, more implementation- effectiveness research for perinatal depression in adolescent population is needed. In addition, given the utility, comprehensiveness, and potential impacts of mhGAP, systematically studying the feasibility of mhGAP for adolescents with perinatal depression merits further inquiry. These areas of research will provide needed knowledge for intervention to reduce burden of perinatal depression for adolescents in Kenya and LMIC in general.

1.4 Studysignificance

This study addresses intervention research and public health gaps for perinatal depression in adolescentsbyutilizingWHOActionPlan,mhGAP,andWHOrecommendedIPT-G.Considering system, leadership, research strengthening needs and needs for cost-effective and culturally sensitive depression interventions for pregnant adolescent in Kenya, we will apply a multi-disciplinary/stakeholder partnership approach to develop and test mhGAP-IG depression interventionguideline/toolsandIPT-GtobeimplementedinMCHclinics.Resultsofthisresearch will advance implementation research and yield need knowledge and tools for addressing important global mental healthgaps.

2. Study Purpose andObjectives

2.1 Purpose

The purpose of this study is to study strategies to improve fit of the mhGAP-depression intervention model (including IPT-G) for pregnant adolescents in Kenya; to develop a Kenyan version of mhGAP/IPT-G intervention program and manual; and to effectively integrate task-shifting/ sharing and collaborative strategies with MCH clinical workflow to implement mhGAP depression intervention for pregnant adolescents in MCH settings.

TheadolescentversionofmhGAP/IPT-GhasnotyetbeendevelopedinKenya.Asafirststep,we will bring a group of leaders/stakeholders from MCHs and communities together to focuson:

(1) Identify strategies for developing an optimized mhGAP/IPT-G for Kenyan pregnant adolescentmothers;
(2) Mapping out implementation/capacity building strategies to fit the integrated model into clinical structure and workflow;and
(3) Carrying out a pilot study to test and estimate impacts of the integrated mental healthcare model in maternal child healthsettings.

2.2 Aims and Objectives

Aim 1 (1-18months).
To explore multi-level implementation barriers, and facilitators to implementation of mhGAP-perinatal depression care guidelines in pregnant adolescents in Kenya. A secondary aim wouldbe to explore the mental health treatment preferences of pregnantadolescents.

1.1 To identify multilevel implementation barriers to the implementation of perinatal depression care in pregnantadolescents
1.2 To identify multilevel implementation facilitators to the implementation of perinatal depression care in pregnantadolescents.
1.3 To understand treatment preferences of pregnant adolescents using conjoint analysis informed by barriers and facilitators (1.1 and1.2)
1.4 To carry out a psychometric evaluation of brief depression, anxiety, suicidality and general functioning tools with general sample of adolescents and pregnant adolescents to develop more sustainable assessments in primary care

Aim 2 (19-30 month). To use Aim 1 findings to develop adapted version of IPT-G/and mhGAP-IG and build MCH capacity for implementation.

2.1 To adapt the mhGAP to Kenyan adolescents and maternal child healthcontexts.
2.2 To adapt the G-IPT to Kenyan adolescents and maternal child healthcontexts.
2.3 To employ a user-centered design to adapt the face-to-face training of mhGAP/IPT-G to an e-version and study the feasibility, acceptability, and usefulness of the e-version of the mhGAP/IPT-G training for health care workers (both health facility staff and community health workers).
2.4 To collaborate with MOH and primary care and community service stakeholders that we have built collaboration relationship with as part of the K to plan and pilot test the e-version of mhGAP/IPT-G training in 2-3 service settings (e.g., KNH, Mathari, andNgara primary health care centre). The research implementation team will provide technical support to service stakeholders/leaders on e-version of mhGAP/IPT-G training and implementation, and to estimate the impacts of the e-training on their clinical service and patient’s mental health outcomes.
2.5 To build maternal child health capacity to implement the adaptedprograms**by** training

HCWs in online training integrating mhGAP/IPT-G through the Fogarty K43 COVID supplement

Aim 3 (31-48 months). To examine feasibility and implementation-effectiveness of the adapted version of integrated mhGAP and IPT-G implementation model (developed from Aim 2) on (a) acceptability (b) engagement (c) preliminary effectiveness in Kenyan adolescents and MCH

3.1 To assess feasibility of the adapted version of mhGAP on Kenyan adolescents and maternal child health contexts.
3.2 Toassessfeasibilityoftheadaptedversionof G-IPTonKenyanadolescentsandmaternal child healthcontexts.
3.3 To assess implementation effectiveness of the adapted versions on three levels (a) acceptability (b) engagement (c) preliminary effectiveness in the management of perinatal depression of pregnant adolescents.

3. StudyDesign

3.1 Overview

The goals of the proposed study are to develop intervention strategies using mhGAP-IG and G-IPT through capacity building and implementation to address depression among pregnant and parenting adolescents.The first 1.5 Years (1-18 months)will focus on mhGAP/IPT-G strategies development to fit Kenyan MCH contexts. The 1.5-2.5 Years (19-30 months) will focus on mhGAP/IPT-G adolescent version manual development, and capacity building for mhGAP/IPT-G implementation and research. The 2.5-4 Years (31-48 months)will focus on implementing mhGAP/IPT-G model in real world MCH settings and evaluate the impacts. Figure 1 shows research tasks, processes, and timeline.

This research study will be conducted based on a partnership approach that includes diverse governmental, clinical, research, and community stakeholders. We will establish an Advisory Boardto facilitate cross-levelpartnershipsandleaders-stakeholders'discussionaroundadolescent mental health intervention, service, and policy issues. This approach also has implications for building leadership and system capacity (e.g., though co-learning and increasing stakeholders' attention and knowledge to the gaps and developing actions toward strategy and policy development(58).

The Advisory Board will include 13 representatives, including 3 MCH (1 leader/director, 1nurse, 1CHW),2governmental(1MOE,1MOH),2academics(1healthservice/implementation,1 Clinicalresearchers),1advocacy(1representativeNGOleader),and2communitystakeholders(2 caregivers of pregnant adolescent, 4 adolescent representatives). The members will meet regularly (3 times a year) throughout studyyears.

The eligibility criterion and terms of reference of the advisory are as follows.

For the four adolescent representatives we will seek one pregnant adolescent and one new adolescent mother and two male adolescents. The eligibility criterion would be:

- Willing to consent toparticipate,
- One pregnant adolescent and one new mother ages13-16,
- Another pregnant adolescent and new mother ages16-18,
- Willing to join the meetings held at the community center or at the healthfacility.

For all other stakeholders, the eligibility wouldbe:

- Willingnesstosigninformedconsenttoparticipateinthestudyandforthedeliberations to be recorded,
- Willing to engage with the issues around mental health and adolescentpregnancy,
- Willingness to commute to the health facility or community center for meetings three times ayear.

The meetings may last between 2-3 hours and a minimum of 9 members need to be present for the meeting to take place.

The advisory board members would be paid transport and offered refreshments like tea, soda etc for the time they spend with the researcher and her team.

The meetings would center on:

1. understanding barriers and strategies to mitigate these barriers to mental health care in pregnantadolescents,
2. findings of the qualitativework
3. Discussing mhGAP adaptationprocess.

Additionally, there would be a technical advisory board. *The technical advisory board comprises ofmemberswhoseorganizationsprovidedsupportlettersduringthegrantandformallycommitted to providing technicalguidance*.

Eligibility:

- Adultsabove18ofyearsandtechnicalpersonsrepresentingmentalhealthandhealthpolicy implementing partners inKenya,
- willing to sign consent form for participation in thestudy,
- Willingness for discussion to berecorded.

The meetings would be held twice a year to discuss the key findings of the work and refine disseminationandimplementationplanfurther.Refinementofthethreeimplementationstrategies – task-sharing/shifting,engagementwithadolescentsandstigmareductionwouldbethekeyfocus. Thetechnicaladvisorywouldbepaidtransportallowanceandofferedrefreshments.Themeetings would last 2-3 hours on days convenient for most members.

- UNFPA, Dr. Ademola Olajide and Lilian Langat- responsible for sexual and reproductive health issues, working on adolescent girl child mentalhealth
- MoH, Simon Njuguna -responsible for integrating mhGAP in clinical workflow at primary carelevel,
- Nairobi County Health Directorate, Raphael Muli, - responsible for task sharing and stigma reduction strategies to addressdepression.
- Nairobi County preventive health- Carol Ngunu
- WHO, Joyce Nato – responsible WHO mhGAP adaptation and local capacity building in mentalhealth
- UNICEF, Regional Office- Fatima Gohar Adolescent Specialist
- WHO HQ Geneva- responsible for G-IPT adaptation and mhGAP for adolescent peripartum depressionmanagement – Drs Chiara Servelli and Neerja Chowdhary

Figure 1. Research Tasks, Processes , and Timeline

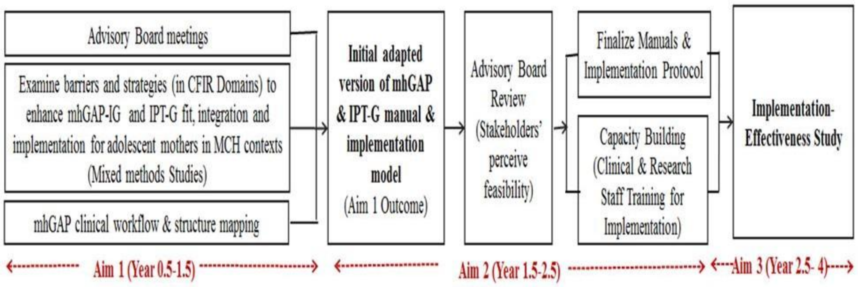

3.2 StudyFramework

The adaptation, implementation, and evaluation of the mhGAP/IPT-G will be **guided by an integratedconceptualmodel**thatcombinedtwoD&Iframeworks.*TheConsolidatedFrameworkfor Implementation Research (CFIR)*(55), which accounts for five domains of contextual factors that may influence key determinants that impact outcomes, will be used to guide Aims 1 & 2 studies. A rigorous mixed methods study will be used to study strategies to adapt and improve fit of mhGAP-IG and IPT-G for Kenyan adolescent population and MCH implementation contexts. *TheConceptualmodelofImplementationOutcomes(56)*whichprovidesconceptualconstructsfor guiding implementation, service, and effectiveness outcome assessments, will be applied toguide the evaluation of implementation-effectiveness study (Aim 3). Figure 2 shows our integrated conceptual model for the proposedstudy.

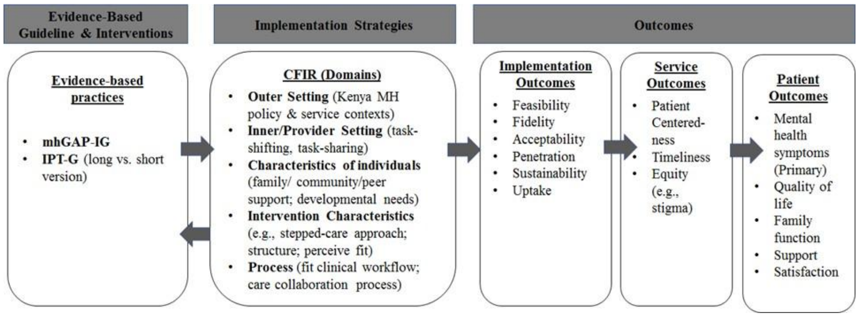

Figure 2. Integrated Conceptual Model for the Study

Figure 3: Timeline

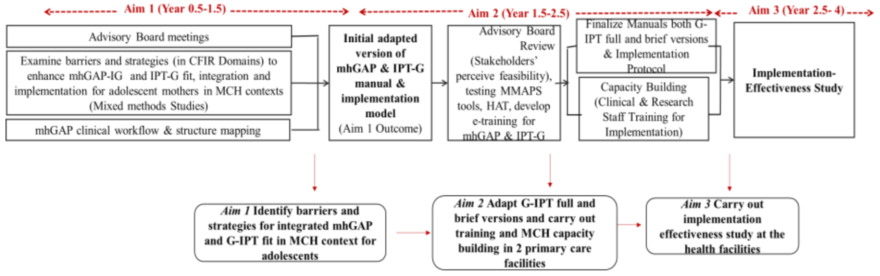

3.3 Methodology for Aim1

(Study barriers and strategies to improve mhGAP-IG/IPT-G fit and implementation).

To understand 'fit' of the mhGAP/IPT-G and study multi-level barriers/strategies to promote utilization and impacts of IPT-G and mhGAP, we will carry out a mixed methods study,includingqualitativeandquantitativedatacollection.Quantitativedatacollectionwillfocus on D&I readiness contexts and new D&I tools pilot testing, which will be used for Aim 3 evaluationstudy.

<u>*Data Collection Focus and Procedures*</u>: We will conduct 8 focus groups (n=40) with representative diverse stakeholders (i.e., including 2groups for pregnant adolescents and new adolescent mothers, 2 groups for adolescent caregivers and partners, 2groups for MCH staff, 1 group for community advocates and policy leaders, and 1 group for child and adolescent mental health professionals). Each focus group discussion (FGD) will have about 5 participants. Each groupwillparticipatein*threeFGDmeetings*.Onemeetingwillfocusonbarriers/strategiesrelated to mhGAP-IG, and the other meeting will focus on barriers/strategies related to IPT-G. The third FGD would be focusing on mental health treatment preferences of pregnant adolescents. Each focus group will last 1½ to 2 hours. Participants will participate in a FGD and complete a survey, andthesewillbecarriedoutinEnglishorKiswahili(asEnglishistheofficiallanguageinKenya). Focus group guides/questions and quantitative survey will be adapted from existing D&I studies (57), or existing D&I measures (see measures under Aim 1). Stakeholders' views on barriers and strategies in <u>5 CFIR domains</u> (see Figure 2 second left box) will be discussed separately for mhGAP and IPT-G. Informed by our previous need assessment work, strategies related to *livelihoodproblem*,*partner/familyengagement*,*knowledgegap*,*andstigmareduction*willalsobe explored.

- For focus group with <u>*adolescents and families/partners*</u>*(n=20, recruited through 2 MCH clinics that we have partnership with from previous MCH epidemiological studies)*, discussion will focus on CFIR individual, intervention, and inner setting domains, and examinetheirknowledgerelatedtoperinataldepression,andviews(includingbarriersandpotent

ial strategies) related to adolescent pregnancy, mhGAP/IPT-G (fit, usefulness, perceived feasibility and effectiveness of IPT-full and mini versions), family member/partner engagement, support, and empowerment strategies, and preference for integrated mental health-MCH services. See appendix.

- *For the MCH providers/staff and mental health professionals (n=15, recruited through 2 MCH clinics [same as the sites for family recruitment] and mental health professional networks),* discussion will focus on the CFIR inner setting, individual/staff, intervention, and process domains. We will examine fit, barriers, and implementation strategies for mhGAP and IPT-G implementation in MCH settings (e.g., collaborators' roles, task-shifting/sharing strategies, workflow logistic/coordination strategies), staff attitude/belief toward EBI practices, perceive effectiveness, and MCH implementation climate. See appendix.
- *For the community advocates and policy stakeholders (n=5, recruited through Ministryof Health and NGO networks)*, discussion will focus on the CFIR intervention, process and outer setting domains, and examine barriers and strategies related to mhGAP and IPT-G. In addition, community awareness/public educational and stigma reduction strategies (outer setting strategies) will be explored to facilitate community support to address adolescent pregnancy and maternal mental health needs, which also has implications for public health impact promotion (58, 59). Seeappendix.

To better identify mhGAP/IPT-G workflow barriers and strategies in MCH contexts, MCH site visits and workflow observation will be arranged for the Community and Technical Advisory Board members (n=18, including researcher). The observation will focus on MCH structure and perinatal service workflow. This will allow the Board and research members to gain better understandingabouttheclinicalsetup,implementationbarriersandstrategies,whichwillfacilitate better and more sustainable workflow plan/strategy development to support mhGAP/IPT-G implementation. To ensure confidentiality, informed consent for all participants will be obtained prior to the data collection. Participating provider personnel and family members will be compensated for their time. Participating mental health professionals and officers of NGO/ Ministry of Education/ Ministry of Health will also becompensated.

Creating an adapted G-IPT model for pregnant adolescents in Kenya requires (1) in-depth qualitativeanalysisofdepressioncareneedsfrommultiplestakeholders;(2)analysisofneedsand incorporation of these needs into a treatment manual for this group. Conjoint Analysis has been usedinmarketingresearch,butithasevolvedasarobustdevisetomeasurehealthcarepreferences of patients. Grounded in economic theory, conjoint analysis assumes that clients make choices among products or services by considering characteristics or attributes—for example, size, color or brand or home or clinic-based care—and making tradeoffs among these attributes to arrive ata finalchoice.Inconjointanalysisusedforhealthcare,clientsarepresentedwithhypothetical

Service scenarios with varying combinations of attributes and are asked to choose among these scenarios. Conjoint analysis (CA) and mixed methods such as Q-methodology, Principle Co-ordinate analyses enable us to estimate relative preference weights and their corresponding trade-offswhenitcomestotreatmentpreferencesandengagementstances(38)Patients'preferencesare particularly salient in depression treatment, because multiple efficacious treatments (for example, combination of antidepressants and psychotherapies) and modalities (for example, group and individual)ordifferenttypesofpsychotherapies(cognitive-behavioralorrelationallikeIPT)`exist. Knowing what these young women would consider beneficial and ways to bolster an evidence based and well-accepted group interpersonal psychotherapy for this age group is the key motivation for this study. Conjoint analysis involves defining of lived experience of depression during pregnancy, preferred intervention for depression, barrier-to-care, stigma in seeking mental health care attributes, attribute levels to the above-mentioned themes will be assigned (treatment delivered at home or clinic, depression care by peer or health worker etc and create hypothetical scenarios. Clinical guidelines like WHO's mhGAP, Kenyan National maternal and child health and mental health policies along with formative qualitative work will guide the process. For the secondary aim of addressing adolescent mental health treatment preferences, the following methodology would beadopted.

Target Population: Pregnant adolescents in Kariobangi and Kangemi informal settlements visitingtheMCH andPumwani Maternity hospital as a referral.Throughinitialcontactwiththesewomenwewillusesnowballingtechniqueto reachotherswhohavepoorercommunitysupportandhealthfacilityinteraction,asubsetofhealth workers from the facility, community health workers, school teachers andcaregivers.

Recruitment: Direct approach of soliciting participation from those in these facilities and associated with the facilities.

Inclusion Criteria:

- Seeking primary care at thecenter;
- ages 13-18years;
- ability to give informedconsent;
- availability for duration ofinterview
- intact cognitivefunctioning

Exclusion Criteria:

Women over 18 years of age, and unable to give informed consent.

Enrollment (N=157) and Study Procedures: Using a simple conjoint experimental task, key preferences will be elicited to address the barriers identified above. Multidimensional scaling and will create a list of closed choice scenarios.

SampleSize: Totalsamplesizeof157 youngwomen will be given a short conjointsurvey.Sample size considerations will be based on the standard formula for sample size estimation for discrete

choice experiment analysis published by Johnson and Orme. In which, the rule of thumb as proposed by Johnson and Orme (60, 61) suggests that the sample size required for the main effects depends on the number of choice tasks (t), the number of alternatives (a), and the number of analysis cells (c) according to the following equation: $N > 500c/(t*a)$, indicating that a minimum sample size of 94 respondents was needed to all two-way interactions. As this rule of thumb is sometimes insufficient, and to allow for sub-group analyses, we will then increase the sample size by 40%, resulting in a targeted sample size of 157 respondents(62).

**Table 1: Examples of Conjoint tasks Post-interviews for Aim 1**	
**Characteristics under for conjoint task**	**Discrete choices**
Treatment format	Group or individual
Treatment duration	1,2 or 3 hours
Incentives	Food, transport both or care items for baby
Treatment setting	MCH facility, home, community center
Treatment support	Male partner, mother, aunt or friend
Infant care information	Yes needed or not needed
Lifeskills needed (assertiveness skills, simple Maths/accounting/home economics)	Yes needed or not needed
Delivery of intervention	MCH nurse, social worker, mental worker
Reading material about depression	Yes or no
Links with livelihood support organizations	Yes or no
Nutritional training for self and baby	Yes or no

***Analysis Plan*.** Qualitative data (from focus groups and observation) collected under this aimwill be transcribed and analyzed using *Atlas.ti*, focusing on barriers/strategies themes relatedto

(i) Improving fit of the *mhGAP*-depression intervention model (including IPT-G) for pregnant adolescents; and (ii) enhancing effective integration and implementation of *mhGAP-IG* into MCH settings. Analysis for qualitative data will follow techniques of narrative analysis (63, 64, 65, 66) and a "constant comparison" analytic approach(67, 68) and be carried out by graduate-level students. We will employ content/thematic analysis to assimilate the rich qualitative data. Quantitative data (e.g., knowledge,EBI attitude) will be analyzed using descriptive and multi-level regression analysis. Reliabilityandvalidityofthesurveymeasureswillbeexamined,whichmayberefined andthenbeusedinAim3.Wewillcompileasummaryofthemes/findingsandthenbe presented back to the Advisory Board members forfeedback.

Data Collection and Analysis for the conjoint task: Brief descriptions of treatment andbarrier-stigma-reduction strategies will be read to the participants from a standardized script before the survey is administered. For each pair of scenarios, the participants will be asked to select the one they prefer. Patient characteristics will be examined using standard descriptive statistics.

Multinomial regression will follow binary logit estimates of treatment and barrier-reduction preference parameters through regression analyses. For each regression, the dependent variable will be a binary indicator of treatment acceptance, and independent variables will the attribute levels and future scenarios. The estimated β coefficients indicate the relative utility ofeachattribute level, with positive values indicating stronger preference. SAWSOOTH (69) software willbeusedtoanalyzetheconjointexperimentandSPSS(70)willbeusedfordescriptivestatistics and triangulating conjoint findings to different participantattributes.

The findings would be integrated in the Aim 2 adaptation process.

To carry out a psychometric evaluation of brief depression, anxiety, suicidality and general functioning tools with general sample of adolescents and pregnant adolescents to develop more sustainable assessments in primary care

We intend to add a short exercise of pilot testing of a set of brief tools that have been recommended for MICS data collection on mental health indicators for adolescents in LMICs. These tools are briefer, simpler and currently being tested with adolescents in Belize and South Africa. These tools cover depression, anxiety, suicidality and functional impairment using Revised Children's Anxiety and Depression Scale (RCADS) (71)

We will test these tools on ages 10-14 and 15-19 years in our study sites. The tools which were listed as part of the intervention have been developed for adults though tested on adolescents.

The overall aim of this exercise are to establish the validity and reliability of measures of adolescent mental health, using multiple qualitative and quantitative approaches, including focus groups, translation/back translation, clinical evaluation, and psychometric analyses.

The specific objectives are

1a) Examine the content validity of tool items within each of the tools for the Kenyan context, using focus group discussions and interviews, and appropriateness of items using cognitive interviewing techniques.

1b) Validate the key measures of mental health for adolescents for a questionnaire module against a gold standard diagnostic interview and classical and modern psychometric analyses.

2: Explore mental illness stigma, help seeking behavior, perspectives on access to social support, mental health care or social services utilization, and knowledge about mental health, using focus groups.

3: Understand younger and older adolescents' conceptions of mental health and wellbeing and how proposed intervention materials can be adapted to best suit Kenyan adolescents, and how materials could be adapted for pregnant and parenting adolescent populations in various LMIC settings.

Table 1b: questionnaire testing phases, steps and relevant activities

Phase	Step	Activity
1. Adaptation and Pre-testing	1	Adaptation of the English-language tools by a local team of 3 with language expertise to ensure appropriateness for the Kenyan context
	2	Review by 3 local mental health experts (independently) and discussion as a team
	3	Focus group discussions with 6 target adolescent groups: boys ages 10-14, boys 15-19, girls 10-14, girls 15-19, or 3 younger and 3 older adolescent groups (6 groups of 6-8 adolescents,(n=48)
		Focus group discussions with parents of 10-19-year-olds (2 groups of 6-8 parents, n=16)
	4	Back translation (by individual blind to original version of tool)
	5	Cognitive Interviewing with 2 adolescents in each of the 4 target groups above (n=8) and with parents of children ages 10-19 (n=8) and qualitative analyses of the cognitive interviewing, (total n=16)
2. Administration of the tool	6	Administration of the tool among selected sample of adolescents and parents of younger adolescents (n=250 adolescents (125 adolescents per site), n= 100 caregivers (50 parents per site)
3. Validation	7	Validation using diagnostic interview by trained mental health worker/ psychosocial counselors in both sites. Eight trained mental health workers will be used (4 per site)
	8	Statistical analyses to establish psychometric properties of the measures will follow the same procedure as the tools given in the proposal.

Inclusion Criteria for adolescents

- Seeking primary care at thecenter;
- ages 10-19years;
- ability to give informedconsent;
- availability for duration ofinterview
- intact cognitivefunctioning

Exclusion Criteria for adolescents:

adolescents over 19 years of age, and unable to give informed consent

caregiver refusing consent

inclusion criteria for caregivers

- agree to sign informed consent
- over 18 years of age
- intact cognitive functioning
- availability for duration of interview

Sample size

The desired sample size is determined with the goal of obtaining a sample sufficient to estimate the Receiver Operating Characteristic (ROC) curve and Area Under the Curve (AUC) for the tool to be translated, adapted, and validated. While a priori sample size determinations for AUC are highly susceptible to assumptions about the performance of the test (72), a sample size of 100 is generally sufficient to make a qualitative assessment of the utility of a test (73). Given the complexity and multiple assumptions involved, it is customary in validation studies to estimate sample size using comparison with previous validation studies with similar designs.

Data collection procedure

Pre-testing phase

The goals of this phase are to ensure appropriate adaptation of mental health measures for population-level measurement. This will be achieved through translation/adaptation and back-translation, qualitative data collection and analysis using focus groups and interviews.

Additional objectives of the research activities conducted in the initial phase are to gain an in-depth understanding of knowledge and beliefs surrounding adolescent mental health, including causal attributions, risks to mental and emotional well-being in everyday life, and help seeking. Culturally grounded elements of mental health, care and support, and of stigma will be explored. The qualitative data contributes independently to our understanding of culturally salient concepts of mental ill-health, and will improve the quality of the survey measures and ability of mental health interventions to best cater to the needs of adolescents.

Translation/Adaptation (STEP 1)

A team of 3 individuals with linguistic expertise will review the previously-validated English language tools. They will work together to ensure that the wording is adjusted to be easily and appropriately interpreted in the Kenyan context. Or, they may independently assess the instruments, and meet to discuss areas of discrepancy, ambiguous interpretation, or unclear phrasing. The overall process of translation and adaptation of scales will be guided by the ITC test translation and adaptation guidelines. A Translation Monitoring Form will be used to document the adaptation process and to track incorporation of recommended changes.

Expert Review of Translations (STEP 2)

The draft version will be presented for review to mental health experts. A second draft version will be created based on feedback from the expert panel. The mental health experts in the technical advisory board would assist us.

Focus Group Discussions (STEP 3)

Focus group discussions will be conducted with 4 target groups: boys ages 10-14, boys 15-19, girls 10-14, girls 15-19. It is anticipated to have roughly 6-8 adolescents in each group. This process will help in

refining the tools by getting feedback from adolescents is a direct manner as well as on and the applicability and sensitivity of the content. A "free listing" exercise may be conducted at the beginning of the focus groups to elicit commonly used words and expressions in the target groups, for example, terms like "sadness" and "worry". Areas of discrepancy, ambiguous interpretation, or unclear phrasing of questions in the tool will be discussed during the focus groups. The feedback received will be incorporated into the tool and documented in the Translation Monitoring Form.

The discussions aim to first, more generally discuss beliefs about mental illness and its treatment, notions of stigma, sources of support, etc., while the second section of topics will cover aspects of the specific measures and questionnaire items.

Mental health has many components, and is also culturally specific, hence we plan to carefully design the discussion guides, interviews, open-ended questions, and vignettes to uncover aspects specific to the setting. The discussions will tap into 'what other people think' about mental illness, care and treatments, and understanding in general surrounding mental illness and well-being. Mental health care utilization and mental health support will be approached with items about mental health services, and non-allopathic modalities, or with inquiring about help seeking behavior in the (fictious) event of depression or other mental disorder by a member of the household or by using a vignette to this effect. The team of investigators will work collaboratively with local and international experts to develop the discussion guides. Interviews and focus groups may be digitally recorded and transcribed verbatim, and may then be entered into a qualitative analysis software for coding.

Independent Back-translation (STEP 4)

Back translation of the tool for the original language/setting will then be conducted by someone blind to the original version, to ensure that the original meaning of items is maintained.

Cognitive Interviews (STEP 5)

Cognitive interviewing will be conducted with a total of 8 adolescents: 2 boys ages 10-14, 2 boys 15-19, 2 girls 10-14, 2 girls 15-19. These interviews will be conducted to garner their understanding of the questionnaire items and to validate the response options. The cognitive interviews will follow a semi-structured interview format. Techniques, such as 'thinking aloud' and verbal probing, will be used to ensure that items are correctly understood, and to identify problems with wording and/or difficulties with response options. The specific items in the questionnaire will be discussed, to confirm the validity of the item adaptation, assess suitability and interpretation of the response options, and to understand the process whereby their response was chosen. Areas of discrepancy, ambiguity, or unclear phrasing of items or response choices will be discussed during the interviews. The data gathered will be used to guide further adaptation or modifications to ensure reliability of data in the next phase of testing, and will be documented in the Translation Monitoring Form.

In order to reduce participant burden, participants may be encouraged to focus on items which they identified as being particularly difficult or easy to understand, or those which they feel could be rephrased to improve clarity. Each cognitive interview is anticipated to last 30 minutes up to 1 hour. Based on feedback obtained during cognitive interviews, a revised version of the questionnaire will be finalized.

The research team will work collaboratively to develop the interviewing guides, and as well to provide training to the research staff.

I. Questionniare administration and validation phase

Questionnaire Administration (STEP 6)

Survey questionnaires will be administered to 250 adolescents and 100 parents of younger adolescents. Surveys may be administered within the school context or at home, depending on school-year and feasibility assessment of the setting. The questionnaire will be administered by research assistants using manually the entire tool, including all optional test modules, is anticipated to take 30 minutes or less to administer. As part of this work all research assistants will receive basic tool administration training and be fluent in English.

A preliminary draft of the measures (instruments) planned for inclusion in the survey is provided in the Appendix 44-55. The final revisions of tools will be informed by the qualitative data results. Cards with written response options may be made be available to all respondents. Specific images and response items on the cards will have been previously validated during the focus groups and cognitive interviews. A selection of 50 participants will be asked to complete the questionnaire a second time, 1-2 weeks following the initial administration – to allow for test-retest analysis of the stability of the measures.

Validation Procedures (STEP 7)

Validation using diagnostic interview by trained mental health worker/ psychosocial counselor A sub-sample of 50 individuals scoring above the cut-off for each condition (anxiety, depression, suicidal ideation or attempt, and behavioral or conduct problems), and 50 or more healthy individuals (controls) will be re-contacted to be administered a diagnostic interview, such as the Kiddie-SADS. The K-SADS is prioritized as it can be administered by a child and adolescent mental health professional with any level of training, ideally mental health nurses, counselors, or social workers. Approximately 30 minutes to 1 hour is the estimated duration for each administration of the diagnostic interview, as only the portions relevant to this study will be administered (introductory interview portion, depressive disorders, anxiety disorders, attention deficit hyperactivity disorder, oppositional defiant disorder, conduct disorder. The diagnostic interview will take place in a room or office arranged for the study purposes, or location convenient to the participant, such as at a nearby clinic, a school or their home. The privacy and safety of the participant will be prioritized.Clinical calibration using K-SADS would be carried out by a team of county and MOH psychiatrists who are part of the study implementation work.

Primary outcome measures of the survey and validation phase include depression, anxiety, behavioral problems, suicidal ideation or attempt, and functional impairment due to any mental health condition. Secondary outcome measures may include health risk or protective behaviors, like substance use, physical activity, experience of violence or abuse, or bullying. Household variables may include socio-economic characteristics and composition of the household and household members, and other established risk or protective factors at the individual, family, or community level, that are relevant to adolescent mental health. **Participants with high scores will receive 4 sessions brief IPT to help manage some of their issues.**

III Data Analysis of qualitative data and quantitative data

Data collected during focus group via audio recordings will be transcribed verbatim and complemented by notes taken. A software package, such as Atlas-Ti, may be used to manage qualitative data generated

from participant focus groups and cognitive interviews. Thematic analysis and content analysis will be used to organize focus group data into meaningful themes, while a deeper analysis and investigation into concepts and constructs may ensue if time and resources permit. Such in-depth analysis of topics including local concepts, beliefs, or stigma, may enable a richer understanding of these aspects within the local context. The data analysis will follow the six steps to thematic analysis proposed by Braun and Clarke (74). Qualitative coding will also incorporate the a-priori themes for acceptability, feasibility, and comprehensibility as outlined by van Ommeren (75) and modified for use with children and adolescents.

Statistical Analysis

Quantitative data will be analyzed using a statistical software package. Analyses will be conducted to assess the performance of the tool using clinical diagnoses according to K-SADS as the gold standard. Analyses will also be conducted to assess the performance of individual questionnaire items. Diagnostic sensitivity and specificity, positive predictive value, negative predictive value, positive likelihood ratio, negative likelihood ratio, diagnostic odds ratio, Youden's index and reliability will be calculated. Curve analysis will be used to determine the optimal cut-off. Internal consistency of the subscales will be assessed. Cronbach's α values will be calculated for the total scale and subscales identified by factor analysis. As factor analysis is planned, to confirm meaningfulness of item clusters, sufficient sample size for this has been estimated. Values of 0.80, or higher, are commonly accepted as evidence of adequate internal reliability. An 'if item deleted' analysis will be conducted to identify whether any items should be dropped from the scale.

To assess test–retest reliability, intraclass correlation coefficients will be calculated on subscale and total scores which are expected to remain stable. Weighted Cohen's κ values may be also calculated to assess test–retest reliability at the item level. Coefficients will be established separately for children and adults, and on samples of approximately 50 questionnaires completed approximately 1-2 weeks after initial completion.

Intraclass correlations will be used to assess inter-rater agreement between pairs of scores from different raters (e.g., self-report vs. parent). Coefficients will be calculated at the subscale and total score levels. Weighted Cohen's κ values will also be calculated to assess inter-rater reliability at the item level.

Receiver operating characteristic (ROC) analyses will be conducted to evaluate the discriminative ability of the tools. Analyses will be conducted comparing to the presence of clinical diagnosis according to the Kiddie-SADS. In addition, independent samples t tests will be performed with clinical diagnoses to assess if there are differences in tool scores and assess the ability of the tools to distinguish between diagnoses.

3.4 Methodology for Aim2

(Develop adapted version of IPT-G/ and mhGAP-IG; and build local capacity for implementation).

Results from Aim 1 will be used to inform the adaptation of mhGAP and IPT-G contents, adolescents/ family members' engagement activities, implementation process, and training of the implementation team.

An adapted manual/implementation protocol for mhGAP-IG and IPT-G (full and mini versions) willbedeveloped,andadditionalfeedbackwillbesoughtfromAdvisoryBoardbeforeusingthese. The refined version of manual/protocol will be used for research team and MCH implementation teamtraining.

To **build research capacity**, I will work with my mentors to design and provide a short/intensive 3-day course on child mental health implementation methodology for research team members, relevant stakeholders, and graduate level students (n=20 in year 2).
We will be testing UNICEF's HAT framework for development of socio-emotional learning and depression mitigation modules for pregnant adolescents. This material will be part of the treatment as usual arm of the work. We will work with user centred design research to inform the development of an integrated mental health promotion and prevention intervention model for pregnant adolescents, based on the WHO-UNICEF Helping Adolescents Thrive Initiative (HAT). This is a low intensity, self-learning mental health information meant to promote mental health. with this material, we would not have to develop enhanced treatment as usual materials by ourselves.

Importantly, the intervention will also aim to build a community of support and identify a system of local resources for pregnant adolescent girls. Towards, this consultation with other key stakeholders will be held to inform intervention models that are culturally relevant, embedded within the local system and structures, and promotes community-based awareness around support and empowerment of pregnant adolescents. This may include civil society organizations that have experience working with pregnant adolescents/adolescent mothers.

Relevance of the work
This work is of immense local and global relevance for its promise to be both locally validated measurement of adolescent mental health and its immediate use for pregnant and parenting

adolescents that can be used at a later stage for national level data collection and programming efforts. Prevalence estimates and qualitative findings would also inform discussions around sub-syndromal conditions as well as how younger adolescents and their caregivers perceive symptoms and distress associated with depression and anxiety. Hence, society will reap a number of benefits will result from this research, not the least of which is better understanding of adolescent mental health conditions in settings where very little is known at present using culturally validated tools. This new knowledge will further help bolster the ongoing study efforts to provide depression and common mental health disorders support and services for this population. This work will also inform global efforts to support adolescent mental health efforts and provide evidence for policies and programs.

To a) build service capacity for adolescent perinatal depression, we will train MCHimplementation team (n=16 from2 MCH clinics) for task-shifting/sharing collaborative approach of mhGAP-IG and IPT/G implementation. Members of the implementation team (to bedetermine in Aim 1, which may include family members/adolescents if peer leader/empowerment implementation strategy will be used) will receive *a 4-day training* (including 1-day practice training) to prepare them for the implementation. Additional *8-group coaching/supportfromtheclinicalteam(providedaftereachIPT-Ggroupsession)*duringthe1st interventionimplementation period will also be provided. The coaching support after the 1st implementation will be based on theneeds.

b) To ensure meaningful engagement of pregnant adolescent girls and a human centred design process, a series of consultation workshops will be held to review data collected and jointly uncover insights to inform key components of a Helping Pregnant Adolescent Girls Thrive Intervention model. The model will seek to enhance the ability of pregnant girls to cope with adversity and prevent substance use. ANC/PNC platforms will be leveraged to provide interactive learning, skills-building, mentoring, and peer support towards the adoption of 4 core practices 1) Emotional regulation and stress management 2) Problem solving 3) Interpersonal skills and 4) Alcohol and drug use prevention.

c) This work will be part of the COVID supplement to K43 in September 2020 for one year. We will first focus on developing e-training modules (1-4 months), and then focused on user-centered testing for the adopted mhGAP and IPT-G E-training modules (months 5-8) to ensure user-centeredness, acceptability, and feasibility of the e-training approach. The e-module contents will be adopted from our current face-to-face version of mhGAP/IPT-G developed from K43. Table 1c outlines the contents and number of e-modules that we anticipated to create. To ensure user centeredness and optimization of the e-training, user-centered testing will be conducted with 20 MCH health workers and relevant service staff. We will conduct user-centered testing separated for mhGAP and IPT-G eTraining modules. The eHealth Technology Acceptance Model (TAM)(76, 77) that focuses on understanding contextual factors that influence perceived usefulness and ease of use of the remote/digital components will be used to guide the study. An iterated feedback-and-improvement process will be applied. Both

quantitative and qualitative feedback data will be gathered, and results will be used for e-training content and quality improvement.

Table 1c. Proposed Digital Products for mhGAP/IPT-G e-Training and Processes

mhGAP/ IPT-G Contents to be adapted to an eHealth version	Number of module/videos	Outcome and description of products
mhGAP – community toolkit	1 video	Short video, aiming at introducing key mhGAP messages of UHC, integration of mental health in primary care, psychological first aid and human rights. The video would be under 7-8 minutes.
mhGAP depression, essential care and practice, anxiety, stress, child and adolescent mental disorders, self harm, substance use, other conditions, epilepsy	9-10 videos	The videos will cover these 8 priority conditions, presented using mhGAP materials that are freely available and contextualized using culturally relevant explanations and animated characters. All videos will be less than 10 minutes. These will cover principles of physical and mental and neurological assessment followed by basic interventions and referral discussion. For adolescents, a module that includes interviewing peripartum adolescents will be included (which focus on adolescents' life story and journey expectations and how mental disorders start to form).
Engagement/Motivational interview and orientation to psychotherapy	1 video	We will introduce this video to develop basic skills and competencies in psychotherapy and engagement with the participants
Group interpersonal psychotherapy	5-6 videos	The group IPT video would cover a series of themes – Core areas of IPT<br>• Assessment and pre-group phase<br>• Initial phase<br>• Middle phase<br>• Termination phase<br>• Signs and areas of concerns<br>• Group as a containment and mobilizer of resources<br>• Dos and dont's

d)After the user-centered testing (about months 9-10), the full version of integrated E-mhGAP/IPT-G training will be pilot implemented and assessed for impacts. Two to three MCH, primary care, and/or community-based service agencies that we have partnership with, have been impacted by COVID-19, and have not received mhGAP/IPT-G training previously will be prioritized. The research implementation team will play a technical support role in supporting agencies to set-up and use the e-training modules/program. Proctor's *Implementation Outcome Framework* (56) will guide our evaluation plan, which includes implementation, service (agency), and patient/user outcomes. We will gather both quantitative and qualitative data from staff in the participating agencies (n=12) and randomly selected health care workers who offer services in those settings (n=45). We will also conduct an FGD with peripartum adolescents aged 13-18 years on the suitability of the e-training content that will ave been developed for health care workers to try and understand if it would address some of their service gaps and needs (n=9; 3 participants per facility). Qualitative feedback related to barriers and facilitators of e-version of mhGAP/IPT-G training and care will also be gathered for future training content and implementation process enhancement. The participating agencies will be Kenyatta national hospital, Mathari national teaching and referral hospital, Nairobi county's-, Ngara health center where previously permissions were given for work.

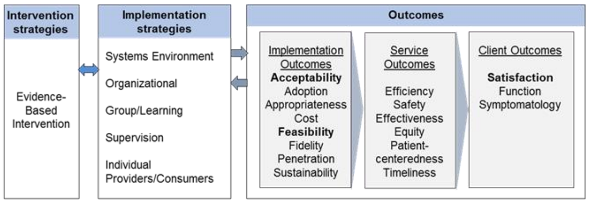

Bolded: outcome assessed in Aim 2 pilot

Data Collection Measures and Procedures: a) Quality and impacts of training will be assessed through attendance tracking, after training satisfaction evaluation, pre- to post-training knowledge/competenceassessmentwithtrainees.Themeasureswillbedevelopedduringthestudy period and adapted from my mentors' previous D&I studies(78).

Table 1d: Steps and activities for HAT adaptation for peripartum adolescents

Planned steps and activities under HAT adaptation for peripartum adolescents	
Step 1 Adolescent peripartum mental health design process	design process consultation with INSPIRE mentors, MOH, Nairobi county, UNFPA, WHO, UNICEF and KNH
	development of adolescent-centred design training for study staff and partners N=20
	training on UCD/HCD focusing on HAT and pregnant adolescents N=20

development and user-centred design	
Step 2 Qualitative and priority setting studies	stakeholder engagement on identifying key themes on peripartum adolescent mental health pegged on INSPIRE CAB and TAB N=45 plus MMAP MH and counseling teams
	ANC/PNC adolescent mental health integration consultation - delphi/priority setting exercise Pumwani, KNH and Mathare hospitals and Nairobi county N=40
	training staff in qualitative data and UCD
	UCD/HCD interviews on HAT with pregnant adolescents N=25
	UCD/HCD interviews on HAT with adolescents mothers N=15
	UCD/HCD interviews on HAT with HCWs and policy makers- adolescent health and mental health specialists N=20 across KNH, Pumwani, Mathare and Nairobi county PHCs
	data analysis of qualitative work
Step 3 quality of care and HAT adaptation work	developing a quality of care focused and a policy or HCD focused report based on qual analysis
	presentation of study findings to CAB and TAB
	adolescent led design workshops in partnership with civil society members, county health and MOH to integrate and present findings from above
	clinical workflow and quality of care evaluation keeping HAT in mind consultative meeting N=30
	synthesis of findings of various consultations
	HAT pregnant and parenting adolescent recommendations for implementation

b) Outputs and Impacts: Findings will provide actionable evidence for technology approach of mental health capacity building and service set-up. This study will generate new knowledge on: (i) user-centered strategies to provide an effective e-mhGAP/IPT-G training; (ii) better understand whether the web/video/eHealth approach of training overcome pre-existing and COVID-19-related implementation barrier; and (iii) better understand whether the e-training of mhGAP/IPT-G can mitigating negative effect of COVID-19 on community members' mental health.

Study Design and Approach.
Table 1e outline the research activities and study timeline for the proposed supplemental grant (12 months).
Research Team. Dr. Kumar (the PI of the current K43 and developer of the Kenyan version of mhGAP/IPT-G care for adolescents), her primary mentors Profs Mary McKay and Caleb Othieno would help review the user-centered design formative work and e-training content development and her mentor Prof. Huang with eHealth design, implementation expertise, and eHealth research network will provide

support in the development of e-training and implementation. The technology consultant and from Kenyan MOH will provide technical expertise in e-Module videos creation. Our partners MOH and Nairobi county (Drs Simon Njuguna and Dr Carol Ngunu) will serve as policy and e-health service design experts/consultants to ensure the approach and modules are in-line with Kenya policy and MOH/public health priority.

Table 1e: Study Timeline & Activities (in Months) for COVID supplement

	1-2	3-4	5-6	7-8	9-10	11-12
mhGAP/IPT-G adaptation to e-Modules & training video development	X	X				
User-centered testing for *mhGAP* E-training & content enhancement (1 MCH; n=10)			X			
User-centered testing for *IPT-G* E-training & content enhancement (1 MCH; n=10)				X		
Pilot implementation and testing mhGAP/IPT-G e-Training in 2-3 existing partnering MCH, primary care, and community-based mental health service settings (n=30)					X	
Mixed method pre-post evaluation with implementation site agency staff (n=12) and randomly selected participants (n=45), FGD with adolescents ages 13-18 years on suitability of content (n=9)					X	X
Results and e-version mhGAP/IPT-G training program dissemination						X

Analysis Plan**.** *Hypothesis*: a) We anticipate capacity building will significantly improve trainees' (nurses and community health workers) knowledge and skills in D&I research or depression intervention implementation. To test hypothesis, qualitative training notes and feedback data will be documented. Themes related to partnership and lessons learned for D&I capacity building will beanalyzedandreported forimprovementinfuturecapacitybuildingapproach.Quantitativedatarelated to knowledge and competence will be analyzed using pair-t tests and repeated measuresto explore impact across different types of MCHpersonnel.

b) For HAT adaptation and user centred design work – the process would be largely qualitative involving data analysis of focused group discussion and key informant interviews. We will use user-centred design perspective along with adolescent mental health service frameworks to develop themes and adaptation of clinical workflow process. We will use grounded theory and qualitative data analytic packages like Atlas-Ti to analyze the data.

3.5 Methodology for Aim3

(Test feasibility and impacts of the adapted version of IPT-G and mhGAP-IG).

To evaluate implementation-effectiveness of the mhGAP-IG (including IPT-G), we will apply a *hybrid effectiveness-implementation* design (79) and *randomized control trial (RCT*). The evaluationconstructswillbeguidedbythe*ConceptualmodelofImplementationResearch(Figure2)* (56), which consider implementation, service, and patient outcome indicators. Primary outcomes will be focus on acceptability, engagement with the interventions, and effectiveness on adolescents'depression.

***Data Collection Measures, and Procedure*.** The 2 MCH clinics that we build capacity in Aim 2 willbethepilot Implementationsites.Duringthe1st implementationcycle(about2-3months),we will work with only 1 MCH clinic (Cohort 1), and then work with another MCH clinic in the 2nd implementation (Cohort 2). For each cohort, 45 pregnant adolescents with moderate to severe depression will berecruited.

Intervention Implementation. Considering high prevalence of adolescent pregnancy, high risk for depression in this population, and low resource in MCH clinics, the WHO recommended full version of G-IPT may not be practical in meeting population service demands and needs. Therefore, we will test a Mini version of G-IPT. Recruited participants will be randomlyassigned (usingcomputerrandom generatorbyresearchstaff)tothreeinterventionarms—IPT-Gfull,IPT- G mini, and wait-list control, n=30 for eacharm).

- The IPT-G full and mini groups will receive the assigned interventions (8 vs. 4 sessions) developed from Aims 1 & 2 (including a perinatal depression educational material/handout). To avoid contamination, intervention for the IPT-G full and mini versionswillbecarriedoutby2separateimplementationteams(2-3facilitatorsineach team) follow the assigned manual/protocol. Table 1 below provides intervention contents for the mini version, in comparing to the recommended long version. Wewill study implementation-effectiveness for bothversions.
- The Control will receive standard care (with referral) and perinatal depression educational materials for safety concernsthat we would have adapted from HAT work carried out in Aim 2. They will receive *IPT-G mini* after the post implementation assessment iscompleted.

Table 2. Intervention content by session and overview of full versus mini versions of G-IPT

IPT-G Full Version (8 sessions)	IPT-G Mini Version (4 sessions)
1. Recognize and start dealing with current depression<br>2. Understand links between current depression and IPT problem areas; conduct an Interpersonal inventory	1. Reviewing the context of depression, understanding interpersonal basis of depression, problem-solving, communication analysis and evaluation
3. Decide with the person on the interpersonal problems that are linked to the current depression, invite the person to join the IPT group and discuss goals and rules<br>4. Introduce the group members and talk about depression	2. Reviewing symptoms and functioning, understanding- grief, disputes and transitions especially arrival of baby and changes in body and social role, learning strategies to deal with these and evaluation
5. Discussion depression and the IPT problem areas – grief, role transitions and role disputes that the group members are facing<br>6. Discuss how the group willwork on those areas	3. Reviewing symptoms and functions, preparing for birth and making plans for baby and self-care, understanding value of communication and support, reviewing depression and evaluation
7. Use strategies specific to each IPT problem area<br>8. Review what has happened during treatment, and for group members to say goodbye to each other and the facilitator. make plans about how to address problems that might arise up or newproblems that emerges	4. Reviewing symptoms and functioning, identifying interpersonal strengths, support and strengthening communication, health facility engagement, plans for post baby and mother care, and depression evaluation

Measures and Procedure. To evaluate implementation and effectiveness outcome, a mixed methodsdatacollectionapproachwillbeapplied,anddatawillbecollectedfrommultiplesources (i.e.,implementers,MCHleaders,adolescentmothersandtheirfamilymembers)tominimizedata collection bias. Three areas of outcomes will be evaluated (see Table 2). Both *quantitative data* (focusing on adolescent mothers' effectiveness outcomes, implementation fidelity, and MCH serviceoutcomes)and*qualitativedata*(focusingonimplementationprocessandcontextualfactors that influence implementation and experience) will be collected through focus groups (2 groups for each intervention arm) at the end of implementationcycle.

All study participants will complete a baseline (Time 1) interview before randomization and implementation, followed by a 1 or 2-month implementation period (2 months for the IPT-G full and 1 month for the IPT-G mini). They will receive a post-intervention interview assessment at the end of implementation cycle (Time 2, about 3-4 months after the Time 1). Implementation outcomedata,collectedfromtheMCHstaff,willbegatheredaftereachgroupsessionthroughout the two implementation periods. To facilitate coordination and program implementation, participating MCH will be provided with two tablets and a small incentive which could offset implementation costs.

Table 3 Study Measures for the Evaluation Study: Constructs, Informants, Assessment Schedule, and Assessment Tools.

Constructs, Informant, & Assessment Time	Assessment Tools/Measures
Multi-level D&I Context (domains listed in Figure 1) *[Time 1]*	
**Inner setting &Individual Characteristics(MCH staff, Adolescent reports)):** ♦ MCH system characteristics; ♦ Implementer demo & attitude about the EBI; ♦ Family characteristics	Provider Environment Questionnaire Survey (72, 73); Organization climate/Readiness Questionnaire (107); EBP Attitude Scale (74); Family Demographics /social determinants[2]
Implementation Outcomes *[Assessed Throughout Implementation Period]*	
**a) Fidelity (Staff):** % of content provided; **b) Engagement (staff report)**: targets'engagement **b) Quality of Implementation(adolescent-report)**: ♦ Satisfaction;	**a**: Fidelity Checklists[3].**b**: Program Engagement; **c.**Satisfaction[4] ;
FBO Pediatric Mental Health Service Outcomes *[Time 1, Time 2]*	
**a) MCH service quality (adolescent mothers):** patient satisfaction**; b) MCH climate (staff report)**: support, trust, communication about adolescent health.	**a:** Service quality; **b:** Organization Climate Questionnaire)[7,8]
mhGAP/IPT-G Effectiveness Outcomes*[Time 1, Time 2]*	
**a) Adolescent mental health outcomes (Primary) (adolescent report); b) family functioning outcomes (Secondary outcomes) (adolescent & caregiver report):** functioning, depression, trauma, adjustment, social support, self-efficacy, stress, family interpersonal communication.	**a**:WHO-Disability Assessment Schedule; Edinburgh Postpartum Depression Scale; Patient Health Questionnaire-9;**b:** Self-efficacy; Social support; PTSD Patient Checklist-Civilian; Kessler Psychological Distress Scale; Adolescent Interpersonal Connectedness and Conflict Inventory(73, 74, 75, 76, 77, 78, 79, 80, 81)

Power Calculation. The sample size (n=90) for the effectiveness evaluation is determined based onmediumtolargeeffectsize(d=.70-1.5)oftheIPTinterventionreportedintheliteraturebased on LMICs (assuming 80% power of two-side significant test with α=.05). Because the primary effectivenessoutcome(i.e.,depression)willbebasedonadolescentreport,weanticipatethegroup nesting will be very low (ICC close to 0) as evident from my mentors' prior research experience and data reported in the literature. Also, adolescent mental health outcomes will be assessed at 2 time points (at baseline and post intervention). For the estimated sample, we will have adequate power to detect impacts that are meaningful and realistic for the Full G-IPT (medium to large effects). We may not have sufficient power for the mini version of G-IPT (as we anticipate the effect size may be smaller). Given this is a pilot implementation-effectiveness study (withlimited researchbudget),theproposedstudywillprovidedataforestimatingthesizeofinterventioneffect,which will be used to do more accurate power estimation for the R01 application (see Table 3 for the

detectable effect sizes with a range of ICCs).

Table 4. Power estimation for the primary effectiveness outcomes (adolescent depression)

ICC	Design effect	Power Estimate for Post Intervention	
		Effective sample Size total (N* for 1:1 two group comparison)	Detectable Cohen's d
0.00	1.00	60	0.74
0.01	1.14	53	0.78
0.03	1.42	42	0.89
0.05	1.70	35	0.96

Note. In cluster RCT, the detectable effect sizes depend on the usual study design parameters, cluster sizes and the ICCs. The clustering usually leads to a decrease of the actual sample size (N) by a factor called Variance Inflation Factor (VIF). The effective sample size (N) in the cluster RCT is N*=N/VIF, where VIF=1+n(m-1). Estimations of detectable effects reportedhere consider: 1) ICC (intervention group level) will be small (range from 0 - 0.05); (2) each arm with N of 30, average group size 15; and (3) comparison between mini G-IPT vs. control or between Long G-IPT vs. control (with N of 60 for each pair ofcomparison).*

Analysis Plan**.** Data collected through 2 cohorts will be analyzed. Hypotheses to be tested include:

- relative to control, pregnant adolescents in the IPT-G will have better outcomes (on Primary andSecondary);
- both IPT-G full and mini versions will be effective, but slightly high impacts for the IPT-G full version;and
- Intervention implementation using task-shifting/sharing approach will have be highly acceptable, engaging, and have high fidelity (for both full and miniversions).

Qualitative data will be transcribed and analyzed using similar techniques described in Aim 1. Wewillreviewallfocusgroupdiscussiondataandengageinanopen-codingprocesstosearchfor emerging themes. Analyses for mhGAP/IPT-G effectiveness will use **quantitative data** and estimate size of effects after the implementation. The analyses will be based on multi-level modelling, adjusting for family/group nesting effects. Post-intervention outcomes will be measuredasafunctionofbaselinelevelsofthecorrespondingoutcomevariablesandintervention status. To evaluate feasibility and quality of implementation, we will review all available data, including multiple sources of feasibility, acceptability, engagement, alliance/ interaction data. I willbetrainedinD&Irelateddataanalysisduringmy Ktraining,andDr.Huang(primarymentor) who has expertise in quantitative analysis will support me on dataanalysis.

3.6 Locations/Facilities.

We will test the**aim 3**intervention in MCH clinics in primary care and aims 1-2 will include Pumwani, KNH and MNTRH and Ngara primary care clinicin Nairobi, Kenya.

3.7 StudyParticipants

A total of 1234subjects will participate in this study (across 3 study aims).

Aim 1: Participants for studying "fit" of the mhGAP/IPT-G and understanding multi-level barriers/strategies (Aim 1; n= 80 diverse stakeholders). To study potential barriers andstrategies for promote utilization and impacts of IPT-G and mhGAP, we will conduct 8 focus groups with representative diverse stakeholders (i.e., including two groups for pregnant adolescents and new adolescent mothers, two groups for adolescent caregivers and partners, two groups for MCH staff, one group for community advocates and policy leaders, and one group for child and adolescent mental health professionals).

Aim 2: Implementers for mhGAP/IPT-G intervention (Aim 2; n=36 provider subjects). To build research capacity, we will provide a short/intensive course on child mental health implementation methodology for research team members, relevant stakeholders, and graduate level students (n=20 in year 2). To build service capacity for adolescent perinatal depression, we will train MCH implementation team (n=16 from 2 MCH clinics) for task-shifting/sharing collaborativeapproachofmhGAP-IGandIPT/Gimplementation.Qualityandimpactsoftraining will be assessed through attendance tracking, after training satisfaction evaluation, pre- to post-training knowledge/competence assessment withtrainees.

Aim 3: **Participants for mhGAP/IPT-G intervention Evaluation Study (Aim 3; n= 90pregnant adolescent subjects).** To estimate impacts of the intervention, 90 adolescents and their caregivers from 2 MCHs will be recruited for the intervention evaluation study.

Inclusion criteria. (For the feasibility study)

- Pregnant adolescents (ages13-18)
- Screened with moderate to severe depression during the 1st to 2nd trimester (defined as Edinburgh Postnatal Depression Screen (EPDS) score $\geq$ 13 or meet the APA's Diagnostic and Statistical Manual for Mental Disorders IV-TR version (DSM-IV-TR) criteria for major depression disorder and perinataldepression),
- Who agree to give informed consent (by adolescents and their adultcaregivers)
- Are willing to come for group sessions at MCH clinic,and
- Can participate in evaluation assessment will beeligible.

Exclusion criteria

Participants will be excluded from the study if they:

- Suffered from or showed evidence of severe personality disorder, acute psychosis, suicidality,or
- Where there was significant substance abuse, or evidence of severecomorbidities.

Recruitedpregnantadolescentsandtheirprimarycaregivers/partnerwillbeinvolvedinevaluation researchactivities,andinvitedtoattendtheIPT-G.WewillworkwithMCHstafftodeterminethe final recruitmentstrategies.

4. Ethical Considerations

***Table 5 summarizes the characteristics of the human subjects*.**

Characteristic	Definition
Main study endpoint	To integrate implementation strategies (identified from Aim 1) Into IPT-G (modified version) and mhGAP-IG depression care modified workflow and estimate implementation-effectiveness in pregnant Kenyan adolescents**.**
Projected Enrolment	180 pregnant adolescents in their 1st-2nd trimester
Age	13-18 years
Source of subjects	Kariobangi and Kangemi Health centers
Follow-up period	12 months
Specimens	n/a
Intervention	Group psychotherapy enhanced with identified implementation strategies (knowledge promotion, peer mentoring, engagement)<br>Training Health workers in delivering this intervention
Exclusion criteria	Severe neurologic defect (unable to participate in group therapy), severe substance abuse, intellectual impairments
Exit	Refer back to primary care program

4.1 Recruitment and enrolment:

A2. *1) Recruitment and enrolment:*

PregnantadolescentsvisitingtheANCclinicsatthe*Kariobangi*and*Kangemi*healthcenters and *Pumwani* (Referral)would be recruited for the study. At these sites, study staff will identify eligible adolescents and invite boththeadolescentsandtheircaregivers(orpartners)whoshowinteresttoparticipateinthestudy. The purpose of the study will be explained to them in English or Kiswahili or in the local dialect they may be using. They will be asked to provide written informed consent for the adolescents' enrolment. Consent will be obtained from all parents/guardians and assent from the adolescents prior to enrolment into the study. Snowballing might be used for recruitment if an adolescent or caregiver or health worker associated with the sites know a pregnant adolescent who could participate.

A2. *2) Population justification:*

Adolescent pregnancy is a big issue in Kenyan society due to its implications for HIV/AIDS

infection spread, poor sexual and reproductive health choices, gender-based violence and high-riskbehaviorsareconsequencesofadolescentpregnancy.Perinatalmentalhealthservicesarevery scantforthisvulnerablegroupanditisimportanttocomeupwithintegratedmentalhealthservices within primary carefacilities.

A2. *3) Collaborative Sites:*

The study will be conducted in Nairobi at the Nairobi County's two primary health care facilities that offer MCH services. It will be a collaboration between the University of Nairobi, partners from Ministry of Health – Community Health Promotion and Mental Health Departments, WHO non-communicable diseases unit, UNFPA's adolescent SRH section. After enrolmentadolescents willbefollowedupforregularclinicvisitsinthetworespectivecenters.Thestudyclinicwillhave dedicated clinical staff including and medical officers able to provide clinical care offered as part of routine Antenatal Clinic (ANC) care for pregnant adolescents. The assumption is that the pregnant adolescent will seek the intervention and ANC at the clinic site from where she was recruited. The delivery options might vary depending on the personal situation, obstetric history and medical complications associated with pregnancy. However, the participants will be tracked on the delivery and birth outcomes wherever child birth takesplace.

A2. *4) Data collection, Management and Protection:*

Collection: Data for the proposed study will be collected and managed onsite in Nairobi respectively. Data will be abstracted form MCH records as well as collected pre-and-post intervention from participant and caregiver interviews and surveys, provider interviews and surveys and other key stakeholder interviews. IPT-G related assessments and evaluation will be conducted utilizing questionnaires and FGDs and individual interviews.

*Management:*Adedicateddatateamleadbyadatamanageranddataclerkswilloverseetheentry, management, monitoring and reporting of data. The University of Nairobi, and key mentors from Washington University at St Louis and New York University will provide technical support to support datamanagement.

*Protection:*Allclinicfileswillbesecuredthroughtheuseoflockedcabinetsinanentry-restricted office and computer pass word protection. Study records will not contain participant identifiers while clinic records which are used for participant intervention and management will have such identifiers. A link-log which identifies a subject's identifying information to a study code will be kept under lock and key and preserved for 7 years after completion of the study after which time it will be destroyed. All databases will be passwordprotected.

4.2 Potentialrisks

B.1. Confidentiality: There is minimal chance of loss of confidentiality of participants recruited in the intervention. However, Nairobi county and the University of Nairobi sites have experience of > 10 years in conducting clinical trials with women and children with a variety of health conditions and on health services research. All study personnel to be involved have completed coursesintheprotectionofhumansubjectsandwillreceiveroutinetraininginresponsibleconduct in carrying out research with adolescents and on peripartumcondition.

B.2.DisclosureofDepressionstatusormentalhealthcondition:Theparticipantswillbeinformed abouttheformalassessmentfindingsinasensitiveandconfidentialmannerensuringthattheyare offered support once depression diagnosis has been made. Any other identified mental health condition would also be shared and the participants would be offered the right referral. A consultant psychiatrist, clinical nurse and a social worker associated with the health facility the participant was registered at will facilitate furthermanagement.

*B.3. DisclosureofHIVstatusandPMTCT:*Inthecourseofrecruitmentofparticipantsifwecome across a participant who is HIV positive and was not aware of her status we would put in place steps to ensure that they are connected to the PMTCT and VCT facilities. We will also facilitate the process of disclosure related counseling. Referral to Kenyatta National Hospital’s PMTCT would be made if the facility level management at PMTCT is notadequate.

B.4. Participants with exposure to GBV or any form of intimate partner violence: any participant with exposure to GBV or any form of violence with partner or caregiver would be linked withthe GBV services at Kenyatta National Hospital. The obstretric/gynecology specialist along with clinical nurse at the facility would physically review the participant and the social worker will be involved to make furtherreview.

4.3 Adequacy of protection of human subjects

C1. *Informed consent:*

The study staff and the clinical teams taking care of the adolescents will jointly identify potential studysubjects.Thestudystaffwillexplainthestudyindetailtopartners/guardians/caregiversand pregnant adolescents and invite them to participate. Written informed consent will be obtained in eitherEnglishorKiswahilidependingonthepartners/guardiancomprehensionofeitherlanguage. Kiswahili is Kenya’s national language while English is the official language. As emancipated minors, pregnant or parenting adolescents can also give their full consent/assent toparticipate.

C2. *Data/Confidentiality:* Besides data being kept in locked cabinets or in password-protected computer databases, all study staff either have already been or will be trained in Protection of human subjects and Good clinical practice.

C3. *Engagement interview*: During the consenting period an appointment for an engagement interview would be sought the purpose of which would be to explain the intervention in details and identify the barriers to care. One research and clinical staff will carry out the engagement interview and will be trained in protection of human subjects as mentioned before.

4.4 Potential benefits of proposed research to human subjects andothers.

D1). *Direct benefits:*

Duringthestudyperiodalladolescentswillhaveaccesstocounselingandmedicalcarebyahighly trainedteamleadbyapediatrician,obstretric/gynecology specializinginperipartumhealthofadolescentsand two consultant psychiatrists. An experienced psychiatrist in addressing peri-partum mental health and the counseling team will address psychosocial concerns and challenges faced byfamilies.

D2). *Indirect benefits:* Adherence to psychopharmacological drugs, antiretroviral drugs may improve due to the intensive clinic schedule allowing for adherence to be probed and supported while high risk behavior including risk of unsafe sexual behavior, exposure to GBV or other domestic violence related exposures may be reduced. At a very vulnerable time during their development critical mental health, medical and support would be offered.

D3). *Importance of knowledge*: This study will add knowledge about adolescent sexual and reproductive health challenges, it will bring in multi-stakeholder perspectives (parents, other caregivers, partners, health providers, specialists and policy makers) on depression during adolescent pregnancies and post-delivery. This would enable us to better understand the barriers and facilitators of adolescent mental health. Infusing WHO's recommended care package with novel culturally and developmentally sensitive implementation strategies would bring about new knowledgetobothmentalhealthinterventiondevelopmentandimplementationanddissemination science fields.

4.5 DataManagement

A. Data and safety MonitoringPlan.

A1). *General Purpose of the DSMP:* Adverse events occurring in any study participants will be reported to the Kenyatta National hospital and University of Nairobi institutional review board within48hoursofoccurrenceandtheexternalreviewboards(advisorycommitteemembersofthe K43 proposal and key mentors at University of Washington St Louis and New York University) within 72 hours. On a monthly basis the study team will review summary reports of the adverse events. A Data Safety and Monitoring Board (DSMB) consisting of social workers, obstetrician/gynecologist, pediatricians, ethicist and biostatistician will be established and review interim comparisons of the trial arms at 3 and 6 monthly intervals. The general purpose of the DSMP is to maximize the safety and privacy of all study participants, and ensure the integrity, validity,andconfidentialityofthedatacollectionandanalysisprocedures.Theprotocoldescribed herein details the integration of the goals of the DSMP into all aspects of study design, implementation,andreview.ThePIwillreportanyseriousandunexpectedadverseevents,orany problems that involve risk to the participants or others, to the Nairobi city council operational research review board and the Kenyan IRB. The PI will report any serious adverse events, along with the Kenyan IRB review response/outcome, to theNIH.

A2). *Protection of Research Participants:* The protection of the rights of research participants is ensured by several aspects of the study protocol. First, the principal investigator and all members of the study staff will complete training in human subjects' protection, as required. This training consists of completion of the formalized training program sponsored by the KNH/UoN IRB as well as ongoing training by the PI in all aspects of human subjects' protection through NIH and her mentors' institutions where she will undergo training. Second, all study procedures are reviewed with staff, mentors and advisory board members from the perspective of ensuring the protection of the rights of study participants. This includes training of study staff in consent and

enrollment procedures to minimize coercion and ensure the principles of informed consent, maintaining study material and information in order to protect study participants' privacy and confidentiality, and ensuring that assessment procedures are conducted in a manner that protects study participants' privacy and rights. In addition, the study advisory board and KNH/UoN IRB will inform study participants about protection of their rights by independent institutional board, that would have approved the study. Third, both study participants and staff will be made aware ofthelimitstoconfidentiality.PregnantadolescentsandMCHstaff/CHWswillbefullyinformed of these limits at the time of consent. Study staff will be trained in the requirement to adhere to legal statutes regarding reporting to child protection agencies information obtained in the course of the study that leads a staff member to suspect that an adolescent is at risk for physical abuse or neglect or is being abused or neglected. There is a protocol detailing the responsibility of staff memberstoshareinformationwiththePIimmediatelyorassoonaspossibleaftertheinformation has been obtained. The protocol requires review of the information to determine whether a report to the protection agency is required. Finally, in the event of a clinical emergency or other crisis, research staff will be trained in basic crisis management including obstetric and pediatric emergenciesandabletocontactthePI.Staffwillbetrainedinamanualizedprotocolforhandling a variety of crises that may present to staff at the health facility or the community venue during interviews. The protocol for handling these issues addresses the safety of both study participants and study staff. The protocol also details the need for supervision in allinstances.

A3). *Integrity of Research Data*: All information collected from study participants during the course of the project will be kept confidentially. This includes information collected and storedin writtenandelectronicform.Asindicatedpreviously,datastoragealsoensuresintegrityofresearch dataandrightsofstudyparticipants.Papercopiesofidentifyinginformation,assessmentmeasures, and other study materials will be maintained in locked research files in locked offices. Electronic data are secured by server maintenance that includes password protection, limited access to data bystaff,differentlevelsofaccessdependingontheperson'sspecificlevelonthestaff,andserver securities, all of which ensure a high degree of protection from unauthorized users. Information will be coded by participant identification number. Linking of identifying information toresearch data will be kept to a minimum and even then under key and lock and only accessible to the PI. The identity of study participants will not be revealed in presentations or publications of study findings.Participantswillbegiventheopportunitytoconsentto(ordecline)theuseofanyspecific data collected from them during the intervention phase for use in presentations orpublications.

A4). *Data Monitoring:* The project staff will continuously evaluate the experience of study participants, with particular regard to participant reactions and risk exposure. Trends in data and findings will be examined periodically (yearly) in order to identify any changes in risk/benefit ratios that might necessitate a modification of the assessment protocol. In the unlikely event that monitoringofdatarevealsunanticipatedorotherwisenegativefindings;thePIwouldreportthese tothefieldintheformofapresentationorpublication.Furthermore,itwouldbecriticaltoassess, to the degree possible, what characteristics of the study protocol contributed to thefindings.

A5). *Progress Report*: Each year, the PI will prepare a report that summarizes the following:

- All serious and unexpected adverse events or other unanticipated problems thatinvolve risk to study participants or others and whether these appeared related to the research assessmentprotocols.
- Documentation of the reporting of all serious and unexpected adverse events to the KNH/UoN Institutional Review Board and the Nairobi County OperationalResearch Committee, as well as the response to and outcome of that report by theIRB.
- A summary of ongoing efforts to ensure participants' safety, privacy, andconfidentiality.
- A description of the way in which research instruments have been administered in a uniform manner and in a way that maintains participants'confidentiality.
- A summary of the study's progress toward recruitment goals, quality of data(e.g., appropriate completion of forms) and participationrates.
- Any changes in risk/benefit ratios that might necessitate the modification of theprotocol. Specific recommendations for protocol modifications will be elaborated, with the accompanyingrationale.

These yearly reports will be filed with the KNH/UoN IRB and with the NIH Project Officer and in consultation with the PIs mentors.

*A6).ExperiencetoDate:*Duringourpreviousstudies,noseriousoradverseeventswerereported. There were no complaints or untoward consequences from any of the aspects of the study, including the assessmentprocedures.

References

1. Chowdhary N, Jotheeswaran AT, Nadkarni A, et al. The methods and outcomes ofcultural adaptations of psychological treatments for depressive disorders: a systematic review. Psychological Medicine. 2014;44(6):1131-1146.
2. Neal SE, Chandra-Mouli V, Chou D. Adolescent first births in East Africa:disaggregating characteristics, trends and determinants. Reproductive Health.2015;12(13).
3. UNFPA. Motherhood in childhood: Facing the challenge of adolescent pregnancy.Paris: UNFPA;2013.
4. Ayele TA, Azale T, Alemu K, Abdissa Z, Mulat H, Fekadu A. Prevalence andAssociated Factors of Antenatal Depression among Women Attending Antenatal Care Service at Gondar University Hospital, Northwest Ethiopia.. PLoS ONE.2016;11(5):e0155125.
5. Gust DA, Gvetadze R, Furtado M, et al. Factors associated with psychologicaldistress among young women in Kisumu, Kenya. International Journal of Women's Health. 2017;9:255-264.
6. Cox JL. Psychiatric morbidity and pregnancy: A controlled study of 263 semi-rural Ugandan women. Br J Psychiatry.1979;134:401-405.
7. Abiodun OA, Adetoro OO, Ogunbode OO. Psychiatric morbidity in a pregnant population in Nigeria. Gen Hosp Psychiatry.1993;15:125-128.
8. Hartley M, Tomlinson M, Greco E, et al. Depressed mood in pregnancy: Prevalenceand correlates in two Cape Town peri-urban settlements. Reprod Health.2011;8(9):1-7.
9. Yator O, Mathai M, Van der SA, D. R, Kumar M. Risk factors for postpartum depression in women living with ADOLESCENT MOTHERS attending prevention of motherto-child transmission clinic at Kenyatta National Hospital, Nairobi. AIDS Care.2016;28(7):884-889.
10. Gelaye B, Rondon M, Araya R, Williams MA. Epidemiology of maternal depression,risk factors, and child outcomes in low-income and middle-income countries. The Lancet. Psychiatry.2016;10(3):973–982.
11. Magadhe B, Kimani V, Nicodimos S, Van der SA, M. K. Postpartum depression and infant feeding practices in a low income urban settlement in Nairobi-Kenya. BMC Research Notes. 2016;9:506.
12. Bass JK, Ryder RW, Lammers M, al..e. Post-partum depression in Kinshasa. Democratic Republic of Congo: Validation of a concept using a mixed-methods cross-culturalapproach. Trop Med Int Health.2008;13(12):1534–1542.

13. Lund C, Breen A, Flisher AJ, al..e. Poverty and common mental disorders in low and middle income countries: A systematic review. Soc Sci Med..2010;71:517–528.

14. Fisher J, Cabral de Mello M, Patel V, et al. Prevalence and determinants of common perinatal mental disorders in women in low- and lower middle- income countries: A systematic review. Bulletin of the World Health Organization.2012;90(2):139-149H.

15. Senturk V, Hanlon C, Medhin G, al e. Impact of perinatal somatic and common mental disorder symptoms on functioning in Ethiopian women: the P-MaMiE population-based cohort study. J Affect Disorder.2012;136:340–349.

16. Rahman A, Malik A, Sikander S, Roberts C, and Creed F. Cognitive behavior therapy-based intervention by community health workers for mothers with depression and their infants in rural Pakistan: a cluster-randomised controlled trial. The Lancet. 2008;372(9642):602-909.

17. Sawyer A, Ayers S, Smith H. Pre- and postnatal psychological wellbeing in Africa:A systematic review. J Affect Disorder. 2010;123(1-3):17-29.

18. Bolton P, Bass J, Theresa B, et al. Interventions for Depression Symptoms Among Adolescent Survivors of War and Displacement in Northern Uganda: ARandomized Controlled Trial. JAMA.2007;298(5):519-527.

19. Onu C, Ongeri L, Bukusi E, et al. Interpersonal psychotherapy for depression and posttraumatic stress disorder among ADOLESCENT MOTHERS-positive women in Kisumu, Kenya: study protocol for a randomized controlled trial. Trial.2016;17(1):1.

20. Verdeli H, Clougherty K, Bolton P, et al. Adapting group interpersonal psychotherapy fora developing country: experience in rural Uganda. World Psychiatry.2003;2(2):114–120.

21. Mendenhall E, De Silva MJ, Hanlon C, et al. Acceptability and feasibility of using non-specialist health workers to deliver mental health care: Stakeholder perceptions from the PRIME district sites in Ethiopia, India, Nepal, South Africa and Uganda. Social Scienceand Medicine. 2014;18:33-42.

22. Lewandowski RE, Bolton PA, Feighery A, et al. Local perceptions of the impact ofgroup interpersonal psychotherapy in rural Uganda. Global Mental Health.2016;3(e23).

23. Singla DR, Weobong B, Nadkarni A, et al. Improving the scalability of psychological treatments in developing countries: An evaluation of peer-led therapy quality assessment in Goa, India. Behaviour Research and Therapy.2014;60(100):53-59.

24. Meffert SM, Neylan TC, Chambers DA, Verdeli H. Novel implementation researchdesigns for scaling up global mental health care: overcoming translational challenges to address the world's leading cause of disability. International Journal of Mental Health Systems. 2016;10:19.

25. UNICEF. https://data.unicef.org/topic/adolescents/adolescent-demographics/.2016.

26. WHO. http://www.who.int/mediacentre/factsheets/fs364/en/.2014.

27. Neal SE, Chandra-Mouli V, Chou D. Adolescent first births in East Africa:disaggregating characteristics, trends and determinants. Reproductive Health.2015;12(13).

28. UNAIDS, UNFPA, WHO. Seen but not Heard: Very Young Adolescents Aged 10–14 Years. Geneva: UNAIDS;2004.

29. Lieberman K, Le HN, Perry DF. A systematic review of perinatal depressioninterventions for adolescent mothers. Journal of Adolescence.2014;37(8):1227-35.

30. Mbelenga E, Othieno C, Kumar M. Impact of maternal depression on stunting and poor cognitive development of children ages 4-6 in Kitui, Kenya. BMC Psychiatry. Under preparation.

31. Magadhe B, Kimani V, Nicodimos S, Van der SA, M. K. Effects of post-partumdepression on infant feeding practices and child malnutrition in an informal settlement based MCH clinic in Nairobi. BMC Research Notes.2016;9:506.

32. Huang KY, Nakigudde J, Kumar M, de-Graft AA. Parent Emotion Socialization and Young Children’s Social Emotion Development: Mechanism Testing with Families from Uganda, Kenya and Ghana; 2016.

33. Pradhan R, Wynter K, Fisher J. Factors associated with pregnancy among adolescentsin low-income and lower middle-income countries: a systematic review. J Epidemiol Community Health.2015;69(9):918-24.

34. Siegel RS, Brandon AR. Adolescents, pregnancy, and mental health. [Review].. Journal of Pediatric and Adolescent Gynecology.2014;27(3):138-150.

35. Atuyambe L, Mirembe F, Annika J, Kirumira EK, Faxelid E. Seeking safety andempathy: adolescent health seeking behavior during pregnancy and early motherhood in central Uganda. J Adolesc.2009;32(4):781-96.

36. Juma M, Alaii J, Bartholomew LK, Askew L, Van den Born B. Understanding orphan and non-orphan adolescents’ sexual risks in the context of poverty: a qualitative study inNyanza Province, Kenya. BMC International Health and Human Rights. 2013;13(32).

37. UNFPA. Motherhood in childhood: Facing the challenge of adolescent pregnancy.Paris: UNFPA;2013.

38. Kumar M. Child Behavioral Health issues in Kenya. Kampala, Uganda2016.

39. WHO. WHO. Mental Health Action Plan 2013-2020. 2013. 2015. Availableat: http://www.who.int/mental_health/action_plan_2013/en/,2015.

40. mhGAP.http://www.who.int/mental_health/mhgap/training_manuals/en/.

41. WHO. mhGAP Intervention Guide for mental, neurological and substance use disorders in non-specialized health settings. Geneva2010a.

42. WHO. Maternal Mental Health and Child Health and Development in Low andMiddle Income Countries. Geneva2008a.

43. WHO. Improving health systems and services for mental health. Geneva: WHO;2009a.

44. Rahman A, Fisher J, Bower P, et al. Interventions for common perinatal mental disordersin women in low- and middle-income countries: a systematic review and meta-analysis. Bulletin of the World Health Organization.2013:593-601.

45. Mendenhall E, De Silva MJ, Hanlon C, et al. Acceptability and feasibility of using non-specialist health workers to deliver mental health care: Stakeholder perceptions from the PRIME district sites in Ethiopia, India, Nepal, South Africa and Uganda. Social Scienceand Medicine. 2014;18:33-42.

46. Chiumento A, Hamdani SU, Khan MN, et al. Evaluating effectiveness and cost-effectiveness of a group psychological intervention using cognitive behaviouralstrategies for women with common mental disorders in conflict-affected rural Pakistan: Study protocall for randolised controlled trial. Trial.2017;18(190).

47. Osok J, Kigamwa P, Vander SA, Huang KY, Bauta B, Kumar M. Experiences ofdepression and interpersonal challenges of pregnant adolescents seeking prenatal health care. Vulnerable child and youth studies. 2016;Underpreparation.

48. Atuyambe L, Mirembe F, Johansson A, Kirumira EK, Faxelid E. Experiences of pregnant adolescents - voices from Wakiso district, Uganda. African Health Sciences. 2005;5(4):304-309.

49. Fisher J, Nguyen H, Mannava P, et al. Translation, cultural adaptation and field-testing of the Thinking Healthy Program for Vietnam. Globalization and Health.2014;10(37).

50. Rahman A. Challenges and opportunities in developing a psychological intervention for perinatal depression in rural Pakistan – a multi-method study. Archives of Women’s Mental Health.2007;10(5):211–219.

51. Rahman A, Malik A, Sikander S, Roberts C, and Creed F. Cognitive behaviour therapy-based intervention by community health workers for mothers with depression and their infants in rural Pakistan: a cluster-randomised controlled trial. The Lancet. 2008;372(9642):602-909.

52. WHO. Thinking Healthy Program Manual. 2015. Available at: http://www.who.int/mental_health/maternal-child/thinking_healthy/en/.

53. Bolton P, Bass J, Theresa B, et al. Interventions for Depression Symptoms Among Adolescent Survivors of War and Displacement in Northern Uganda: ARandomized Controlled Trial. JAMA.2007;298(5):519-527.

54. http://apps.who.int/iris/bitstream/10665/250219/1/WHO-MSD-MER-16.4-eng.pdf?ua=1.

55. Damschroder LJ, Lowery JC. Evaluation of a Large-scale weight managementprogram using the consolidated framework for implementation research (CFIR). Implementation Science.2013;8(51):2-17.

56. Proctor E, Silmere H, Raghavan R, et al. Outcomes for implementation research:conceptual distinctions, measurement challenges, and research agenda. Administration and policy in mental health.2011;38(2):65-76.

57. Huang KY, Nakigudde J, Calzada E, Boivin MJ, Ogedegbe G, Brotman LMI.Implementing an early childhood school-based mental health promotion intervention in low-resource Ugandan schools: study protocol for a cluster randomized controlled trial. Trial. 2014;15:471.

58. Maselli D, Lys JA, Schmid J. Improving Impacts of Research Partnerships. Swiss Commission for Research Partnerships with Developing Countries, KFPE.Switzerland: GEOGRAPHICA BERNENSIA, Berne; 2004.

59. Kirk MA, Kelley C, Yankey N, Birken SA, Abadie B, Damschroder L. A systematicreview of the use of the Consolidated Framework for Implementation Research. Implementation Science : IS.2015;11:72.

60. Orme B. Sample size issues for conjoint analysis studies. Sequim: Sawtooth Software Technical Paper; 1998

61. Johnson R, Orme B. Getting the most from CBC. Sequim: Sawtooth Software Research Paper Series, Sawtooth Software; 2003

62. Orme B. Getting started with conjoint analysis: Strategies for product design andpricing research. Second edition ed. Madison: Wisconsin: Research publishers LLM;2010.

63. Engle PL. Maternal mental health: program and policy implications. Am JClinNutr. 2009;89(96):3S–6S.

64. Alford S, Cheetham N, Hauser D. Science & Success in Developing Countries: Holistic Programs that Work to Prevent Teen Pregnancy, HIV & Sexually TransmittedInfections. Washington, DC: DCAdvocates for Youth; 2005.

65. Mishler E. Research Interviewing: Context and Narrative. Cambridge, MA:Harvard University Press;1986.

66. Riessman CK. Narrative Analysis. Newbury Park, CA: Sage;1993.

67. Charmaz K. Constructing Grounded Theory: A Practical Guide ThroughQualitative

Analysis. Thousand Oaks, CA: Sage Publications;2000.

68. Blumer H. Symbolic interactions: Perspective and method. Englewoods Cliffs, NJ: Prentice Hall.;1969.
69. Sawtooth Software Inc. The CBC system for choice-based conjointanalysis.
70. IBM, Corp. Released 2016. IBM SPSS Statistics for Windows, Version24.0.
71. Chorpita BF, Moffitt CE, Gray J. Psychometric properties of the Revised Child Anxiety and Depression Scale in a clinical sample. *Behav Res Ther*. 2005;43(3):309-322. doi:10.1016/j.brat.2004.02.004
72. Bradley AP, Longstaff ID. Sample size estimation using the receiver operating characteristic curve. In: *Proceedings of the 17th International Conference on Pattern Recognition, 2004. ICPR 2004.* IEEE; 2004:428-431 Vol.4. doi:10.1109/ICPR.2004.1333794
73. Metz CE. Basic principles of ROC analysis. *Semin Nucl Med*. 1978;8(4):283-298. doi:10.1016/S0001-2998(78)80014-2
74. Braun V, Clarke V. Using thematic analysis in psychology. *Qual Res Psychol*. 2006;3(2):77-101. doi:10.1191/1478088706qp063oa
75. van Ommeren M, Sharma B, Thapa S, et al. Preparing Instruments for Transcultural Research: Use of the Translation Monitoring Form with Nepali-Speaking Bhutanese Refugees. *Transcult Psychiatry*. 1999;36(3):285-301. doi:10.1177/136346159903600304
76. Labrique AB, Vasudevan L, Kochi E, Fabricant R, Mehl G. mHealth innovations as health system strengthening tools: 12 common applications and a visual framework. Global Health: Science and Practice. 2013;1:161-171.
77. Venkatesh V, Bala H. Technology Acceptance Model 3 and a Research Agenda on Interventions. Decision Sciences. 2008;39:273-315.
78. Huang KY, Nakigudde J, Ruhule D, et al. Transportability of an Evidence-based Early Childhood Intervention in a Low-Income African Country: Results of a ClusterRandomized Controlled Implemention Study. Prevention Science.2017;18:964-975.
79. https://www.nimh.nih.gov/news/science-news/2015/new-nimh-strategic-plan-aims-to-focus-accelerate-mental-health-research.shtml.
