# Supplementary material for: Group Interpersonal Psychotherapy for Depression in Perinatal Adolescents in Kenya: A Pilot Randomized Clinical Trial
Source: JAMA Netw Open. 2026 Jun 16;9(6):e2618255. doi: 10.1001/jamanetworkopen.2026.18255 (PMC13273492; doi:10.1001/jamanetworkopen.2026.18255)
Supplement: Supplement 2. — eMethods 1. Facilitator Recruitment and Training eMethods 2. Sample Size and Power eMethods 3. Primary Outcome Analysis eMethods 4. Effect Sizes eMethods 5. Hypothesis Testing and Significance Level eMethods 6. Analysis Population eMethods 7. Multiple Comparisons eMethods 8. Missing Data Handling eMethods 9. Subgroup and Sensitivity Analyses eMethods 10. Software and Code Availability eMethods 11. Secondary Outcomes eMethods 12. Subgroup Analysis by Baseline Depression Severity eTable 1. Description of Intervention and Comparison Arms eTable 2. Summary of Study Measures, Psychometric Properties, and Scoring eTable 3. Baseline Characteristics by Completeness of PHQ-9 Data eTable 4. Additional Baseline Characteristics by Intervention Arm eTable 5. Attendance Metrics by Intervention Arm eTable 6. Adjusted Differences in PHQ-9 Scores by Intervention Group and Baseline Severity eFigure 1. Session by Session Attendance Rates eFigure 2. Mean FFC Over Time by Intervention eFigure 3. Mean WHODAS Over Time by Intervention eFigure 4. Mean WHO Well-Being Over Time by Intervention eFigure 5. Mean PTSD Over Time by Intervention eFigure 6. Mean Multidimensional Scale of Social Support Over Time by Intervention eFigure 7. Mean HITS Over Time by Intervention eFigure 8. Mean General Self-Efficacy Over Time by Intervention eFigure 9. Mean CORE-10 Over Time by Intervention eFigure 10. Mean COPE Over Time by Intervention eFigure 11. Average Depression Scores by Session and Intervention Arm eFigure 12. Average Depression Scores by Session and Individual eReferences. [file jamanetwopen-e2618255-s002.pdf]

Supplementary Online Content

Kumar M, Tele A, Nyongesa V, et al. Group interpersonal psychotherapy for depression in perinatal adolescents in Kenya: a randomized clinical trial. *JAMA Netw Open.* 2026;9(6):e2618255. doi:10.1001/jamanetworkopen.2026.18255

eMethods 1. Facilitator Recruitment and Training

eMethods 2. Sample Size and Power

eMethods 3. Primary Outcome Analysis

eMethods 4. Effect Sizes

eMethods 5. Hypothesis Testing and Significance Level

eMethods 6. Analysis Population

eMethods 7. Multiple Comparisons

eMethods 8. Missing Data Handling

eMethods 9. Subgroup and Sensitivity Analyses

eMethods 10. Software and Code Availability

eMethods 11. Secondary Outcomes

eMethods 12. Subgroup Analysis by Baseline Depression Severity

eTable 1. Description of Intervention and Comparison Arms

eTable 2. Summary of Study Measures, Psychometric Properties, and Scoring

eTable 3. Baseline Characteristics by Completeness of PHQ-9 Data

eTable 4. Additional Baseline Characteristics by Intervention Arm

eTable 5. Attendance Metrics by Intervention Arm

eTable 6. Adjusted Differences in PHQ-9 Scores by Intervention Group and Baseline Severity

eFigure 1. Session by Session Attendance Rates

eFigure 2. Mean FFC Over Time by Intervention

eFigure 3. Mean WHODAS Over Time by Intervention

eFigure 4. Mean WHO Well-Being Over Time by Intervention

eFigure 5. Mean PTSD Over Time by Intervention

© 2026 Kumar M et al. *JAMA Network Open*.

eFigure 6. Mean Multidimensional Scale of Social Support Over Time by Intervention

eFigure 7. Mean HITS Over Time by Intervention

eFigure 8. Mean General Self-Efficacy Over Time by Intervention

eFigure 9. Mean CORE-10 Over Time by Intervention

eFigure 10. Mean COPE Over Time by Intervention

eFigure 11. Average Depression Scores by Session and Intervention Arm

eFigure 12. Average Depression Scores by Session and Individual

eReferences.

This supplementary material has been provided by the authors to give readers additional information about their work.

© 2026 Kumar M et al. *JAMA Network Open*.

eMethods 1. Facilitator Recruitment and Training

CHPs from the two participating health centers were recruited as facilitators based on their experience with adolescents, communication skills, and availability for weekly sessions. Seven CHPs were selected and trained using a train-the-trainer model. Facilitators completed a five-day intensive training workshop, primarily online with in-person field sessions, covering IPT principles, group facilitation, manual delivery, and safety procedures for participant distress. Additional training based on WHO mhGAP guidelines included online instruction and face-to-face competency assessments to enhance clinical skills further.

Facilitator Supervision

Facilitators received ongoing supervision during the intervention to maintain fidelity to the IPT model and support skill development. Supervision involved on-site observation, monthly refresher training, and structured case discussions, all provided by the study's clinical psychologists. Facilitators and supervisors were not blinded to intervention arms.

eMethods 2. Sample Size and Power

This was a pilot implementation-effectiveness study rather than a fully powered randomized controlled trial. As specified in the protocol, the primary purpose was to test preliminary effectiveness, estimate effect sizes for future studies, and assess feasibility outcomes (attendance, retention, fidelity). The sample size (N = 122 at baseline across three arms: Treatment as Usual [TAU] = 44, Mini-IPT = 38, Full-IPT = 40) was therefore not calculated to achieve statistical power for confirmatory hypothesis testing. Instead, the sample size was selected to: (1) estimate key feasibility outcomes with a margin of error of approximately ±10-15% at 95% confidence; (2) generate preliminary effect size estimates (Cohen d with 95% confidence intervals) to inform sample size calculations for a future fully powered randomized controlled trial; and (3) accommodate practical constraints including study budget and timeline.

A post-hoc sensitivity analysis was conducted to characterize the precision of the study for preliminary effectiveness comparisons. Assuming a two-sided significance level of $\alpha = 0.05$ and a sample size of approximately 90 participants with outcome data at 6 months, the study provides 80% power to detect a moderate effect size (d = 0.62-0.68) for pairwise comparisons. However, because this is a pilot study, we emphasize that all hypothesis tests are exploratory and hypothesis-generating. The study was not powered to detect small effect sizes or to definitively establish superiority of one arm over another. Results should be interpreted as preliminary.

eMethods 3. Primary Outcome Analysis

To examine changes in PHQ-9 scores over time by intervention arm, we fitted a linear mixed-effects model (multi-level model) with repeated measures nested within participants. The model included the following predictors:

- Time (categorical: baseline, within 1 week post intervention, 6 months)
- Intervention arm (categorical: TAU [reference], Mini-IPT, Full-IPT)
- Time × arm interaction (to test whether the trajectory of PHQ-9 scores differed between arms over time)

No mathematical transformations were applied to these variables. No additional covariates were included in the primary model due to the limited sample size (n = 122 at baseline, n = 91 at 6 months), which restricts model complexity to avoid overfitting. The scientific rationale for including time, arm, and their interaction is based on the primary research question of whether either IPT protocol reduces depressive symptoms compared to TAU, and whether the Mini-IPT protocol produces similar effects to the full-length protocol. Baseline PHQ-9 score was examined as a potential covariate in sensitivity analyses and did not substantively change the results.

The model was estimated using conditional likelihood estimation (restricted maximum likelihood [REML]), which accounts for missing data under the missing at random assumption. Random intercepts were included for each participant to account for within-subject correlation of repeated measures. We avoid the term "fixed effects" to describe predictors; instead, we refer to "model predictors" or "variables." Pairwise comparisons between intervention arms at each time point were estimated using post-estimation contrasts (Stata's margins command). Estimated marginal means (EMMs) were used to estimate group differences at post-intervention and follow-up.

eMethods 4. Effect Sizes

© 2026 Kumar M et al. *JAMA Network Open*.

Cohen d effect sizes were calculated as the difference in estimated marginal means divided by the pooled baseline standard deviation, with 95% confidence intervals for each comparison. Effect size calculations were performed using R version 4.2.0 with the effsize package.

eMethods 5. Hypothesis Testing and Significance Level

All hypothesis tests were two-sided with a significance level (alpha) of 0.05. For the primary outcome (change in PHQ-9 score over time), we tested the following null hypotheses:

1. The time × Mini-IPT interaction coefficient equals zero (no difference in trajectory between Mini-IPT and TAU).
2. The time × Full-IPT interaction coefficient equals zero (no difference in trajectory between Full-IPT and TAU).
3. The difference between Mini-IPT and Full-IPT slopes equals zero (no difference between the two active arms).

eMethods 6. Analysis Population

All analyses followed the intention-to-treat (ITT) principle, with participants analyzed according to their original arm assignment regardless of attendance or protocol adherence.

eMethods 7. Multiple Comparisons

Secondary outcomes are interpreted as exploratory and hypothesis-generating. No corrections for multiple testing were applied given the pilot nature of the study. P-values for secondary outcomes should be interpreted with caution, and findings require confirmation in future definitive trials.

eMethods 8. Missing Data Handling

Missing PHQ-9 scores occurred when participants missed a scheduled session or withdrew from the study. The proportion of missing data at each time point by arm is reported in the Results section. Reasons for missingness included session non-attendance and study withdrawal.

The linear mixed-effects model was estimated using conditional likelihood estimation (REML), which uses all available data and provides unbiased estimates under the missing at random (MAR) assumption without the need for explicit imputation. As a sensitivity analysis, we also conducted complete-case analysis (excluding participants with any missing outcome data). Last observation carried forward (LOCF) was not used.

A supplementary table comparing baseline characteristics between participants with complete versus incomplete outcome data is provided (eTable 3). Little's test for missing completely at random (MCAR) was non-significant ($\chi^2 = 12.34$, df = 15, p = 0.65), supporting the MAR assumption.

eMethods 9. Subgroup and Sensitivity Analyses

No subgroup analyses were planned or conducted due to the limited sample size. Sensitivity analyses were performed by including baseline PHQ-9 score as a covariate in the primary model; these did not substantively change the direction, magnitude, or statistical significance of the results.

eMethods 10. Software and Code Availability

All multi-level model analyses were conducted using Stata version 18.0 [1] with the mixed command for multi-level models and margins for pairwise comparisons. Effect sizes (Cohen d) were calculated using R version 4.2.0 [2] with the effsize package. The full analysis code for both Stata and R is provided in Supplementary Code S1 (online supplement). The Statistical Analysis Plan (SAP) is provided in Supplementary SAP S1.

eMethods 11. Secondary Outcomes

Secondary outcomes showed variable patterns across domains. At post-intervention, psychological distress as measured by the CORE-10 decreased significantly in the Full-IPT-G arm compared with TAU (β = -3.308, 95% CI -6.167 to -0.449; P = .02), but the reduction for Mini-IPT-G compared with TAU was not statistically significant (β = -1.433, 95% CI -4.272 to 1.406; P = .32). Conversely, posttraumatic stress symptoms (NSESSS-PTSD) showed a

© 2026 Kumar M et al. *JAMA Network Open*.

significant reduction for Mini-IPT-G compared with TAU (β = 5.041, 95% CI 0.948 to 9.133; P = .02), while the difference for Full-IPT-G compared with TAU did not reach statistical significance (β = 2.642, 95% CI -1.479 to 6.763; P = .21). Subjective mental well-being (WHO-5) showed a non-significant trend favoring Full-IPT-G over TAU (β = 11.050, 95% CI -0.738 to 22.838; P = .07). No other secondary outcomes at post-intervention, including intimate partner violence (HITS), coping strategies (Brief COPE), self-efficacy (GSE), social support (MDSPSS), or functional disability (WHODAS), demonstrated statistically significant between-group differences (all P ≥ .14).

At 6-month follow-up, subjective mental well-being (WHO-5) showed significant improvement for Mini-IPT-G compared with TAU (β = 12.483, 95% CI 0.788 to 24.179; P = .04). A non-significant trend was observed for intimate partner violence (HITS) favoring Full-IPT-G over TAU (β = 1.329, 95% CI -0.226 to 2.884; P = .09). No other secondary outcomes demonstrated statistically significant between-group differences at follow-up, including psychological distress (CORE-10), posttraumatic stress symptoms (NSESSS-PTSD), coping strategies (Brief COPE), self-efficacy (GSE), social support (MDSPSS), or functional disability (WHODAS) (all P ≥ .11) (eFigures 11 and 12).

eMethods 12. Subgroup Analysis by Baseline Depression Severity

To explore whether treatment effects differed by baseline depression severity, participants were stratified into mild-moderate (PHQ-9 scores 5-14) and moderately severe (PHQ-9 scores 15-19) groups. A mixed-effects model including a three-way interaction between time, intervention group, and baseline severity was fitted. Baseline severity was a significant predictor of PHQ-9 scores, and there was evidence that changes over time differed by severity level.

Participants with mild to moderate baseline symptoms who received Full IPT-G experienced greater reductions in PHQ-9 scores compared with enhanced TAU within 1 week post intervention (adjusted mean difference, -2.90; 95% CI, -4.91 to -0.90; P = .005) and 6 months (-4.38; 95% CI, -7.74 to -1.01; P = .011). Mini IPT-G did not differ from TAU at either time point. Among participants with moderately severe baseline symptoms, Full IPT-G was associated with greater reductions in PHQ-9 scores at 1 week post intervention (-3.52; 95% CI, -6.36 to -0.69; P = .015), although this effect was not sustained at 6 months (-1.14; 95% CI, -4.06 to 1.78; P = .44). Mini IPT-G showed a borderline effect at 1 week post intervention (-1.98; 95% CI, -3.96 to 0.00; P = .051) and a modest reduction at 6 months (-2.83; 95% CI, -5.51 to -0.15; P = .039) (eTable 6).

© 2026 Kumar M et al. *JAMA Network Open*.

eTable 1. Description of Intervention and Comparison Arms

Domain	TAU (Enhanced Usual Care)	Mini IPT-G	Full IPT-G
**Purpose / Rationale**	Ethical enhanced usual care to control for nonspecific study contact; psychoeducation to support symptom recognition, help-seeking, and stigma reduction	Brief, front-loaded adaptation of IPT-G designed to capture early clinical gains while reducing participant burden and maximizing scalability	Standard evidence-based IPT-G dose to allow phased skill acquisition, deeper interpersonal work, and potentially more durable clinical change
**WHO mhGAP integration**	Health facility nurses, clinical officers, and nursing officers trained in WHO mhGAP essential care principles and depression module, including referral pathways	Same mhGAP training as TAU for facility staff. For CHPs delivering IPT-G: on-site supervision by a psychologist plus weekly virtual group supervision and debriefs with the full clinical team and county officials overseeing adolescent health, mental health, and GBV/social protection. Supervision entails IPT-G facilitators presenting experiences and observations during the weekly sessions, focusing on their achievements, challenges, areas of improvement, and setting therapy goals for subsequent sessions	Same mhGAP training and supervision structure as Mini IPT-G
**Core Components**	Self-directed psychoeducational pamphlets; information on depression, help-seeking, and referral; follow-up if requested; research assistant telephone check-ins at 3 and 6 months	One individual pre-group engagement session; structured group IPT-G focused on priority interpersonal problem areas (eg, role transitions, disputes), with emphasis on pregnancy, transition to motherhood, and future planning identified by the group	One individual pre-group engagement session; full phased IPT-G protocol emphasizing peer support, interpersonal problem-solving, emotional processing, and future planning (eg, motherhood, education, livelihoods)
**Implementation Strategies**	Multiple sources of information on help-seeking and referral; clear contact details provided;	*Task-sharing and task-shifting model*: nurses screen and introduce psychotherapy; CHPs recruit participants and deliver IPT-G. County and facility focal persons	Same *task-sharing/task-shifting* model as Mini IPT-G: mhGAP-trained nurses screen and introduce psychotherapy; CHPs deliver IPT-G and support follow-up. County and facility focal persons engage groups for linkage

© 2026 Kumar M et al. *JAMA Network Open*.

	warning signs highlighted	periodically engage groups to link participants to essential services (eg, diapers, sanitary pads, food banks, transport support, medical management, birth spacing). Facility staff provide *stigma-reduction messages* related to adolescent pregnancy and mental health. *Engagement strategies* used to promote disclosure and peer support.<br><br>Paired facilitation with experienced CHPs during therapy delivery; onsite supervisors use session checklists to provide real-time feedback.	to social and health supports. Nurses and CHPs provide *stigma-reduction messages* on adolescent pregnancy and mental health. Similar *engagement strategies* used to strengthen peer support and participation.<br><br>Paired facilitation with experienced CHPs; session-level fidelity checklists with real-time feedback.
**Dose & Duration**	Pamphlets at baseline; telephone follow-up at 3 and 6 months	1 individual session plus 4 weekly 90-minute group sessions over approximately 5 weeks (≈7.5 hours total)	1 individual session plus 8 weekly 90-minute group sessions over approximately 9 weeks (≈13.5 hours total)
**Mode of Delivery**	Individual written materials and telephone follow-up	Face-to-face delivery: individual pre-group session followed by closed, in-person therapy groups	Face-to-face delivery: individual pre-group session followed by closed, in-person therapy groups
**Providers**	Routine facility staff for usual care; research assistants conduct follow-up calls	mhGAP-trained nurses conduct screening using the EPDS; selected CHPs recruit participants; designated CHPs deliver IPT-G under clinical psychologist supervision, with psychiatrist oversight for mhGAP-guided medical management when indicated. CHPs received pre- and post-training assessments; training delivered in-person and virtually.	mhGAP-trained nurses conduct screening using the EPDS; CHPs deliver IPT-G under clinical psychologist supervision. CHPs received pre- and post-training assessments; training delivered in-person and virtually.
**Safety Planning & Advanced Referrals**	Nurses trained in mhGAP depression module, including assessment and	Integrated safety protocol with regular consultation among nurses, PI, psychologists, and	Same as Mini IPT-G: integrated safety protocol with regular review by nurses, PI, psychologists, and psychiatrists; stepped-up care and

© 2026 Kumar M et al. *JAMA Network Open*.

	management of suicide and self-harm; clear internal and external referral pathways and key study contacts provided	psychiatrists for safety planning and stepped-up care when symptoms worsen or when substance use or suicidal risk is identified. Participants remained in groups unless withdrawal was requested or imminent risk was identified	referral for symptom worsening, substance use, self-harm, or suicidal risk; continued group participation unless withdrawal is requested or risk is imminent
**Tailoring**	None beyond standardization; pamphlets vetted by the Kenyan Ministry of Health and aligned with WHO mhGAP	Interpersonal inventory used to identify and prioritize a primary problem area; examples and exercises adapted to adolescents' social context. The depression rating on the PHQ-9, mood rating at the beginning of each session, and life events between sessions were also considered when setting goals for each of the 4 sessions.	Interpersonal inventory and IPT-G timeline used to guide phased work on primary problem area(s), with tailoring to developmental and social context. The depression rating on the PHQ-9, mood rating at the beginning of each session, and life events between sessions were also considered when setting goals for each of the 8 sessions.
**Fidelity Monitoring**	Not formally assessed	The trained IPT-G facilitators were subjected to WHO IPT Knowledge test to assess their level of retention (cut-off for qualification set at 70% performance). WHO IPT-G fidelity checklists; monthly refresher trainings; multilevel supervision (psychologists, psychiatrists, facility, and county stakeholders). Session-level checklists used onsite; real-time feedback; virtual supervision by master facilitators	The trained IPT-G facilitators were subjected to WHO IPT Knowledge test to assess their level of retention (cut-off for qualification set at 70% performance). WHO IPT-G fidelity checklists; monthly refreshers; ongoing supervision by clinical psychologists and psychiatrists. Session-level checklists used onsite; real-time feedback; virtual supervision by master facilitators
**Implementation Intensity**	Low	Moderate	High

eTable 2. Summary of Study Measures, Psychometric Properties, and Scoring

Outcome Measure	Domain	Number of Items	Scoring	Reliability (Cronbach's α)	Outcome Type	Source of outcome measure
Edinburgh Postnatal Depression Scale (EPDS)	Depression screening	10	0–3 per item; total 0–30; >10 indicates eligibility	0.78	Screening	[3]
Patient Health Questionnaire-9 (PHQ-9)	Depression severity	9	0–3 per item; total 0–27; higher = worse	0.86–0.89	Primary	[4,5]
Functioning Items	Functional Limitations	3	Range 0-3 : Higher scores = more frequent disruption of daily life, work/school, or social relationships caused by the problems. Lower scores = little or no disruption.	Good (varies)	Primary	[6,7]
Family functioning scale	Relationships within family	12	Strongly Agree - 4, Agree - 3, Disagree - 2, Strongly disagree - 1. The odd items are reversed scored (5 - value).		Primary	[8]
Hurt, Insult, Threaten, Scream (HITS)	Intimate partner violence	4	Range 4-20 : Higher scores = existence of intimate partner violence. Lower scores = little or no disruption.	~0.80	Secondary	[9]
WHO-5 Well-Being Index	Subjective mental well-being	5	0–5 per item; total 0–25; <13 = poor well-being	0.84	Secondary	[10]
General Self-Efficacy Scale (GSE)	Self-efficacy	10	1–4 per item; total 10–40; higher = greater efficacy	0.76–0.90	Secondary	[11,12]
WHODAS 2.0 (Short Version)	Functional disability	12	0–4 per item; transformed 0–100; higher = worse functioning	0.84–0.92	Secondary	[13]
CORE-10	Psychological distress	10	0–4 per item; higher = greater distress	Reliable change index 6	Secondary	[14]
Brief COPE Inventory	Coping strategies	28	1–4 per item; higher = greater use of coping strategy	0.60–0.90	Secondary	[15]

© 2026 Kumar M et al. *JAMA Network Open*.

Multidimensional Scale of Perceived Social Support (MSPSS)	Support	12	1-7 per item: Total Scale: Sum across all 12 items, then divide by 12	Cronbach's α typically > 0.85 across samples	Second ary	[16]
Severity of Posttraumatic Stress Symptoms – Adult" (NSESSS-PTSD)	PTSD	9	0-4; Summation (0-36)	Cronbach's α ≈ 0.90–0.94 in clinical and general population samples	Second ary	[17]

© 2026 Kumar M et al. *JAMA Network Open*.

eTable 3. Baseline Characteristics by Completeness of PHQ-9 Data

Characteristic	Complete data (n = 85)	Incomplete data (n = 37)	Test statistic	P-value
**Primary outcome**				
Baseline PHQ-9, mean (SD)	11.76 (5.03)	11.32 (5.84)	$t(120) = 0.42$	0.67
**Intervention arm, n (%)**			$\chi^2(2) = 5.87$	0.053
TAU	29 (34.1)	15 (40.5)		
Mini-IPT	32 (37.6)	6 (16.2)		
Full-IPT	24 (28.2)	16 (43.2)		
**Socioeconomic characteristics, n (%)**				
Marital status			$\chi^2(1) = 0.08$	0.78
Single	67 (78.8)	30 (81.1)		
Married/With partner	18 (21.2)	7 (18.9)		
Education			$\chi^2(1) = 0.07$	0.79
Primary school	21 (24.7)	10 (27.0)		
Secondary/High school	64 (75.3)	27 (73.0)		
Monthly income			$\chi^2(2) = 3.31$	0.19
<KSh 4,999/=	42 (49.4)	22 (59.5)		
KSh 5,000–9,999/=	28 (32.9)	13 (35.1)		
KSh 10,000 and above	15 (17.6)	2 (5.4)		
Persons living with			$\chi^2(2) = 2.79$	0.25
Parents	46 (54.1)	15 (40.5)		
Spouse/Partner	17 (20.0)	7 (18.9)		
Others	22 (25.9)	15 (40.5)		
Currently on medication			$\chi^2(1) = 2.16$	0.14
No	77 (90.6)	30 (81.1)		
Yes	8 (9.4)	7 (18.9)		
Gestational age at first ANC visit			$\chi^2(2) = 1.80$	0.41
< 12 weeks	24 (28.2)	15 (40.5)		
12-28 weeks	58 (68.2)	21 (56.8)		
> 28 weeks	3 (3.5)	1 (2.7)		

© 2026 Kumar M et al. *JAMA Network Open*.

Unplanned pregnancy			$\chi^2(1) = 1.35$	0.25
No	34 (40.0)	19 (51.4)		
Yes	51 (60.0)	18 (48.6)		
Presence of social support			$\chi^2(1) = 0.07$	0.79
No	10 (11.8)	5 (13.5)		
Yes	75 (88.2)	32 (86.5)		
Family history of mental illness			$\chi^2(1) = 0.09$	0.76
No	73 (85.9)	31 (83.8)		
Yes	12 (14.1)	6 (16.2)		
Intimate partner violence in pregnancy			$\chi^2(1) = 1.27$	0.26
No	66 (77.6)	32 (86.5)		
Yes	19 (22.4)	5 (13.5)		
Live with problem drinker/substance user			$\chi^2(1) = 0.27$	0.60
No	58 (68.2)	27 (73.0)		
Yes	27 (31.8)	10 (27.0)		
Ever consumed alcohol			$\chi^2(1) = 1.45$	0.23
No	60 (70.6)	22 (59.5)		
Yes	25 (29.4)	15 (40.5)		
Pressured into alcohol/substances			$\chi^2(1) = 0.58$	0.45
No	61 (71.8)	24 (64.9)		
Yes	24 (28.2)	13 (35.1)		

Note: Complete data defined as having PHQ-9 scores at both post-intervention (within 1 week post intervention) and follow-up (6 months). Incomplete data includes participants missing at either time point. All p-values are from chi-square tests for categorical variables and t-tests for continuous variables.

© 2026 Kumar M et al. *JAMA Network Open*.

eTable 4. Additional Baseline Characteristics by Intervention Arm

Measure	Full IPT (n = 40)	Mini IPT (n = 38)	TAU (n = 44)
	Mean (SD), Median [Min–Max; IQR]	Mean (SD), Median [Min–Max; IQR]	Mean (SD), Median [Min–Max; IQR]
**Age (Years)**	17.05 (1.01), 17 [13–18; 17–18]	17.11 (0.95), 17 [14–18; 17–18]	17.21 (0.77), 17 [16–18; 17–18]
**Depression (PHQ-9)**	12.38 (5.90), 12 [4–25; 7.5–16]	11.58 (4.93), 11 [3–21; 8–15]	11.00 (4.97), 10 [3–21; 7.5–15]
**Functioning (Functional Limitations)**	2.52 (0.70), 2.54 [1.08–3.58; 2.04–3.08]	2.48 (0.71), 2.29 [1.25–3.92; 2.00–2.92]	2.47 (0.74), 2.42 [1.00–4.00; 2.00–2.88]
**PTSD Severity (NSESSS-PTSD)**	9.60 (9.95), 7 [0–28; 0–17.5]	10.24 (11.42), 3.5 [0–32; 0–21]	8.91 (9.33), 6.5 [0–32; 0–14.5]
**Intimate Partner Violence (HITS)**	7.15 (3.59), 5 [5–23; 5–9]	7.47 (4.55), 5 [5–22; 5–8]	7.05 (3.68), 5 [5–18; 5–7]
**Psychological Distress (CORE-10)**	16.65 (6.30), 16 [5–32; 12–20.5]	16.37 (5.81), 16 [4–27; 13–21]	15.52 (8.07), 14 [1–32; 10–22.5]
**Coping (Brief COPE)**	14.05 (4.01), 14 [7–24; 11–16.5]	15.66 (3.78), 17 [8–21; 13–18]	16.07 (4.43), 16.5 [6–24; 13–19]
**Self-Efficacy (GSE)**	25.20 (8.56), 23 [12–39; 18.5–33]	26.16 (8.39), 28.5 [10–40; 19–33]	27.41 (8.32), 27.5 [10–40; 21–35]
**Social Support (MSPSS)**	4.06 (1.20), 4.08 [1.17–6.00; 3.25–4.83]	3.95 (1.24), 3.92 [1.92–6.08; 2.83–4.92]	4.27 (1.25), 4.17 [1.58–7.00; 3.25–5.33]
**Functional Disability (WHODAS 2.0)**	26.67 (17.82), 21.88 [0–72.92; 15.63–40.63]	23.14 (17.32), 18.75 [0–62.50; 12.5–33.33]	23.82 (16.78), 19.79 [2.08–62.50; 11.46–35.42]
**Well-Being (WHO-5)**	28.60 (16.47), 26 [0–72; 20–36]	31.68 (22.39), 26 [0–84; 20–40]	31.64 (22.39), 22 [4–84; 20–46]

Note.
Full IPT-G = Full Interpersonal Psychotherapy; Mini IPT-G = abbreviated IPT; TAU = Treatment As Usual; PHQ-9 = Patient Health Questionnaire-9; NSESSS-PTSD = National Stressful Events Survey PTSD Short Scale; HITS = Hurt, Insult, Threaten, Scream; CORE-10 = Clinical Outcomes in Routine Evaluation-10; GSE = General Self-Efficacy Scale; MSPSS = Multidimensional Scale of Perceived Social Support; WHODAS 2.0 = WHO Disability Assessment Schedule; WHO-5 = WHO-5 Well-Being Index.

© 2026 Kumar M et al. *JAMA Network Open*.

eTable 5. Attendance Metrics by Intervention Arm

Intervention Arm	N	Mean (SD)	Median [Q1, Q3]	Range (Min–Max)
Full-IPT-G	36	6.5 (2.24)	8 [6.75, 8]	1–8
Mini-IPT-G	36	3.6 (0.84)	4 [4, 4]	1–4

Note: Full-IPT-G had 8 scheduled sessions; Mini-IPT-G had 4 scheduled sessions.

© 2026 Kumar M et al. *JAMA Network Open*.

eTable 6. Adjusted Differences in PHQ-9 Scores by Intervention Group and Baseline Severity

Baseline Severity	Time Point	Comparison	Adjusted Mean Difference	95% CI	P value
**Mild–Moderate (5–14)**	1 Month	Mini IPT vs TAU	-0.87	-2.83 to 1.09	.38
		Full IPT vs TAU	**-2.90**	-4.91 to -0.90	**.005**
	6 Months	Mini IPT vs TAU	0.05	-3.31 to 3.42	.98
		Full IPT vs TAU	**-4.38**	-7.74 to -1.01	**.011**
**Moderately Severe (15–19)**	1 Month	Mini IPT vs TAU	-1.98	-3.96 to 0.00	.051
		Full IPT vs TAU	**-3.52**	-6.36 to -0.69	**.015**
	6 Months	Mini IPT vs TAU	-2.83	-5.51 to -0.15	.039
		Full IPT vs TAU	-1.14	-4.06 to 1.78	.44

© 2026 Kumar M et al. *JAMA Network Open*.

eFigure 1. Session by Session Attendance Rates

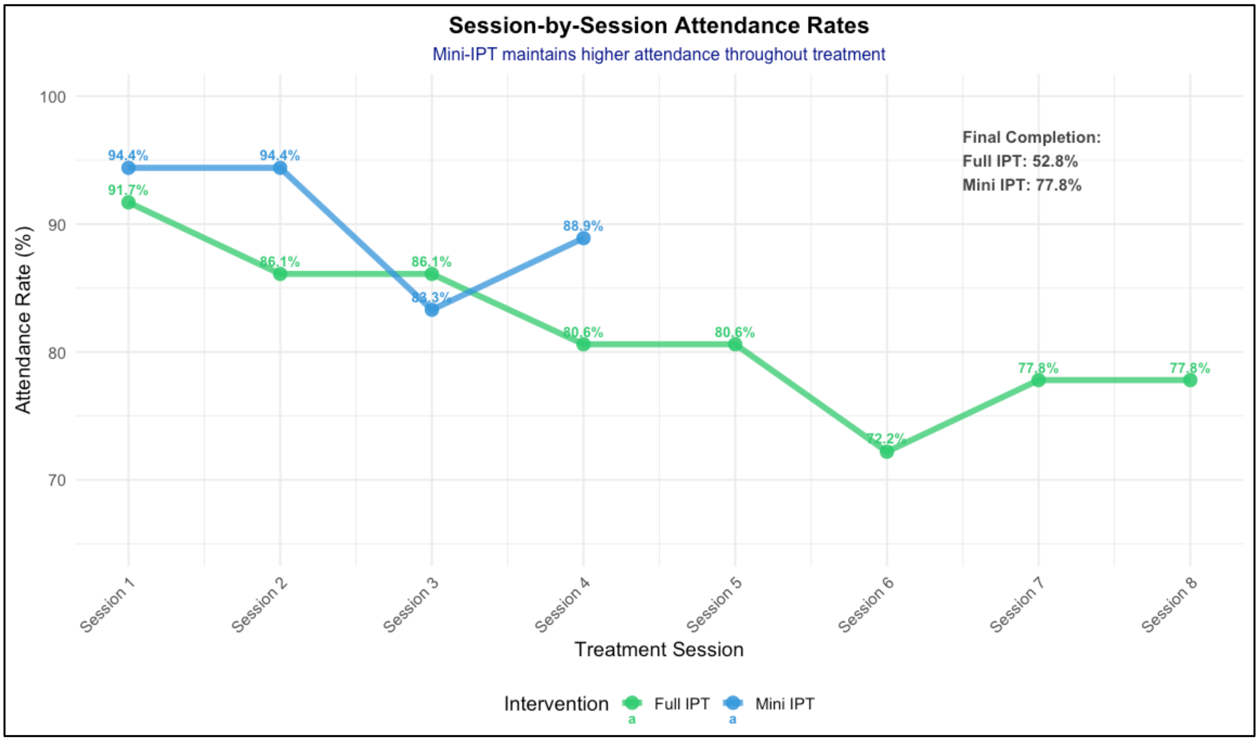

© 2026 Kumar M et al. *JAMA Network Open*.

eFigure 2. Mean FFC Over Time by Intervention

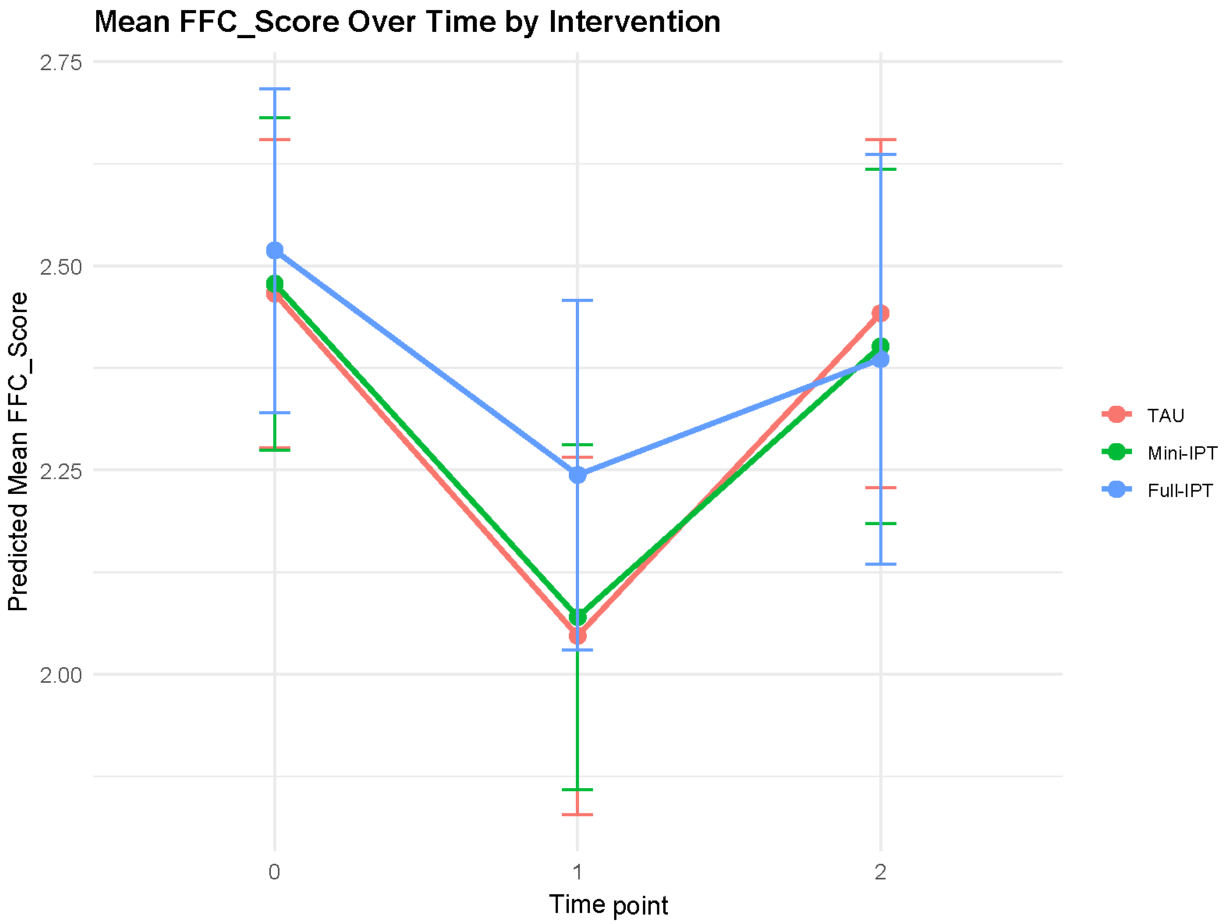

© 2026 Kumar M et al. *JAMA Network Open*.

eFigure 3. Mean WHODAS Over Time by Intervention

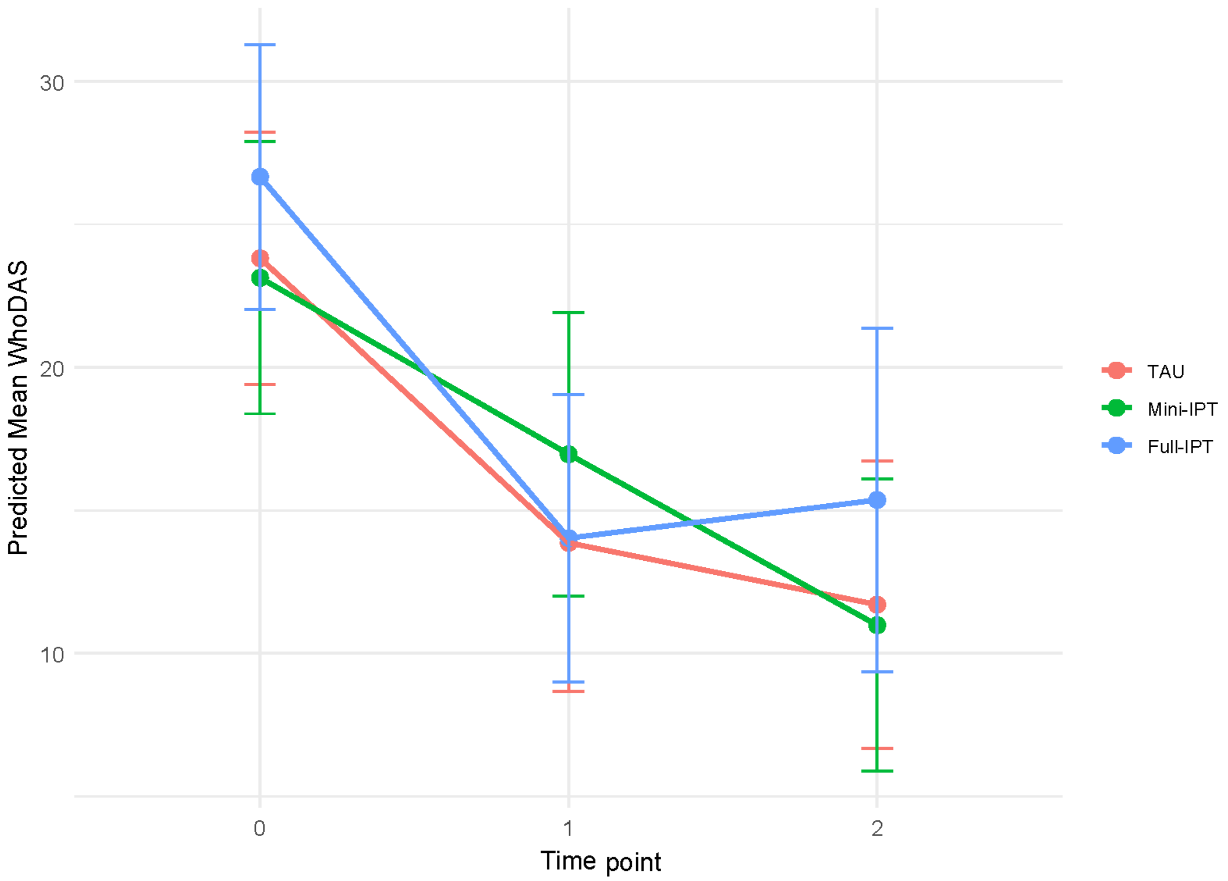

© 2026 Kumar M et al. *JAMA Network Open*.

eFigure 4. Mean WHO Well-Being Over Time by Intervention

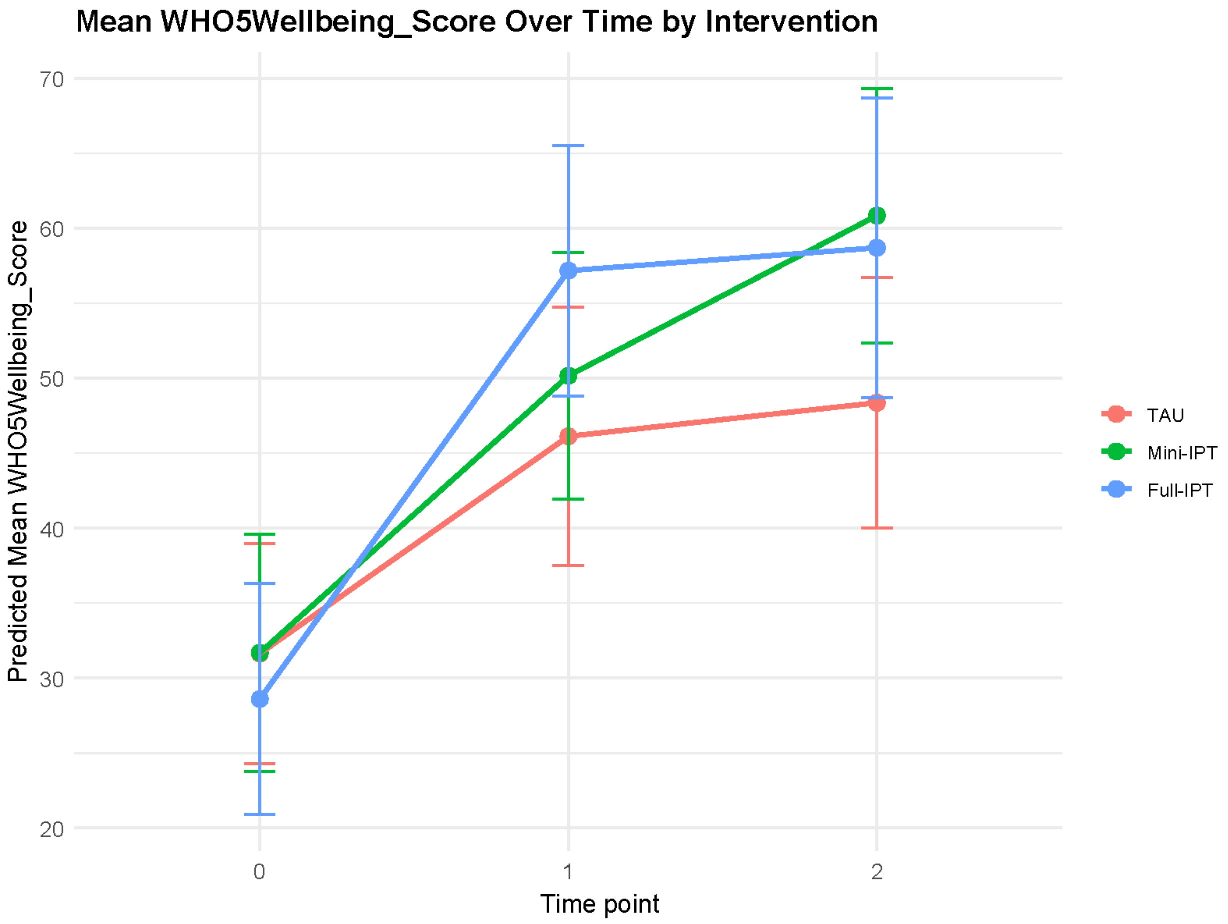

© 2026 Kumar M et al. *JAMA Network Open*.

eFigure 5. Mean PTSD Over Time by Intervention

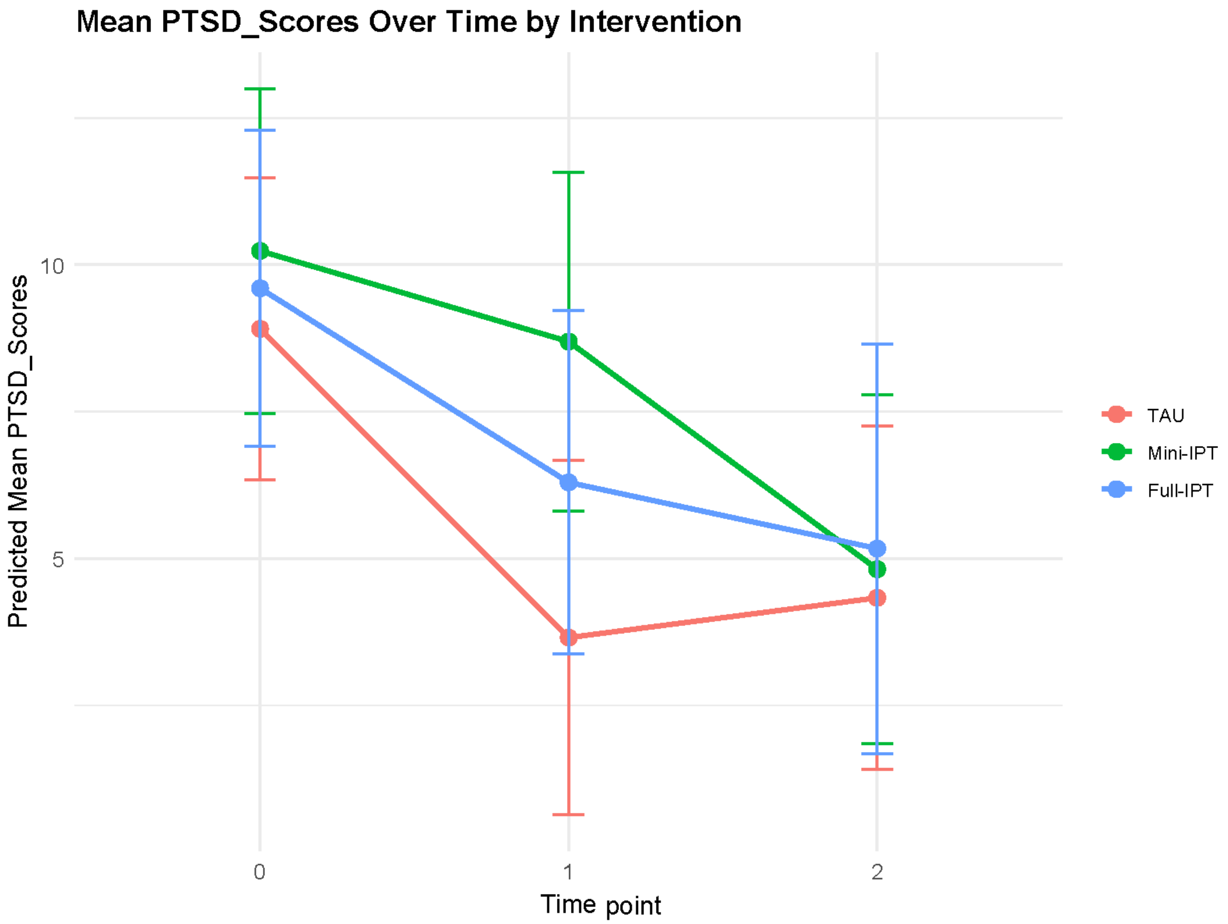

© 2026 Kumar M et al. *JAMA Network Open*.

eFigure 6. Mean Multidimensional Scale of Social Support Over Time by Intervention

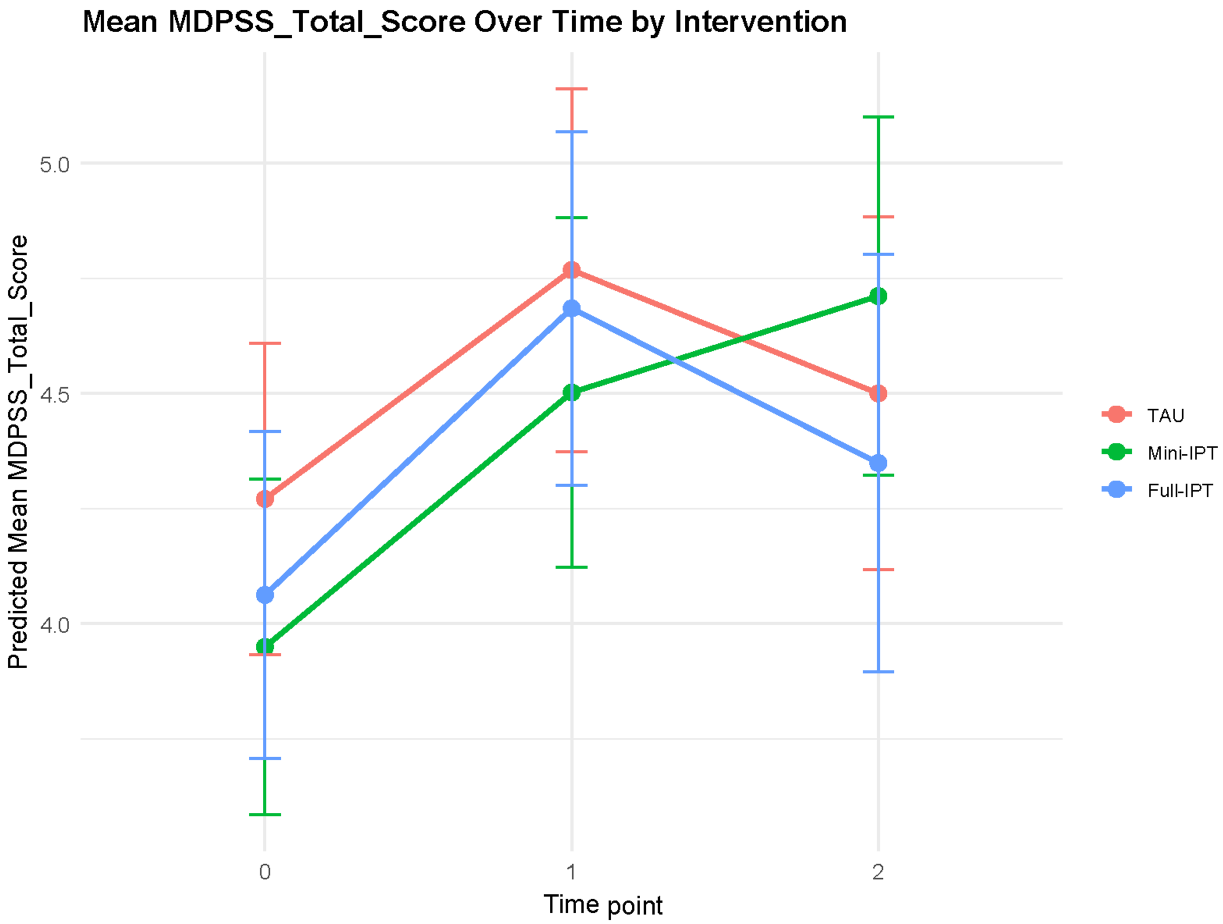

© 2026 Kumar M et al. *JAMA Network Open*.

eFigure 7. Mean HITS Over Time by Intervention

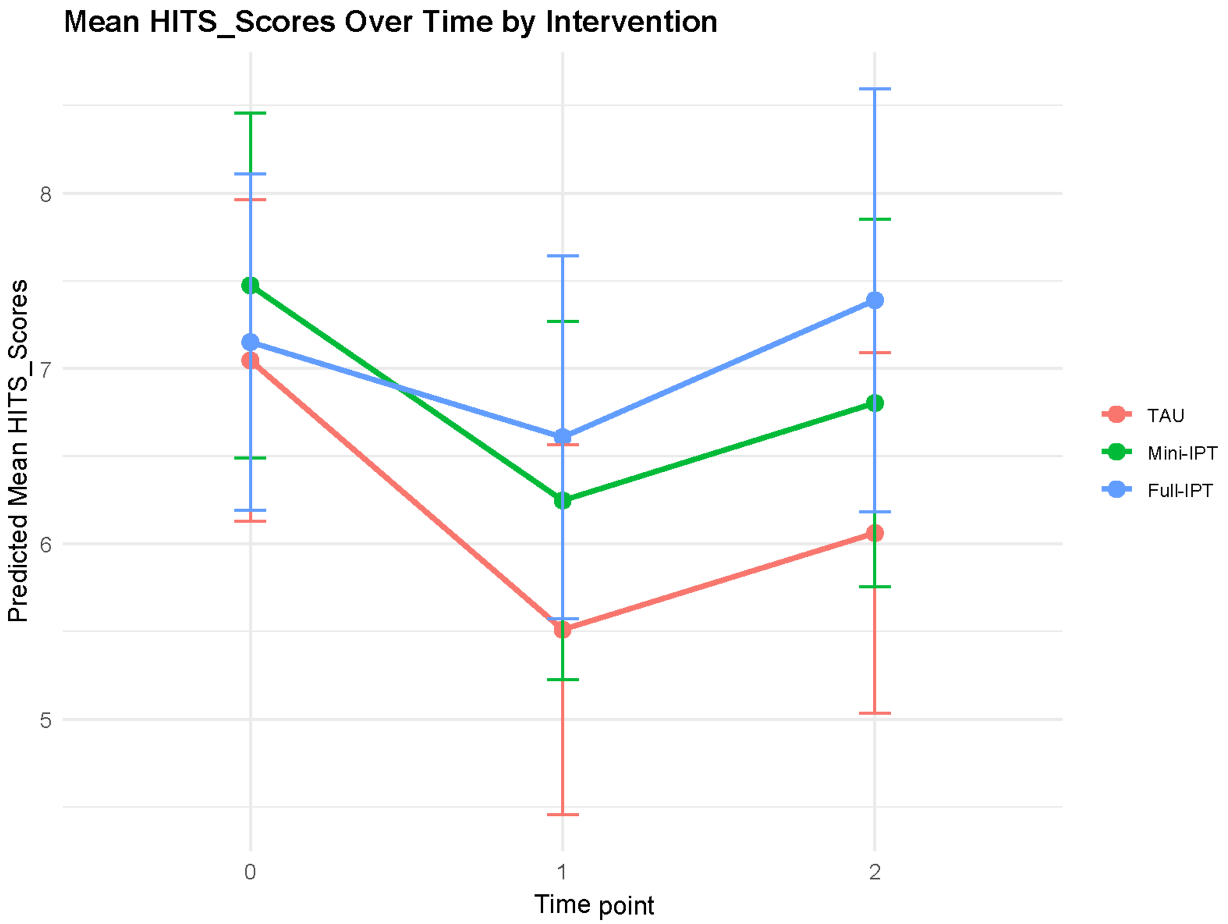

© 2026 Kumar M et al. *JAMA Network Open*.

eFigure 8. Mean General Self-Efficacy Over Time by Intervention

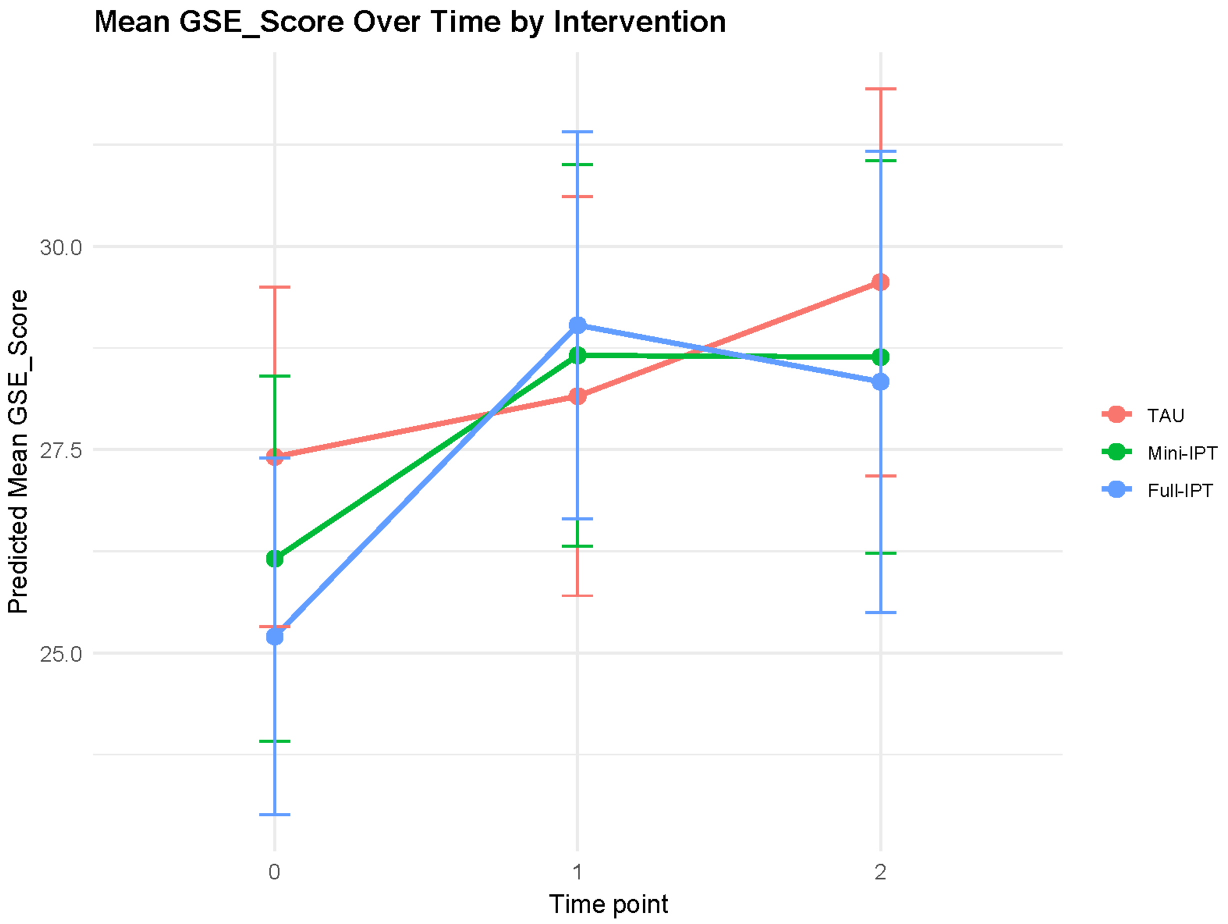

© 2026 Kumar M et al. *JAMA Network Open*.

eFigure 9. Mean CORE-10 Over Time by Intervention

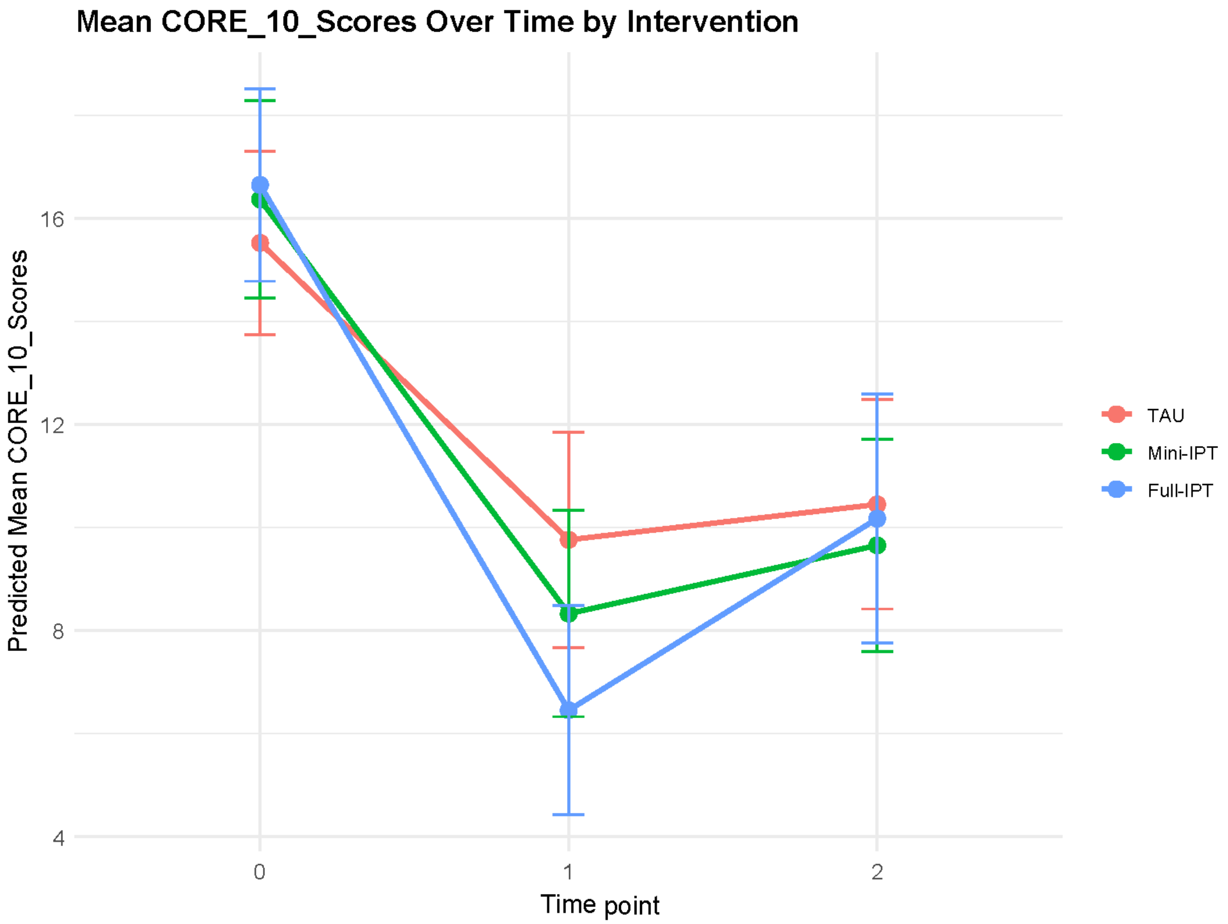

© 2026 Kumar M et al. *JAMA Network Open*.

eFigure 10. Mean COPE Over Time by Intervention

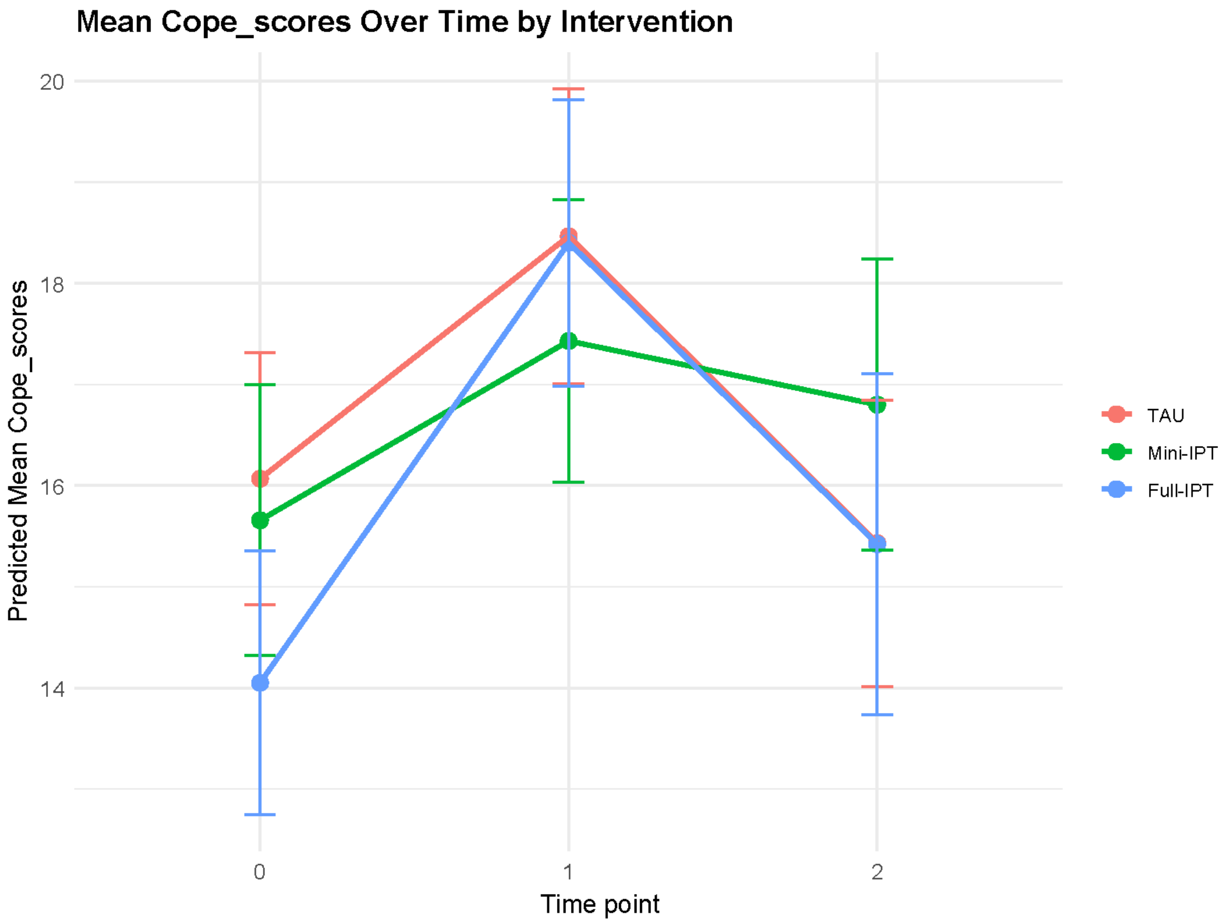

© 2026 Kumar M et al. *JAMA Network Open*.

eFigure 11. Average Depression Scores by Session and Intervention Arm

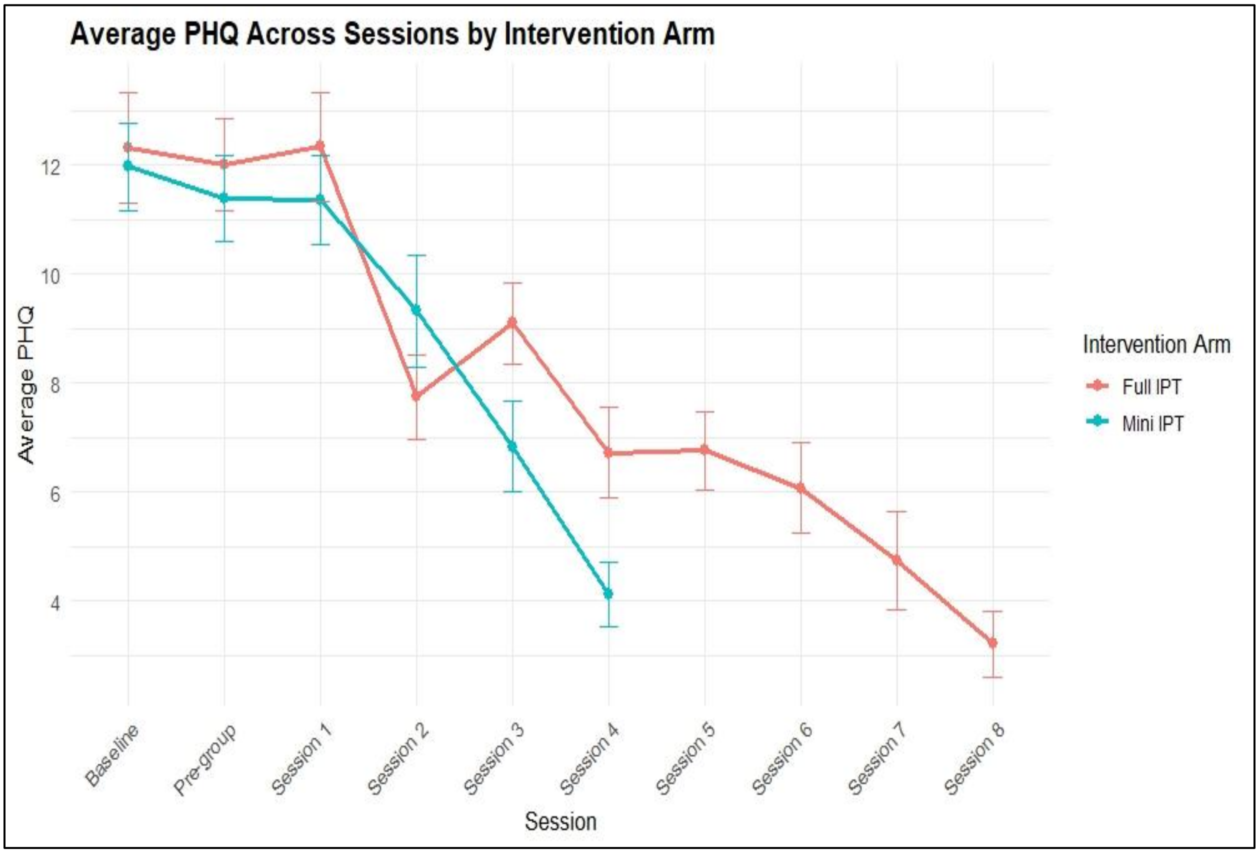

© 2026 Kumar M et al. *JAMA Network Open*.

eFigure 12. Average Depression Scores by Session and Individual

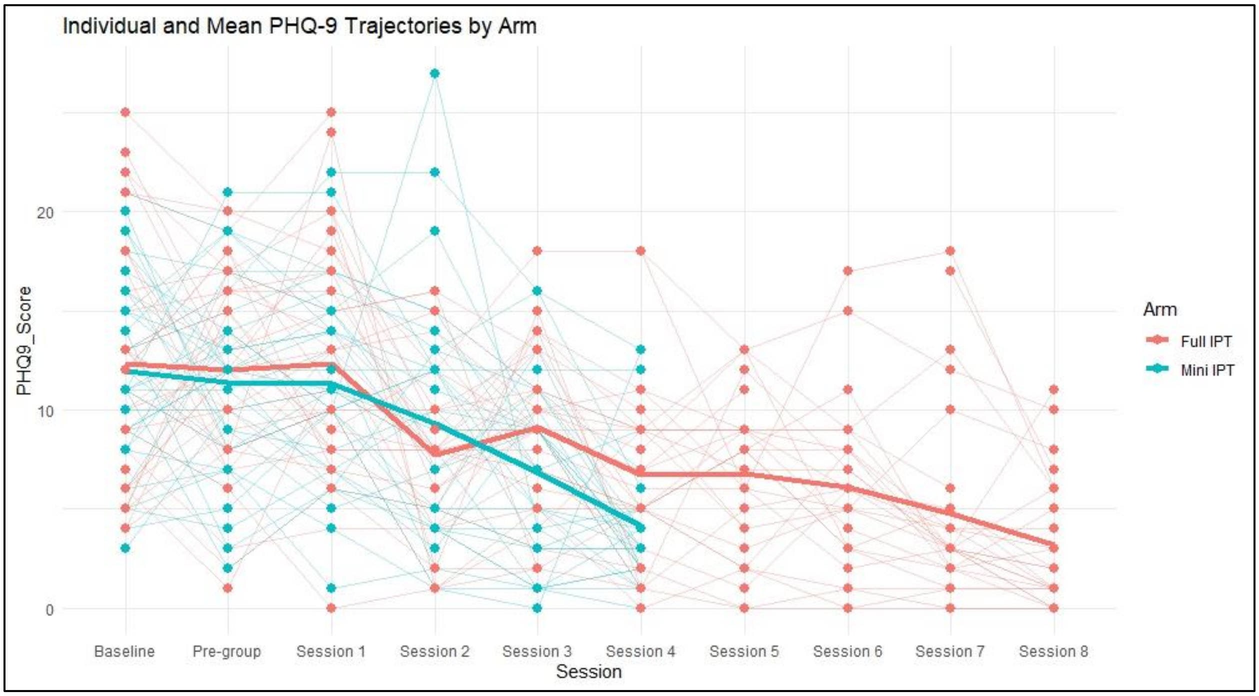

© 2026 Kumar M et al. *JAMA Network Open*.

eReferences.

1. Stata Corp. Stata 18 . Published 2026. Accessed April 11, 2026. https://www.stata.com/stata18/
2. R Core Team. R: A Language and Environment for Statistical Computing. *R Found Stat Comput*. Published online 2024. https://www.r-project.org/
3. Cox JL, Holden JM, Sagovsky R. Detection of Postnatal Depression: Development of the 10-item Edinburgh Postnatal Depression scale. *Br J Psychiatry*. 1987;150(JUNE):782-786. doi:10.1192/bjp.150.6.782
4. Kroenke K, Spitzer RL, Williams JB. The PHQ-9: validity of a brief depression severity measure. *J Gen Intern Med*. 2001;16(9):606-613. doi:10.1046/j.1525-1497.2001.016009606.x
5. Kroenke K, Spitzer RL. The PHQ-9: A New Depression Diagnostic and Severity Measure. *Psychiatr Ann*. 2002;32(9):509-515. doi:10.3928/0048-5713-20020901-06
6. UNICEF. A new tool to measure mental health among adolescents and young people at the population level. Published 2023. Accessed May 24, 2023. https://data.unicef.org/topic/child-health/mental-health/mmap/
7. Carvajal L, Ahs JW, Requejo JH, et al. Measurement of Mental Health Among Adolescents at the Population Level: A Multicountry Protocol for Adaptation and Validation of Mental Health Measures. *J Adolesc Heal*. Published online March 2022. doi:10.1016/j.jadohealth.2021.11.035
8. UW–Madison. McMaster Family Assessment Device (FAD) – Addiction Research Center . Published 2025. Accessed November 8, 2025. https://arc.psych.wisc.edu/self-report/mcmaster-family-assessment-device-fad/
9. Sherin KM, Sinacore JM, Li XQ, Zitter RE, Shakil A. HITS: a short domestic violence screening tool for use in a family practice setting. *Fam Med*. 1998;30(7):508-512. http://www.ncbi.nlm.nih.gov/pubmed/9669164
10. World Health Organization Regional Office for Europe. *Wellbeing Measures in Primary Health Care/the DepCare Project: Report on a WHO Meeting: Stockholm, Sweden, 12–13 February 1998*. World Health Organization. Regional Office for Europe; 1998. https://iris.who.int/handle/10665/349766
11. Schwarzer R, Jerusalem M. General Self-Efficacy Scale. *PsycTESTS Dataset*. Published online January 9, 2012. doi:10.1037/t00393-000
12. Schwarzer R, Jerusalem M. Generalized Self-Efficacy Scale. In: Weinman J, Wright S, Johnston M, eds. *Measures in Health Psychology: A Users Portfolio. Causal Control Beliefs*. Nfer-Nelson; 1995:35-37.
13. Abdin E, Seet V, Jeyagurunathan A, et al. Validation of the 12-item World Health Organization Disability Assessment Schedule 2.0 in individuals with schizophrenia, depression, anxiety, and diabetes in Singapore. *PLoS One*. 2023;18(11):e0294908. doi:10.1371/journal.pone.0294908
14. Barkham M, Bewick B, Mullin T, et al. The CORE-10: A short measure of psychological distress for routine use in the psychological therapies. *Couns Psychother Res*. 2013;13(1):3-13. doi:10.1080/14733145.2012.729069
15. Carver C. You want to measure coping but your protocol's too long: consider the brief COPE. *Int J Behav Med*. 1997;4(1):92-100. doi:0.1207/s15327558ijbm0401_6
16. Zimet GD, Dahlem NW, Zimet SG, Farley GK. The Multidimensional Scale of Perceived Social Support. *J Pers Assess*. 1988;52(1):30-41. doi:10.1207/s15327752jpa5201_2
17. Kilpatrick D, Resnick H, Friedman M. Severity of Posttraumatic Stress Symptoms-Adult * * National Stressful Events Survey PTSD Short Scale (NSESSS). *Am Psychiatr Assoc*. Published online 2013:1. Accessed November 5, 2025. https://www.psychiatry.org/File Library/Psychiatrists/Practice/DSM/APA_DSM5_Severity-of-Posttraumatic-Stress-Symptoms-Adult.pdf

© 2026 Kumar M et al. *JAMA Network Open*.
