# Supplementary material for: Group Interpersonal Psychotherapy for Depression in Perinatal Adolescents in Kenya: A Pilot Randomized Clinical Trial
Source: JAMA Netw Open. 2026 Jun 16;9(6):e2618255. doi: 10.1001/jamanetworkopen.2026.18255 (PMC13273492; doi:10.1001/jamanetworkopen.2026.18255)
Supplement: Supplement 3. — Data Sharing Statement [file jamanetwopen-e2618255-s003.pdf]

Data Sharing Statement

Kumar. Group Interpersonal Psychotherapy for Depression in Perinatal Adolescents in Kenya. *JAMA Netw Open*. Published June 16, 2026. doi:10.1001/jamanetworkopen.2026.18255

Data
Additional Information: PACTR202501888041900.
Data available: Yes
Data types: Deidentified participant data, Data dictionary
How to access data: the data will be provided upon reasonable request from the corresponding author due to vulnerable populations being considered in the study
When available: With publication

Supporting Documents
Document types: Statistical/analytic code, Other (please specify)
Additional Information: study protocol
How to access documents: R/Stata statistical code
When available: With publication

Additional Information
Who can access the data: corresponding author
Types of analyses: non-commercial purpose, open to any secondary analysis of reasonable kinds
Mechanisms of data availability: without investigator support and with a signed data access agreement
